# Accumulation of newly synthesized docosahexaenoic acid plays an essential role in heart regeneration

**DOI:** 10.1093/procel/pwaf062

**Published:** 2025-08-20

**Authors:** Zimu Tang, Zhaoxiang Sun, Chun Yang, Qian Gong, Zirui Liu, Nanhui Chen, Kai Liu, Yong Wang, Ting Zhao, Shengfan Ye, Lenan Zhuang, Jiahao Lin, Wei-Qiang Tan, Jinrong Peng, Jun Chen

**Affiliations:** MOE Key Laboratory of Biosystems Homeostasis & Protection, College of Life Sciences, Cancer Center, Zhejiang University, Hangzhou 310058, China; MOE Key Laboratory of Biosystems Homeostasis & Protection, College of Life Sciences, Cancer Center, Zhejiang University, Hangzhou 310058, China; MOE Key Laboratory of Biosystems Homeostasis & Protection, College of Life Sciences, Cancer Center, Zhejiang University, Hangzhou 310058, China; College of Animal Sciences, Zhejiang University, Hangzhou 310058, China; MOE Key Laboratory of Biosystems Homeostasis & Protection, College of Life Sciences, Cancer Center, Zhejiang University, Hangzhou 310058, China; MOE Key Laboratory of Biosystems Homeostasis & Protection, College of Life Sciences, Cancer Center, Zhejiang University, Hangzhou 310058, China; MOE Key Laboratory of Biosystems Homeostasis & Protection, College of Life Sciences, Cancer Center, Zhejiang University, Hangzhou 310058, China; MOE Key Laboratory of Biosystems Homeostasis & Protection, College of Life Sciences, Cancer Center, Zhejiang University, Hangzhou 310058, China; MOE Key Laboratory of Biosystems Homeostasis & Protection, College of Life Sciences, Cancer Center, Zhejiang University, Hangzhou 310058, China; MOE Key Laboratory of Biosystems Homeostasis & Protection, College of Life Sciences, Cancer Center, Zhejiang University, Hangzhou 310058, China; College of Animal Sciences, Zhejiang University, Hangzhou 310058, China; MOE Key Laboratory of Biosystems Homeostasis & Protection, College of Life Sciences, Cancer Center, Zhejiang University, Hangzhou 310058, China; Department of Plastic Surgery, Sir Run Run Shaw Hospital, Zhejiang University School of Medicine, Hangzhou 310016, China; College of Animal Sciences, Zhejiang University, Hangzhou 310058, China; MOE Key Laboratory of Biosystems Homeostasis & Protection, College of Life Sciences, Cancer Center, Zhejiang University, Hangzhou 310058, China; Department of Plastic Surgery, Sir Run Run Shaw Hospital, Zhejiang University School of Medicine, Hangzhou 310016, China

**Keywords:** heart regeneration, zebrafish, mouse, DHA, PPARD

## Abstract

Adult zebrafish and neonatal mice can fully regenerate their hearts after partial amputation through the proliferation of preexisting cardiomyocytes (CMs). However, the adult mammalian heart has limited regenerative capability following cardiac damage. The reason for this phenomenon remains elusive. Here, we find that docosahexaenoic acid (DHA) is accumulated only in the injured hearts of zebrafish and neonatal mice, but not of adult mice, which coincides with the upregulation of DHA synthesis genes in CMs, fibroblasts, and macrophages near the injury areas. Inhibition of *Fads2*, a DHA synthesis enzyme, impairs heart regeneration in both zebrafish and neonatal mice. Injection of DHA remodels the transcriptome from injury response to regeneration response and improves cardiac function in adult mice after myocardial infarction. Interestingly, DHA facilitates CM proliferation but inhibits fibrosis and inflammation. Mechanistically, only DHA, but not oleic acid (OA), can trigger the peroxisome proliferator-activated receptor d (PPARD) to bind to the promoter regions of heart regeneration-related genes, such as *Mef2d, Phlda3*, and *Txndc5*, to regulate their expression. Molecular docking, molecular dynamics simulations, and mutagenesis experiments suggest that DHA binds to PPARD in a distinct manner compared to OA, which may help explain their differing abilities to influence the expression of heart regeneration genes. Our findings demonstrate that the DHA signal plays an essential and evolutionarily conserved role in heart regeneration and provide a therapeutic potential for myocardial infarction.

## Introduction

The neonatal mouse heart is capable of regeneration up to 15% of the cardiomyocytes (CMs) during the first 7 days after birth. However, this regenerative capacity is lost soon after (Porrello et al., 2011). The limited regenerative capability of mammalian hearts following cardiac damage is a major barrier in cardiovascular medicine and often leads to heart failure. Unlike adult mammals, adult zebrafish *(Danio rerio)* have full capacity of cardiac regeneration following ventricular resection (Poss et al., 2002). Cell lineage tracing experiments revealed that CMs from the subepicardial ventricular layer dedifferentiated into *gata4*-positive CMs to proliferate and the new CMs slowly replaced the scar tissue in the area of injury (Jopling et al., 2010; Kikuchi et al., 2010).

Several lines of evidence have demonstrated that CM polyploidization is a barrier to heart regeneration (Gonzalez-Rosa et al., 2018; Hirose et al., 2019; Patterson et al., 2017). In zebrafish, about 99% of CMs remain mononuclear and diploid throughout life (Gonzalez-Rosa et al., 2018). In contrast, mammalian CMs mostly become binucleated and/or polyploid after birth. In mice, polyploidization occurs during the first postnatal week and results in about 78% of CMs becoming polyploid, coinciding with their loss of proliferative and regenerative capacity. However, mammalian liver is also composed of high percentages of polyploid hepatocytes (about 90% in mouse). Both polyploid and diploid hepatocytes can contribute to the liver regeneration (Lin et al., 2020; Wilkinson et al., 2019). Therefore, the major challenge is how to stimulate polyploid CMs to undergo cytokinesis or endoreplication to compensate for the loss of cardiac mass in adult mammals.

A recent study has identified AP-1-enriched regeneration-responsive enhancers (RREs) by comparison of the epigenetic and transcriptional changes during caudal fin regeneration between two teleosts: zebrafish and African killifish (Wang et al., 2020). The AP-1-enriched RREs are required for both caudal fin and heart regeneration, and function to activate a regenerative response that includes both injury and regeneration. Through the course of evolution and speciation, the RREs are changed, resulting in dissociation of regeneration (loss) and injury (retention) responses in mammals (mouse and human). However, this mechanism is difficult to explain why the liver of mammals and the heart of neonatal mice can still fully regenerate.

During development, the embryonic heart mainly utilizes glucose for ATP production (Piquereau and Ventura-Clapier, 2018). As the heart grows, CMs exhibit metabolic changes and more than 95% of cardiac ATP is generated from fatty acid oxidation in the adult heart (Doenst et al., 2013). Interestingly, the loss of cardiac regenerative capacity in mammalian hearts coincides with the metabolic switch of energy source. Lipid is not only served as an energy supply but also is composed of many biology macro molecules such as membranes. However, excessive of some lipids in heart, such as ceramides, diacylglycerols, long-chain acyl-CoAs, and acylcarnitines lead to lipotoxicity, which may cause cardiomyopathy (D’Souza et al., 2016). Abrogating transportation of fatty acids from cytoplasm to mitochondria by inactivation of *Cpt1b* allows heart regeneration in adult mice after ischemia–reperfusion injury (Li et al., 2023) and inhibiting fatty acid utilization promotes cardiomyocyte proliferation in postnatal mice (Cardoso et al., 2020). Therefore, how lipid metabolic pathway participates in heart regeneration and whether some lipids can determine heart regeneration capacity as a signal still remains unknown. Recent studies have demonstrated that α-ketoglutarate promotes CM proliferation through either KDM5-mediated demethylation of H3K4me3 (Li et al., 2023) or KDM6-mediated demethylation of H3K27me3 (Shi et al., 2024), suggesting some metabolites function as a signal molecule to regulate heart regeneration.

In this report, we reveal that the accumulation of docosahexaenoic acid (DHA) around the injury site only occurs in the injured hearts of zebrafish and neonatal mice, but not of the adult mice, and DHA contributes to the difference in their heart regeneration capacities. DHA-injection in adult mice after myocardial infarction (MI) remodels the transcriptome from injury response to regeneration response, thus induces the CM proliferation, reduces scarring, and improves cardiac function. Previous longitudinal prospective cohort studies have demonstrated that DHA and eicosapentaenoic acid (EPA) are associated with a lower risk of developing cardiovascular disease, especially coronary heart disease, MI, and cardiovascular mortality (1999). Therefore, DHA supplementation provides great potential for the therapy of heart failure diseases.

## Results

### Lipids are accumulated around the injury site of zebrafish heart

**Figure 1.**
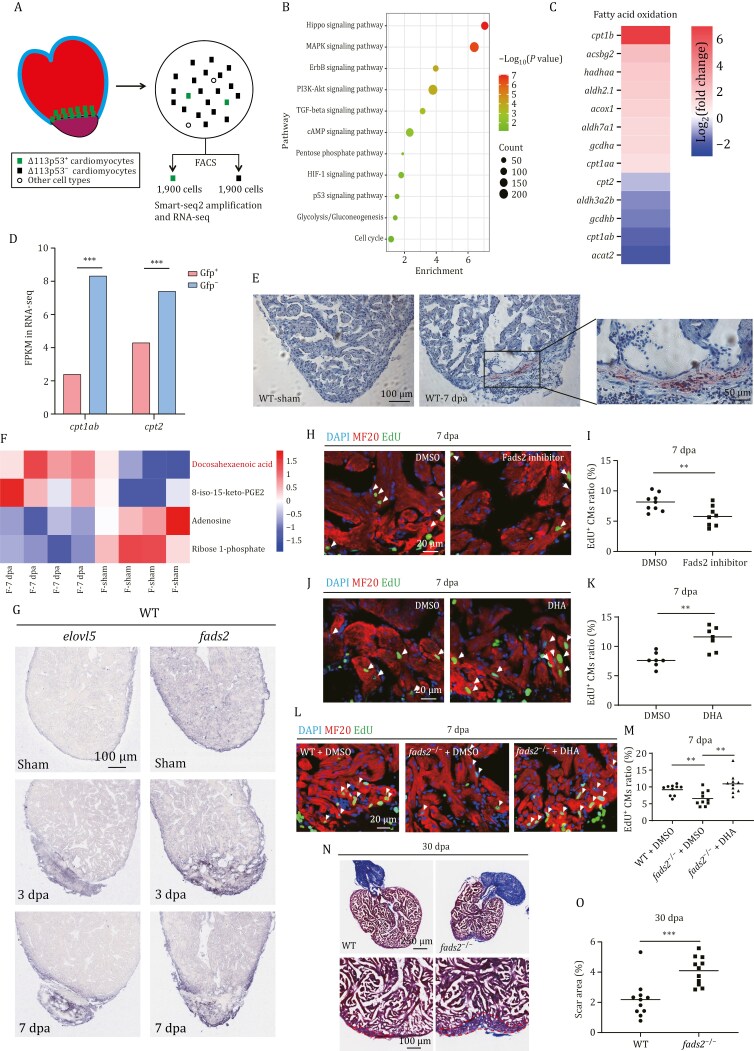
**DHA is the lipid metabolite that increases the most in zebrafish injury hearts to promote heart regeneration.** (A) Diagram showing isolation of ∆ 113p53^+^ CMs from 6 injury hearts of *Tg*(*Δ113p53*:*GFP*) transgenic zebrafish at 7 dpa. The dissociated CMs were sorted with FACS for GFP^+^ and GFP^−^ CMs. About 1,900 GFP^+^ and 1,900 GFP^−^ CMs were pooled together, respectively, followed by RNA-seq with Smart-seq2 technique. (B) KEGG analysis of differentially expressed genes (DEGs) between ∆ 113p53^+^ and ∆ 113p53^−^ CMs to show significantly enriched pathways related to CM proliferation and metabolism. (C) Heatmaps of DEGs related to fatty acid oxidation in ∆ 113p53^+^ and ∆ 113p53^−^ CMs. (D) FPKMs of *cpt1ab* and *cpt2* in ∆ 113p53^+^ and ∆ 113p53^−^ CMs from RNA-seq. (E) Representative images of Oil Red O staining in zebrafish injury and sham hearts at 7 dpa. *n*: 10 hearts/sample; Scale bars: 100 μm or 50 μm, respectively, for left and right panels. (F) Hierarchical clustering analysis of differential metabolites between zebrafish sham and injury hearts at 7 dpa. Each treatment had 4 replicates. About 15–25 ventricles in each replicate were pooled together to reach 5 mg weight and subjected for LC-MS analysis. (G) RNA *in situ* hybridization was performed with the DIG-labeled probe to detect both *elovl5* and *fads2* on cryosections of zebrafish WT hearts at sham, 3, and 7 dpa. The representative picture was taken from 10 hearts in each group. Scale bar: 100 μm. (H and J) Immuno-staining of MF20 (in red) and EdU incorporation assay (in green) of zebrafish WT injury hearts with different treatments at 7 dpa. The zebrafish with heart resection were intraperitoneally injected with DMSO (the injection control), Fads2 inhibitor (H), and DHA (J). Scale bar: 20 μm. (I and K) Statistical analyses of EdU^+^ CMs in (H) and (J). The number of EdU^+^ CMs on heart sections was presented as the percentage of the total MF20^+^ cells at the resection sites. Each dot represents an individual heart. *n*: 8–9 hearts/sample. (L)Immuno-staining of MF20 (in red) and EdU incorporation assay (in green) of WT and *fads2*^−/−^ injury hearts injected with DMSO or DHA at 7 dpa. Scale bar: 20 μm. (M) Statistical analyses of EdU^+^ CMs in (L). The number of EdU^+^ CMs on heart sections was presented as the percentage of the total MF20^+^ cells at the resection sites. Each dot represents an individual heart. *n*: 10 hearts/sample. (N) Fibrin clot stained with Masson’s trichrome on the cryosections of WT and *fads2*^−/−^ injury hearts at 30 dpa. The scar areas were magnified in the lower panel and indicated by the red dotted lines. Scale bar: 250 μm or 100 μm, respectively, for upper and lower panels. *n*: 11 hearts/sample. (O) Statistical analyses of scar areas in (N). The average scar area with fibrin clots on heart sections was presented as the percentage of the total ventricular area. Each dot represents an individual heart. The experiments were repeated independently for at least 2–3 times with similar results. Statistical analysis was performed by Student’s two-tailed unpaired *t* test in GraphPad Prism 8. The *P* values were represented by ns and asterisks. ns, *P* > 0.05; *P < 0.05; **P < 0.01; ***P < 0.001.

After zebrafish heart injury, CMs from the subepicardial ventricular layer dedifferentiate into *gata4*-positive CMs and migrate to the injury site to proliferate (Kikuchi et al., 2010). The area around the injury site is considered a proliferating border zone to contribute to zebrafish heart regeneration. Previously, we used *Δ113p53* (an N-terminal truncated isoform of p53) promoter to generate a *Tg*(*Δ113p53*:*GFP*) transgenic zebrafish line (Chen et al., 2009). The expression of GFP in the transgenic fish faithfully mimics the transcription of endogenous *Δ113p53* to be activated in the border zone of zebrafish heart beginning at 7 dpa. These GFP^+^ CMs enter cell cycle to participate in CM renewal (Ye et al., 2020). To explore the gene expression differences between proliferating and non-proliferating CMs, we surgically removed ~15% of ventricular CMs from *Tg*(*Δ113p53*:*GFP*) transgenic zebrafish hearts. CMs of the amputated hearts from 6 individuals at 7 dpa were dissociated and sorted with FACS for GFP^+^ and GFP^−^-CMs. Approximately 1,900 GFP^+^ and 1,900 GFP^−^-CMs were pooled, respectively, followed by RNA-seq with Smart-seq2 technique (Picelli et al., 2014) (Fig. 1A). Total 9,001 up-regulated and 2,924 down-regulated genes were identified in GFP^+^ CMs (|log_2_(fold change)| ≥ 1, *P*_*adj*_ < 0.05) (Fig. S1A). Using single-cell sequencing technology, a previous study has identified 752 upregulated genes in proliferating CMs (Honkoop et al., 2019). Intersection analysis on the upregulated genes showed that 296 genes in the GFP^+^ cells overlapped with genes identified in single-cell sequencing, indicating GFP^+^ cells represent proliferating CMs (Fig. S1B). As expected, the fragments per kilobase of exon model per million mapped fragments (FPKMs) of *Δ113p53*, as well as two cell cycle-related genes *cdc6* and *wee1* were extremely higher in GFP^+^ cells than those in GFP^−^ cells, which was validated by qRT-PCR (Fig. S1D and S1E). To evaluate the quality of the RNA-seq data, we randomly selected 20 genes, including 6 upregulated, 6 down-regulated, and 8 no-change genes to perform qRT-PCR. The correlation analysis showed a highly significant correlation (*P < *0.01, coefficient = 0.9567) between the results of qRT-PCR and RNA-seq (Fig. S1C). *In situ* hybridization with 3 upregulated genes (*aldocb*, *pgm1*, and *hif1ab*) in GFP^+^ CMs also showed that the expression of three genes was induced around injury sites at 7 dpa (Fig. S1I). The results demonstrated that the GFP^+^ cells were the proliferating CMs.

In consistent with previous studies (Chablais and Jazwinska, 2012; von Gise et al., 2012; Jopling et al., 2012a, b; Gemberling et al., 2015; Flinn et al., 2019; Fukuda et al., 2020; Shoffner et al., 2020; Mukherjee et al., 2021; Tahara et al., 2021; Tan et al., 2022), KEGG pathway analysis of the differentially expressed genes (DEGs) showed that 11 signal pathways involved in heart regeneration including Hippo, MAPK, ERBB, PI3K-AKT, TGF-β, cAMP, pentose phosphate pathway (PPP pathway), Hif-1, p53, glycolysis/gluconeogenesis and cell cycle were enriched (Fig. 1B). For instance, among 51 cell cycle related and DNA replication DEGs, 34 genes were upregulated and 17 DEGs were downregulated; among 41 DEGs related to glycolysis and glucose oxidation, 37 genes were upregulated and 4 genes were downregulated; among 26 DEGs related to the PPP pathway and tricarboxylic acid cycle (TCA cycle), 20 genes were upregulated and 6 genes were downregulated. In contrast, most of the DEGs related to oxidative phosphorylation (29/35) were downregulated (Fig. S1F and S1G). Interestingly, among 13 DEGs involved in fatty acid oxidation, 8 genes were upregulated and 5 genes were downregulated (Fig. 1C). GSEA analysis showed that there were no significant changes in fatty acid oxidation in proliferation CMs (Fig. S1J), which was somehow different from previous studies (Honkoop et al., 2019). We found that two of the five downregulated genes (*cpt1ab* and *cpt2*) were responsible for the transportation of fatty acids from cytoplasm to mitochondria, which *cpt1ab* was confirmed by qRT-PCR (Figs. 1D and S1H). The results suggested that the intracellular transportation of fatty acids was probably reduced in proliferating CMs. To investigate the consequences of downregulation of fatty acid transportation genes, we performed oil-red O staining in regenerating zebrafish heart. Interestingly, lipid accumulation in a diffusion manner was observed around the injury site at 3 and 7 dpa, and disappeared at 30 dpa (Figs. 1E and S2A–D), raising a question whether lipid accumulation might play a role in heart regeneration.

### Knockout of *cpt1ab* increases lipid accumulation around injury area and promotes zebrafish heart regeneration

To address the role of lipid accumulation in heart regeneration, we manipulated lipid transportation from cytoplasm to mitochondria by the abdominal injection of a Cpt1a inhibitor (Etomoxir) (Schmidt-Schweda and Holubarsch, 2000) or a Cpt1a activator (Baicalin) (Dai et al., 2018), during 4–6 dpa. The results showed that the percentage of EdU^+^ CMs at 7 dpa was significantly increased in Etomoxir-injected hearts, and significantly decreased in Baicalin-injected hearts, compared to that in DMSO-injected control hearts (Fig. S3A–D).

To confirm the function of Cpt1a, we generated a zebrafish *cpt1ab* mutant with 4 bp deletion, resulting in a premature termination codon (PTC) at 528 aa (Fig. S3E). The qRT-PCR showed that the transcript of *cpt1ab* was obviously decreased in the *cpt1ab*^−/−^ mutant at different developmental stages and in *cpt1ab*^−/−^ mutant injury heart (Fig. S3F), suggesting the mutant mRNA with a PTC was degraded. The *cpt1ab*^−/−^ zebrafish mutant developed relatively normal and fertile. Similar to Etomoxir-injected hearts, the percentage of EdU^+^ CMs at 7 dpa was significantly increased in *cpt1ab*^−/−^ zebrafish mutant hearts, compared to that in WT hearts (Fig. S3G and S3I). The masson trichrome staining also showed that the scar area at 30 dpa was significantly smaller in the *cpt1ab*^−/−^ mutant hearts than that in WT hearts (Fig. S3H and S3J). The results demonstrate that the depletion of *cpt1ab* promotes zebrafish heart regeneration, which is consistent with the observation in *Cpt1b* knockout mice of a recent publication (Li et al., 2023). In this study, the authors have demonstrated that knockout of *Cpt1b* stimulates CM proliferation, allowing heart regeneration after ischemia-reperfusion injury in adult mice. However, two studies from zebrafish have revealed that zebrafish *cpt1b* knockout mutant fish display defects in ventricle regeneration and CM proliferation (Cheng et al., 2024; Zhao et al., 2024), which are contradictory to our zebrafish *cpt1ab* and mouse *Cpt1b* mutants. Interestingly, our Smart-seq data showed that in contrast to the downregulation of *cpt1ab* and *cpt2* in proliferating CMs, the expression of *cpt1b* in the proliferating CMs was significantly increased (Fig. 1C). The differential expression regulation might explain the function discrepancy between zebrafish *cpt1ab* and *cpt1b* mutants and also suggested that zebrafish *cpt1ab* and *cpt1b* play different roles in fatty acid transportation.

Oil-red O staining showed that the lipid accumulation at 7 dpa was significantly increased in *cpt1ab*^−/−^ zebrafish mutant hearts, but decreased in Baicalin-injected hearts (Fig. S3K–N). Taken together, the results suggest that *cpt1ab* knockout may lead to intracellular lipid accumulation to promote the heart regeneration. However, it does not exclude the possibility that *cpt1ab* knockout may also result in an increase in glucose utilization to promote the heart regeneration.

### DHA is the metabolite that increases most significantly in the zebrafish injury hearts to promote heart regeneration

Next, we were wondering if there were any specific fatty acids in lipid accumulation to function as a signal to promote heart regeneration. For this purpose, we performed metabonomics through liquid chromatography-mass spectrometry (LC-MS) to compare sham hearts and injury hearts at 7 dpa. Each group had four replicates, each of which contained 15–25 hearts (about 5 mg in weight). A total of 5,456 metabolites were detected, and 202 metabolites were precisely mapped by the negative ion (NEG) model. The orthogonal partial least squares discriminant analysis (OPLS-DA) score plots showed that four replicates in each group were nicely distinguished in a 2D plot (Fig. S4A). Among 5,456 detected metabolites, 46 metabolites were significantly up-regulated and 62 were significantly down-regulated (Fig. S4B). Of the 202 mapped metabolites, only 4 metabolites were identified to be significantly changed in the injured hearts, including two decreased metabolites (adenosine and ribose-1-phosphate) and two increased metabolites (DHA and 8-iso-15-keto-PGE2) (Fig. 1F). The two increased metabolites were fatty acids or derivatives. Among 202 mapped metabolites, 59 were lipids or derivatives. In addition to DHA and 8-iso-15-keto-PGE2, other 6 lipids were also increased but not significantly in the injured hearts (Fig. S4C). Interestingly, the FPKMs of *fatty acid desaturase 2* (*fads2*) and *elongation of very long-chain fatty acids protein 5* (*elovl5*), two genes encoding enzymes responsible for the synthesis of DHA, were significantly higher in proliferation CMs (Fig. S4D). *In situ* hybridization also showed that the expression of these two genes was induced at around the resection site of the injured heart at 3 and 7 dpa (Fig. 1G). To detect the precise expression localization of these two genes, we performed double *in situ* hybridization by combining *cmlc2* (a CM marker) with *fads2* or *elovl5* probes. The results revealed that injury-induced expression of *fads2* and *elovl5* not only appeared in the injury area but also co-localized with CMs around the injury boundary at 7dpa (Fig. S4E and S4F). Previous studies have revealed that PGE2 functions in wounding response and promotes heart regeneration (FitzSimons et al., 2020). All of these results demonstrate that there might be a close correlation between the lipid accumulation and heart regeneration.

As DHA was the fatty acid that most significantly increased in heart regeneration (2.3 times), it raised a question of whether the DHA accumulation functions in heart regeneration. We abdominally injected a Fads2 inhibitor (SC-26196) (Harmon et al., 2003) or DHA into WT zebrafish during 4–6 dpa. The results showed that the percentage of EdU^+^ CMs at 7 dpa was significantly decreased in Fads2 inhibitor-injected hearts, and significantly increased in DHA-injected hearts, while the proportion of EdU^+^ other cell types was not significantly changed in DHA-injected injured hearts (Fig. S5E and S5F), compared to DMSO-injected control hearts (Fig. 1H–K). Notably, the proportion of EdU^+^ CMs in the Sham hearts was not significantly increased by DHA injection, compared to DMSO-injected Sham hearts (Fig. S5G and S5H).

During zebrafish heart regeneration, a large blood clot (most of them are erythrocytes and platelets) forms at the resection site after a few seconds of profuse bleeding from the ventricular lumen. These blood cells will be replaced by fibrin beginning at 3 dpa. CMs surround, penetrate, and finally replace the fibrin clot from 7 to 30 dpa (Koth et al., 2020). The area of the injury containing the fibrin clot is a critical parameter for evaluating the quality of heart regeneration. Interestingly, we found that the percentage of blood clot area in wounded hearts at 7 dpa was significantly decreased in DHA-injected hearts, compared to those in DMSO-injected control hearts (Fig. S5A and S5B). Masson-trichrome staining showed that the fibrin clot area at 7 dpa was significantly smaller in DHA-injected hearts than that in DMSO-injected control hearts (Fig. S5C and S5D).

A previous study has reported that a *fads2*^−/−^ zebrafish mutant develops relative normal and is fertile, but has an increase in α-linolenic acid (ALA) and a decrease in DHA, compared to WT fish (Zhao et al., 2020). Next, we used the zebrafish *fads2* mutant to confirm the role of DHA accumulation in heart regeneration. DHA or DMSO was abdominally injected into *fads2*^−/−^ zebrafish during 4–6 dpa. The results showed that the percentage of EdU^+^ CMs at 7 dpa was significantly decreased in DMSO-injected *fads2*^−/−^ mutant hearts, compared to those in DMSO-injected WT hearts (Fig. 1L and 1M). Masson-trichrome staining also showed that the fibrin clot area at 30 dpa was significantly larger in *fads2*^−/−^ mutant hearts than that in WT hearts (Fig. 1N and 1O). Interestingly, DHA injection restored the percentage of EdU^+^ CMs at 7 dpa in the *fads2*^−/−^ mutant injured hearts to similar levels of those in the WT injured hearts (Fig. 1L and 1M).

The results demonstrate that the DHA accumulation plays an essential role in zebrafish heart regeneration.

### DHA promotes zebrafish heart regeneration through *ppardb*

**Figure 2.**
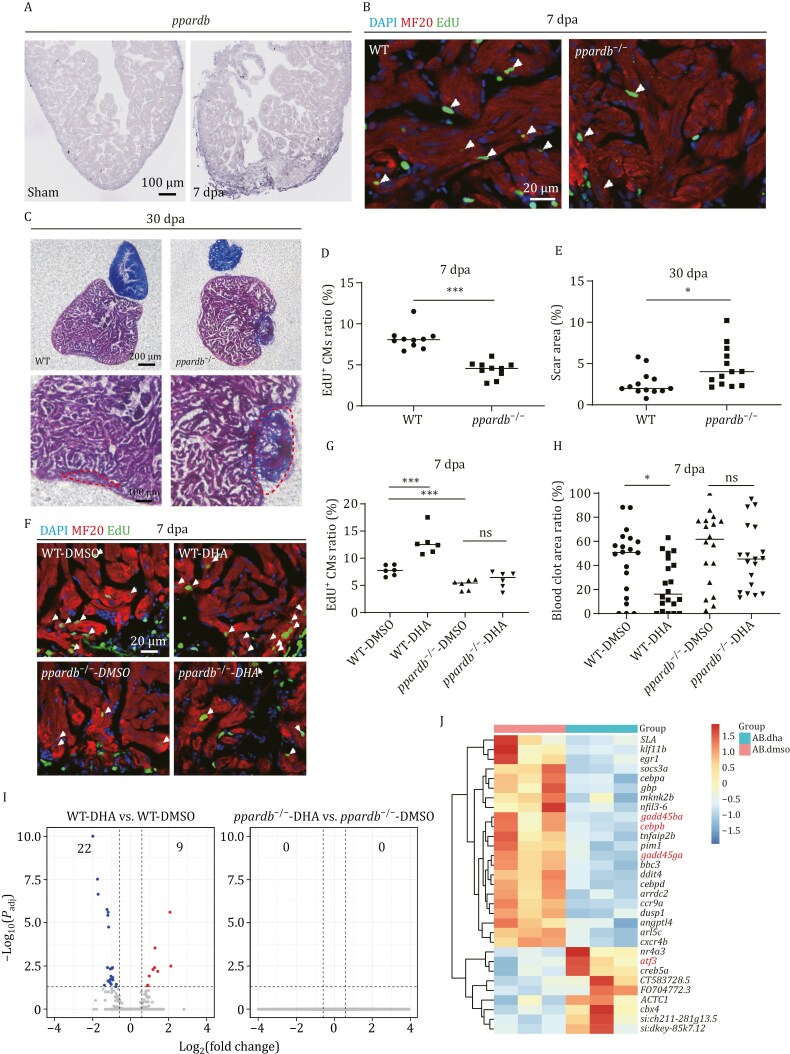
**DHA promotes zebrafish heart regeneration via *ppardb* to regulate the expression of heart regeneration-related genes.** (A) RNA *in situ* hybridization was performed with the DIG-labeled probe to detect *ppardb* on cryosections of zebrafish WT hearts at sham and 7 dpa. The representative picture was taken from 8–10 hearts in each group. Scale bar: 100 μm. (B) Immuno-staining of MF20 (in red) and EdU incorporation assay (in green) of WT and *ppardb*^−/*−*^ injury hearts at 7 dpa. *n*: 10 hearts/sample; Scale bar: 20 μm. (C) Fibrin clot stained with Masson’s trichrome on the cryosections of WT and *ppardb*^−/−^ injury hearts at 30 dpa. The scar areas were magnified in the lower panel and indicated by the red dotted lines. *n*: 13 hearts/sample; Scale bar: 200 μm. (D and E) Statistical analyses of EdU^+^ CMs and scar areas in (B) and (C). The number of EdU^+^ CMs on heart sections was presented as the percentage of the total MF20^+^ cells at the resection sites (B). Average scar area with fibrin clots on heart sections was presented as the percentage of the total ventricular area (C). Each dot represents an individual heart. (F) Immuno-staining of MF20 (in red) and EdU incorporation assay (in green) of WT and *ppardb*^−/−^ injury hearts injected with DMSO or DHA at 7 dpa. Scale bar: 20 μm. (G) Statistical analyses of EdU^+^ CMs in (F). The number of EdU^+^ CMs on heart sections was presented as the percentage of the total MF20^+^ cells at the resection sites. Each dot represents an individual heart. *n*: 6 hearts/sample. (H) Statistical analysis of blood clot areas in WT and *ppardb*^−/−^ injury hearts injected with DMSO or DHA at 7 dpa (Fig. S6D). The average area with blood clot on an injury heart was presented as the percentage of the total injury area. Each dot represents an individual heart. *n*: 18–20 hearts/sample. (I) Volcano plots showing the DEGs (|fold change| ≥ 1.5, *P*_adj_ < 0.05) of injury hearts at 7 dpa in two comparisons: WT-DHA versus WT-DMSO, and *ppardb*^−/−^-DHA versus *ppardb*^−/−^-DMSO. The WT and *ppardb*^−/−^ zebrafish with heart resection were intraperitoneally injected with DMSO (the injection control) and DHA. Each treatment had three replicates. Each replicate had 6 injury hearts at 7 dpa. The total RNA from 6 injury hearts of each replicate was subjected for RNA-seq analysis. (J) Heatmap of DEGs in 7 dpa injury hearts of WT zebrafish injected with DHA. Genes related to the heart repair or proliferation were in red. The experiments were repeated independently for at least 2–3 times with similar results. Statistical analysis was performed by Student’s two-tailed unpaired *t* test in GraphPad Prism 8. The *P* values were represented by ns and asterisks. ns, *P* > 0.05; *P < 0.05; ***P < 0.001.

A previous study has demonstrated that the supplementation of DHA and EPA promotes B-lymphocyte cell proliferation (Verlengia et al., 2004). DHA and EPA can bind to nuclear receptors such as peroxisome proliferator-activated receptors (PPAR) (PPARα, PPARβ/δ, and PPARγ) and Retinoid X Receptor (RXR) to function as an agonist (Han et al., 2017). Furthermore, activation of PPARδ promotes heart regeneration in both zebrafish and mice (Magadum et al., 2017). There are two PPARδ homologous genes in zebrafish: *pparda* and *ppardb*. From RNA-seq data, we found that both *pparda* and *ppardb* were upregulated in GFP^+^ CMs. However, the total FPKM of *ppardb* was about 3 times that of *pparda* in GFP^+^ CMs (Fig. S6A). *In situ* hybridization also showed that the expression of *ppardb* was induced around wounded sites at 7 dpa (Fig. 2A). Therefore, we generated a zebrafish *ppardb* mutant with 11 bp deletion, resulting in a premature termination codon (PTC) at 246 aa (Fig. S6B). The qRT-PCR showed that the transcript of *ppardb* was significantly decreased in *ppardb*^−/−^ mutant embryos at 1, 3, and 5 dpf (Fig. S6C), suggesting that the mutant mRNA with a PTC was degraded. Similar to the *cpt1ab*^−/−^ mutant, the *ppardb*^−/−^ zebrafish mutant developed relatively normal and fertile. However, the percentage of EdU^+^ CMs at 7 dpa was significantly decreased in *ppardb*^−/−^ zebrafish mutant hearts, compared to that in WT hearts (Fig. 2B and 2D). The masson trichrome staining also showed that the scar area at 30 dpa was significantly larger in the *ppardb*^−/−^ mutant hearts than that in WT hearts (Fig. 2C and 2E). Thus, similar to the previous study, our results also demonstrate that *ppardb* is required for zebrafish heart regeneration.

To investigate the essential role of DHA in heart regeneration in context of *ppardb*, we abdominally injected DHA into WT and *ppardb*^−/−^ mutant during 4-6 dpa. The results showed that the injection of DHA significantly increased the percentage of EdU^+^ CMs and decreased the percentage of blood clot area at 7 dpa in WT hearts, but not in *ppardb*^−/−^ mutant hearts, compared to those in respective DMSO-injection controls (Figs. 2F–H and S6D).

To explore how the DHA/ppardb axis promotes zebrafish heart regeneration, we performed RNA-seq to compare transcriptomes between DHA- and DMSO-injected injury hearts at 7 dpa in WT or *ppardb*^−/−^ mutant background. DEG analysis (|log_2_(fold change)| ≥ 0.68, *P*_adj_ < 0.05) showed that DHA injection altered gene expression only in the WT injured hearts (22 genes down-regulated and 9 genes upregulated), but not in the *ppardb*^−/−^ mutant injured hearts, which was consistent with the results that DHA significantly increased heart regeneration in WT, but not in *ppardb*^−/−^ (Fig. 2I). Among these DEGs in DHA treatment, several genes (*atf3* upregulated; *gadd45ba*, *gadd45ga*, and *cebpb* downregulated) have been reported to play a role in heart repair or regeneration (Fig. 2J). For instance, overexpression of ATF3 in cardiac fibroblasts in response to hypertensive stimuli protects the heart by suppressing p38 MAPK signaling (Li et al., 2017). The activation of p38 MAPK impaired CM proliferation in either fetal mice or zebrafish (Engel et al., 2005; Jopling et al., 2012). GADD45 is a cell cycle repressor that terminates cell proliferation by down-regulating *PCNA* and *CDK* family members (Humayun and Fornace, 2022). Inhibition of *dusp6* (a member of *dusp* phosphotase family) promotes CM proliferation in zebrafish (Han et al., 2014) and knockout of *Dusp6* attenuates cardiac damage after myocardial infarction in rats and mice (Maillet et al., 2008). *Dusp6* is transcriptionally activated by *p38*/*Cebpb* and also acts as a positive regulator to maintain p38 activity (Zhou et al., 2022). All these discoveries imply that the DHA/ppardb signaling may trigger a heart regeneration response.

Taken together, the results demonstrate that DHA promotes zebrafish heart regeneration depending on *ppardb*.

### *De novo* synthesis of DHA is required for heart regeneration of neonatal mice

**Figure 3.**
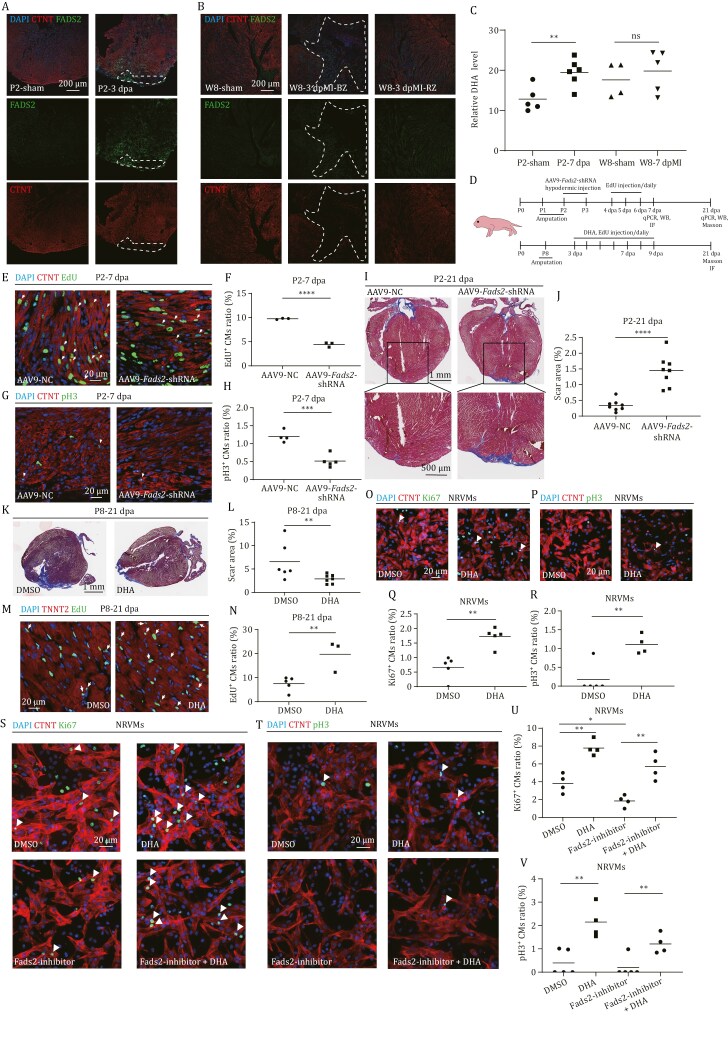
***De novo* synthesis of DHA is required for heart regeneration of neonatal mice.** (A and B) Immuno-staining of CTNT (in red) and FADS2 (in green) on cryosections of neonatal mouse (P2) injury hearts at sham and 3 dpa (A), and adult mouse (week 8, W8) injury hearts at sham and 3 dpMI (B). Red dotted lines indicate the approximate injury area. The representative picture was taken from 4–6 hearts in each group. Scale bar: 100 μm. (C) Relative DHA levels analyzed with LC-MS in neonatal mouse (P2) injury hearts at sham and 7 dpa, and adult mouse (W8) injury hearts at sham and 7 dpMI. Each treatment had 4–6 replicates. Each replicate had one injury heart. (D) Diagram showing the time schedule of the AAV9-*Fads2*-shRNA and the chemical injection in P2 and P8 mice. (E) Immuno-staining of CTNT (in red) and EdU incorporation assay (in green) of injury hearts of P2 hearts injected with AAV9-NC or AAV9-Fads2 shRNA at 7dpa. *n*: 3 hearts/sample. Scale bar: 20 μm. (F) Statistical analyses of EdU^+^ CMs in (E). The numbers of EdU^+^ CMs on heart sections were presented as the percentage of the total CTNT^+^ cells at the resection sites, respectively. Each dot represents an individual heart. (G) Immuno-staining of cTNT (in red) and pH3 (in green) of injury hearts of P2 hearts injected with AAV9-NC or AAV9-Fads2 shRNA at 7 dpa. *n*: 4–5 hearts/group; Scale bar: 20 μm. (H) Statistical analyses of pH3^+^ CMs in (G). (I) Fibrin clot stained with Masson’s trichrome on the cryosections of injury hearts of P2 neonatal mice injected with AAV9-NC or AAV9-Fads2 shRNA at 21 dpa. Framed areas in the upper panels were magnified in the lower panels. Scale bar: 1 mm or 500 μm in lower panels. (J) Statistical analyses of scar areas in (I). Average scar area with fibrin clots on heart sections was presented as the percentage of the total ventricular area. Each dot represents an individual heart. *n*: 8 hearts/group. (K) Fibrin clot stained with Masson’s trichrome on the cryosections of injury hearts of P8 mice injected with DMSO or DHA at 21 dpa. Scale bar: 1 mm. (L) Statistical analyses of scar areas in (K). Average scar area with fibrin clots on heart sections was presented as the percentage of the total ventricular area. Each dot represents an individual heart. *n*: 6–7 hearts/sample. (M) Immuno-staining of TNNT2 (in red) and EdU incorporation assay (in green) of injury hearts of P8 hearts injected with DMSO or DHA at 21 dpa. *n*: 3–5 hearts/sample. Scale bar: 20 μm. (N) Statistical analyses of EdU^+^ CMs in (M). The numbers of EdU^+^ CMs on heart sections were presented as the percentage of the total TNNT2^+^ cells at the resection sites, respectively. Each dot represents an individual heart. (O and P) Immuno-staining of CTNT (in red) and Ki67 (O) or pH3 (P) (in green) of NRVMs treated with DMSO or DHA for 24 h. Scale bar: 20 μm. (Q and R) Statistical analyses of Ki67^+^ CMs in (O) or pH3^+^ CMs in (P) were presented as the percentage of the Ki67^+^ or pH3^+^ of the total CMs. (S and T) Immuno-staining of CTNT (in red) and Ki67 (S) or pH3 (T) (in green) of NRVMs treated with DMSO or DHA or Fads2-inhibitor for 24 h. Scale bar: 20 μm. (U and V) Statistical analyses of Ki67^+^ CMs in (S) or pH3^+^ CMs in (T) were presented as the percentage of the Ki67^+^ or pH3^+^ of the total CMs. Statistical analysis was performed by Student’s two-tailed unpaired *t* test in GraphPad Prism 8. The *P* values were represented by ns and asterisks. ns, *P* > 0.05; **P < 0.01.

To investigate the role of DHA in mammalian heart regeneration, we performed heart surgery by amputating apex in P2 neonatal mice and induced myocardial infarction (MI) by ligation of the left anterior descending artery (LAD) in adult mice. Interestingly, *in situ* hybridization showed that the expression of two DHA synthesis genes (*Fads2* and *Elovl5*) was increased around injury site in neonatal injured hearts at 3 dpa (Fig. S7A). To verify if *Fads2* was induced in CMs, we performed co-immunofluorescence using FADS2 and CTNT (a CM marker) antibodies. Results showed that the expression of FADS2 was induced in both the injury area and the CMs near the injury area of neonatal hearts at 3 dpa, but was not obviously increased in CMs around the injury area of adult MI hearts, compared to the sham hearts (Fig. 3A and 3B). In addition to CMs, FADS2 protein was also increased in fibroblasts (α-SMA) and macrophages (F4/80) in neonatal injured hearts (Fig. S7B and S7C). Next, we conducted metabonomic analysis to compare DHA accumulation in the sham and injured hearts of both neonatal and adult mice at 7 dpi. The results showed that DHA accumulation was significantly increased only in the injured hearts of neonatal mice, but not of adult mice, compared to their respective sham hearts (Figs. 3C and S8A–D). *Fads2* and *Elovl5* are involved in the synthesis of both ω-3 and ω-6 poly-unsaturated fatty acids (PUFAs) (Fig. S8E). The upregulation of *Fads2* and *Elovl5* in injured hearts of neonatal mice also resulted in an increase of Arachidonic acid (AA) accumulation (Fig. S8G). However, other PUFAs were not significantly increased after injury in both neonatal and adult mice (Fig. S8F and S8G).

To evaluate the function of *de novo* DHA synthesis in heart regeneration, we blocked DHA synthesis by injecting Fads2 inhibitor (SC-26196) into injured hearts of neonatal mice from 3 to 9 dpa (Fig. S9E). The masson trichrome staining showed that the scar area at 21 dpa was significantly larger in Fads2 inhibitor-injected hearts than that in DMSO-injected hearts (Fig. S9F and S9G). To verify the function of *Fads2*, we knocked down *Fads2* in P2 neonatal mice by injecting AAV9-*Fads2*-shRNA at 1 dpa (Fig. 3D). qRT-PCR showed that *Fads2* mRNA level was significantly decreased in AAV9-*Fads2*-shRNA injected hearts at both 7 and 21 dpa (Fig. S9A and S9B). Western blot also confirmed the significant decrease of FADS2 protein in AAV9-*Fads2*-shRNA injected hearts at 21 dpa (Fig. S9C and S9D). Compared with the AAV9 negative control (AAV9-NC) injected hearts, the percentages of EdU^+^ and pH3^+^ CMs at 7 dpa were significantly decreased in AAV9-*Fads2*-shRNA injected hearts (Fig. 3E–H), whereas the masson trichrome staining showed that the scar area at 21 dpa was significantly increased in AAV9-*Fads2*-shRNA injected hearts (Fig. 3I and 3J). The results demonstrate that blocking DHA synthesis impairs heart regeneration in neonatal mice.

Next, we investigated whether DHA could prolong the heart regeneration window of mice. DHA was injected into P8 mice after apex resection from 3 dpa to 9 dpa (Fig. 3D). The masson trichrome staining showed that the scar area at 21 dpa was significantly decreased in DHA-injected hearts than that in DMSO-injected hearts (Fig. 3K and 3L). The percentages of EdU^+^ CMs at 21 dpa were also significantly increased in DHA-injected mice, compared to those in DMSO-injected controls (Fig. 3M and 3N).

Further, we isolated the neonatal (P2) rat primary ventricle myocytes (NRVMs) to evaluate whether DHA promoted CM proliferation *in vitro*. The results showed that the ratio of Ki67^+^ and pH3^+^ NRVMs was significantly increased in the DHA treatment (Fig. 3O–R). In contrast, the ratio of Ki67^+^ NRVMs was significantly reduced in the Fads2 inhibitor treatment, while Ki67^+^ and pH3^+^ NRVMs was restored by additional supplementation of DHA (Fig. 3S–V).

These data suggest that *de novo* synthesis of DHA promotes CM proliferation upon heart injury.

### Injection of DHA induces cell cycle progression in cardiomyocytes and prevents the decline of cardiac function in adult mice after MI

**Figure 4.**
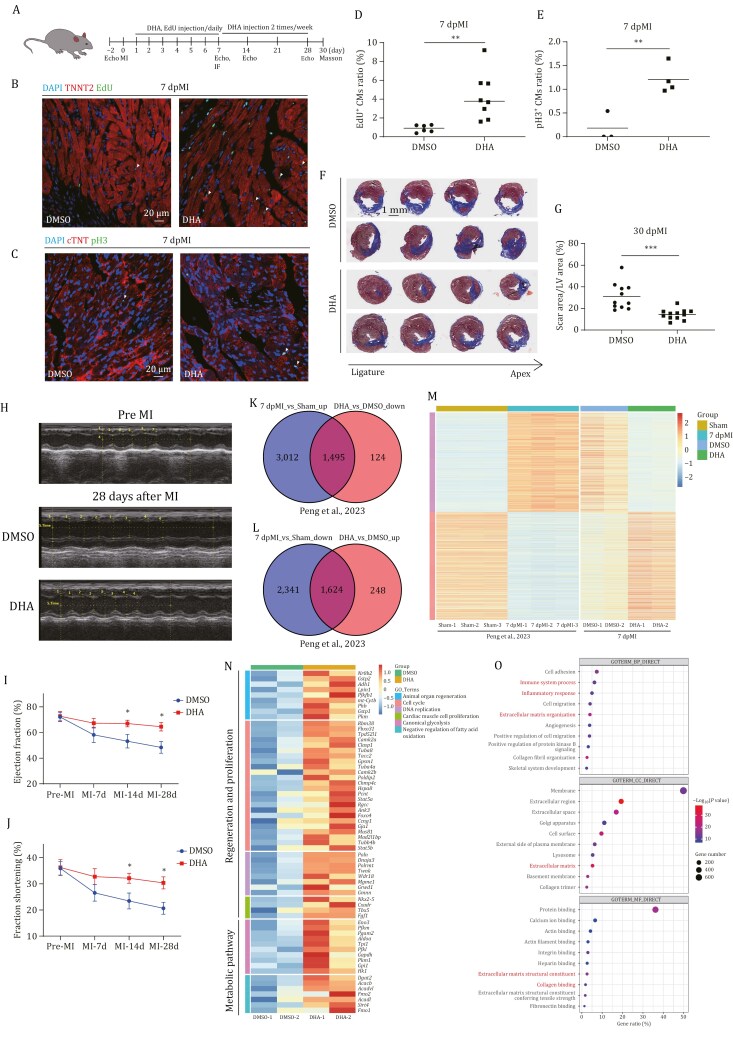
**DHA treatment induces CM proliferation and promotes heart repair to restore cardiac function in adult mice after MI**. (A) Diagram showing how the chemical injection was performed. The DHA and EdU were intraperitoneally injected into adult mice with MI treatment once daily for 7 days from 1 to 7 dpMI and twice a week from 8 to 30 dpMI. Echo: echocardiography; MI: myocardial infarction; IF: immune-staining; Masson: Masson staining. (B) Immuno-staining of TNNT2 (in red) and EdU incorporation assay (in green) of adult mouse hearts with MI treatment injected with DMSO or DHA at 7 dpMI. *n*: 6–8 hearts/sample. Scale bar: 20 μm. (C) Immuno-staining of cTNT (in red) and pH3 (in green) of adult mouse hearts with MI treatment injected with DMSO or DHA at 7 dpMI. *n*: 3–4 hearts/sample; Scale bar: 20 μm. (D and E)Statistical analyses of EdU^+^ CMs in (B) and pH3^+^ CMs in (C). The numbers of EdU^+^ CMs and pH3^+^ CMs on heart sections were presented as the percentage of the total TNNT2^+^ and cTNT^+^ cells at the resection sites, respectively. Each dot represents an individual heart. (F) Fibrin clot stained with Masson’s trichrome on the cryosections of injury hearts of adult mice injected with DMSO or DHA at 30 dpMI. Scale bar: 1 mm. (G) Statistical analyses of scar areas in (F). The average injury area with fibrin clots on heart sections was presented as the percentage of the left ventricular area. Each dot represents an individual heart. *n*: 11–12 hearts/sample. (H) Representative echocardiographic images of adult mice with MI treatment injected with DMSO or DHA at 28 dpMI. (I and J) Cardiac function indexes of the ejection fraction (EF) (I) and the fraction shortening (FS) (J) of adult mice with MI treatment injected with DMSO or DHA from echocardiography measurement on at −2 (before MI), 7, 14, 28 dpMI. (K) The Venn diagram shows that the upregulated genes in MI hearts compared to sham hearts overlapped with downregulated genes in DHA-treated MI hearts compared to DSMO-treated MI hearts at 7 dpMI. (L) The Venn diagram showing that the downregulated genes in MI hearts compared to sham hearts overlapped with upregulated genes in DHA-treated MI hearts compared to DSMO-treated MI hearts at 7 dpMI. (M) Heatmap of overlapped genes in (K) and (L). (N) Heatmaps of upregulated genes related to regeneration, proliferation, and metabolic pathways in DHA-treated MI hearts at 7 dpMI. (O) GO analysis of downregulated genes in DHA-treated MI hearts compared with DMSO-treated MI hearts at 7 dpMI. Statistical analysis was performed by Student’s two-tailed unpaired *t* test in GraphPad Prism 8. The *P* values were represented by ns and asterisks. ns, *P* > 0.05; *P < 0.05; **P < 0.01; ***P < 0.001.

Next, we intraperitoneally injected DHA into adult mice after MI, from 1 to 7 days post MI (dpMI) once a day, and from 8 to 28 dpMI twice a week (Fig. 4A). Cell proliferation examination showed that the percentages of EdU^+^ and pH3^+^ CMs at 7 dpMI were significantly increased in DHA-injected mice (4.31% and 1.21% respectively), compared to those in DMSO-injected controls (0.88% and 0.18%, respectively) (Fig. 4B–E), while the percentages of EdU^+^ CMs was not significantly changed in DHA-treated Sham hearts, compared to DMSO-treated controls (Fig. S10E and S10F). In contrast, immunohistochemistry showed that CD45 (a pan leukocyte marker) in the infarction area was obviously reduced by the DHA injection at 7 dpMI, suggesting that, similar to zebrafish, the immune response was down-regulated by DHA supplementation (Fig. S10A). Masson staining showed that the scar area at 28 dpMI was significantly reduced in the DHA-injected mice (14.4%), compared to that in the DMSO-injected controls (31.15%) (Fig. 4F and 4G). The results demonstrated that DHA supplementation also promoted heart repair post-injury in adult mice.

To assess if DHA supplementation restored cardiac function, we performed echocardiography. The results showed that the indexes of ejection fraction (EF) and fraction shortening (FS) at 7 dpMI were slightly increased, but not significantly, in the DHA-injected mice, compared to those in the DMSO-injected controls; however, the two indexes at 14 and 28 dpMI were significantly increased in the DHA-injected mice, compared to those in the DMSO-injected controls (Fig. 4H–J). There were no significant differences in the ratio of heart weight to body weight and the sizes of CMs between the DHA-injected mice and DMSO-injected mice (Fig. S10B–D), which indicated that DHA-injection would not cause obvious side effects, such as CM hypertrophy, during the experimental period.

The results demonstrate that DHA supplement not only promotes CM proliferation but also prevents the decline of cardiac function after MI.

### DHA supplementation promotes CM proliferation in adult MI hearts through PPARD

To further investigate the mechanism of DHA in promoting regeneration and CM proliferation, we isolated the 1/4 ventricles of one mouse heart at 7 dpMI with or without the DHA treatment to perform RNA-seq. We compared our data with the dataset of mouse sham hearts and 7 dpMI hearts published in 2023 (Yu et al., 2023). There were 1,872 genes upregulated, 1,619 genes down regulated in DHA injection compared with DMSO injection. Interestingly, among these DEGs, 1,624 upregulated genes were found to be downregulated, and 1495 downregulated genes were found to be upregulated in 7 dpMI hearts, compared to the sham hearts (Fig. 4K and 4L). The heatmap of these DEGs indicated that DHA injection almost altered the entire trancriptome of the injured hearts after MI (Fig. 4M). Among the upregulated genes in DHA-treated MI hearts, genes related to animal organ regeneration, cell cycle, DNA replication, and cardiac muscle cell proliferation were enriched, suggesting DHA induces a regeneration response (Fig. 4N). In addition, genes related glycolysis, negative regulation of fatty acid oxidation were also enriched in the upregulated genes in DHA-treated MI hearts (Fig. 4N), indicating the metabolic remodeling in proliferating CMs (Honkoop et al., 2019). On the other hand, GO analysis showed that immune response, ECM organization, and collage binding genes were significantly enriched in downregulated genes of DHA injection (Fig. 4O), which was consistent with the decrease of CD45 in the infarction area of the DHA-injected injury hearts at 7 dpMI (Fig. S10A). The results implied that DHA injection promoted heart repair in part by inhibiting immune response and reducing fibrosis.

In response to heart damage, zebrafish and neonatal mice can fully regenerate their hearts by directing the expression of a series of regeneration-response genes, which is known as the regeneration response. In contrast, adult mice have almost lost the capability of heart regeneration due to limited CM proliferation and activation of injury-dependent gene expression, such as the immune response and fibrosis-related genes, which is known as injury-response (Wang et al., 2020). Taken together, the RNA-seq analysis demonstrates that DHA supplementation may reshape MI hearts from injury response to regeneration response.

To investigate whether DHA, like in zebrafish, relied on PPARD to promote CM proliferation in mouse injury hearts, we intraperitoneally injected DHA or a PPARD inhibitor (GSK3787) (Magadum et al., 2017) or DHA along with the inhibitor into adult mice from 1 to 7 dpMI. In consistent with the above results, the injection of DHA alone significantly increased the percentage of EdU^+^ CMs in the injury areas of MI hearts, compared to DMSO-injected controls. However, the positive effects (the increase in CM proliferation) of DHA supplementation in MI heart repair were diminished by the co-injection of the PPARD inhibitor (Fig. S11A and S11B).

**Figure 5.**
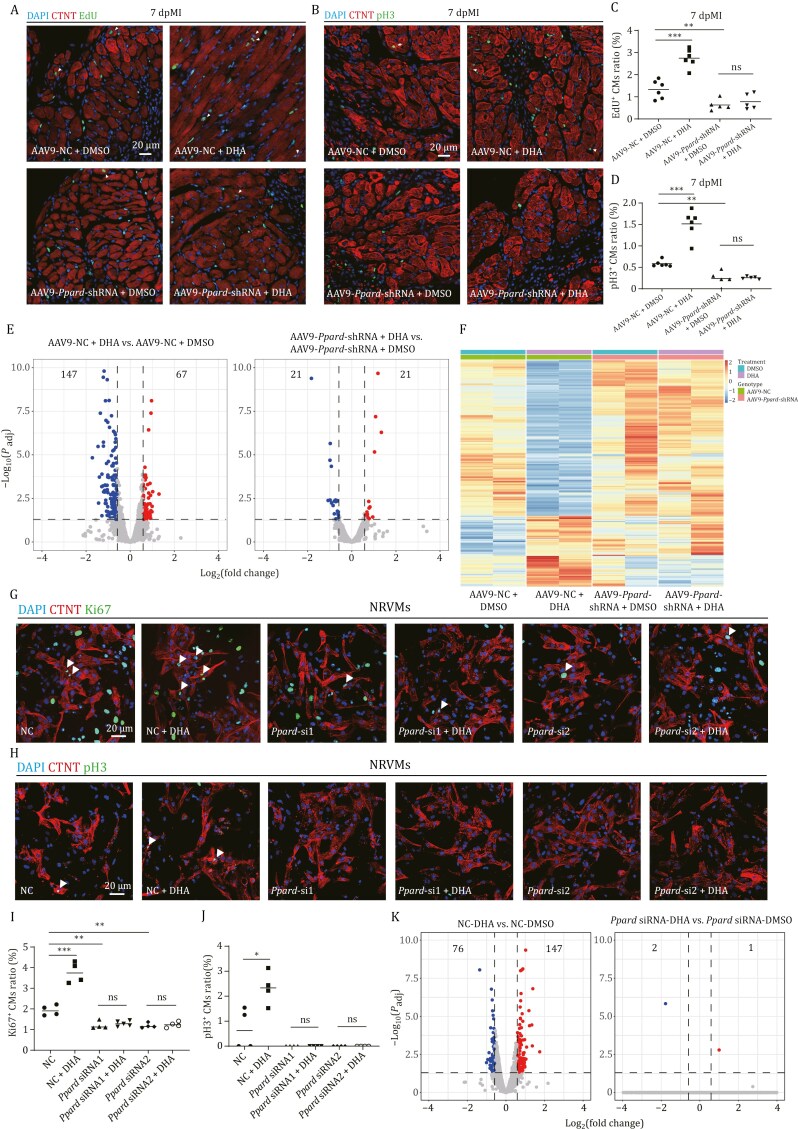
**DHA promotes CM proliferation through PPARD in adult mouse injury hearts and NRVMs.** (A) Immuno-staining of CTNT (in red) and EdU incorporation assay (in green) of adult mouse hearts with AAV9-NC or AAV9-*Ppard-*shRNA injected combined with DMSO or DHA injection at 7 dpMI. *n*: 5–6 hearts/sample. Scale bar: 20 μm. (B) Immuno-staining of cTNT (in red) and pH3 (in green) of adult mouse hearts with AAV9-NC or AAV9-*Ppard-*shRNA injected combined with DMSO or DHA injection at 7 dpMI. *n*: 5–6 hearts/sample; Scale bar: 20 μm. (C and D) Statistical analyses of EdU^+^ CMs in (A) or pH3^+^ CMs (B). The numbers of EdU^+^ or pH3^+^ CMs on heart sections were presented as the percentage of the total CTNT^+^ cells at the resection sites, respectively. Each dot represents an individual heart. (E) Volcano plots showing the DEGs (|fold change| ≥ 1.5, *P*_adj_ < 0.05) of injury hearts at 7 dpMI in two comparisons: AAV9-NC-DHA versus AAV9-NC-DMSO, and AAV9-*Ppard-*shRNA-DHA versus AAV9-*Ppard-*shRNA-DMSO. (F) Heatmap of DEGs in AAV9-NC-DHA compared to AAV9-NC-DMSO. (G and H) Immuno-staining of CTNT (in red) and Ki67 (G) or pH3 (H) (in green) of NRVMs transfected with negative control (NC), *Ppard* siRNA for 24 h, and treated with DMSO or DHA for 24 h. Scale bar: 20 μm. (I and J) Statistical analyses of Ki67^+^ CMs in (G) or pH3^+^ CMs in (H) were presented as the percentage of the Ki67^+^ or pH3^+^ of the total CMs. (K) Volcano plots showing the DEGs (|fold change)| ≥ 1.5, *P*_adj_ < 0.05) of NRVMs in two comparisons: NC-DHA versus NC-DMSO, and *Ppard* siRNA-DHA versus *Ppard* siRNA-DMSO. Statistical analysis was performed by Student’s two-tailed unpaired *t* test in GraphPad Prism 8. The *P* values were represented by ns and asterisks. ns, *P* > 0.05; *P < 0.05; **P < 0.01; ***P < 0.001.

To verify the function of *Ppard*, we knocked down *Ppard* by injecting AAV9-*Ppard*-shRNA in the adult mice 14 days before MI (Fig. S11G). Subsequently, DHA or DMSO was injected into AAV9-*Ppard*-shRNA and AAV9-NC injected mice at 2–6 dpMI. Western blot showed that PPARD protein was significantly reduced in the AAV9-*Ppard*-shRNA injected hearts at 7 dpMI (Fig. S11H and S11I). Similar to the results in DHA-treated WT MI hearts (Fig. 4B–E), the percentages of EdU^+^ and pH3^+^ CMs were also significantly increased by DHA treatment at 7 dpMI in AAV9-NC-injected hearts, compared to DSMO-treated AAV9-NC-injected hearts. In contrast, the percentages of EdU^+^ and pH3^+^ CMs were significantly decreased at 7 dpMI in DMSO-treated AAV9-*Ppard*-shRNA injected hearts, compared to DSMO-treated AAV9-NC injected hearts, and the significant increase by DHA treatment was completely abolished by knockdown of *Ppard* in DHA-treated AAV9-*Ppard*-shRNA injected hearts (Figs. 5A–D, S11K and S11L).

To investigate how the DHA/PPARD axis promotes heart regeneration in adult mice, we performed RNA-seq to compare the transcriptomes of DHA-treated injured hearts with DMSO-treated control hearts at 7 dpMI in the context of *Ppard* knockdown with AAV9-*Ppard*-shRNA. DEG analysis (|log_2_(fold change)| ≥ 0.68, *P*_adj_ < 0.05) identified that 147 genes were downregulated and 67 genes were upregulated by DHA treatment in AAV9-NC injected injury hearts, whereas only 21 genes were downregulated and 21 genes were upregulated by DHA treatment in the AAV9-*Ppard*-shRNA injected injury hearts (Fig. 5E). The heatmap of these DEGs also showed that most DEGs in DHA-treated AAV9-NC injected injury hearts did not exhibit similar trends in DHA-treated AAV9-*Ppard*-shRNA injected injury hearts, suggesting that DHA altered gene expression in adult injury hearts through PPARD (Fig. 5F). Among the DEGs, 11 negative regulators of cell proliferation were enriched in the downregulated genes by DHA treatment in AAV9-NC injected injury hearts, which did not appear in the downregulated genes by DHA treatment in AAV9-*Ppard*-shRNA injury hearts (Fig. S11J), consistent with the results in CM proliferation assay (Figs. 5A–D, S11K and S11L).

Next, we used *in vitro* culture of NRVMs to address whether DHA directly promotes CM proliferation via PPARD. *Ppard* expression was knocked down by two *Ppard* siRNAs in NRVMs treated with DHA or DMSO (Fig. S11M). Consistent with *in vivo* results, the proportions of Ki67^+^ and pH3^+^ NRVMs were significantly increased by DHA supplementation, while the increase was completely eliminated by two *Ppard* siRNAs (Fig. 5G–J). In addition, the proportion of proliferating NRVMs was also significantly decreased by *Ppard* knockdown.

RNA-seq was employed to compare the transcriptome of NRVMs treated with DHA and DMSO in the context of *Ppard* knockdown. DEG analysis identified that 76 genes were downregulated and 147 genes were upregulated by DHA treatment in NC-siRNA-transfected NRVMs, whereas only 2 genes were downregulated and 1 gene was upregulated by DHA treatment in the *Ppard* siRNA-transfected NRVMs (Fig. 5K), which were similar to the RNA-seq data in zebrafish and adult mice injured hearts. GO analysis on the DEGs showed that several pathways related to cell proliferation, such as PI3K-AKT and ERK, were enriched in the upregulated genes by DHA treatment in NC-siRNA-transfected NRVMs, consistent with the function of DHA in the proliferation of NRVMs (Fig. S11N).

The results of two *Ppard* siRNAs in NRVMs were verified by a PPARD inhibitor GSK3787. GSK3787 also impaired the increase of Ki67^+^ or pH3^+^ NRVMs in the DHA treatment (Fig. S11C–F).

Taken together, DHA supplementation in adult mice or NRVMs promotes heart repair and CM proliferation via PPARD.

### DHA supplementation inhibits inflammation and fibrosis in adult MI hearts through PPARD

Interestingly, genes related to extracellular matrix (ECM) and immune response were also downregulated by DHA treatment in AAV9-NC-injected injury hearts, which was abolished by *Ppard* knockdown (Fig. S12A). Macrophage polarization from M1 (pro-inflammatory) to M2 (anti-inflammatory) plays a crucial role in immune response regulation. As the induction of *Fads2* expression was also detected in macrophages, immunohistochemistry was performed to examine CD68 (M1 macrophage marker) and ARG1 (M2 macrophage marker) in DHA or DMSO-treated AAV9-NC and AAV9-*Ppard*-shRNA-injected injury hearts. The results showed that DHA treatment significantly decreased the CD68 level and increased the ARG1 level in the border zones of AAV9-NC MI hearts but not in AAV9-*Ppard*-shRNA MI hearts (Fig. S12B–E). These findings suggest that DHA can also rely on PPARD to inhibit inflammation in adult injury hearts, possibly through macrophage polarization.

To assess whether DHA has an effect on cardiac fibroblasts, we isolated neonatal rat primary cardiac fibroblasts (NRCFs). Similar to the above results, supplementation of DHA (from 0.5 μmol/L to 5 μmol/L, except 10 μmol/L) significantly promoted NRVM proliferation. On the contrary, the proportion of EdU^+^ NRCFs gradually decreased significantly, as the DHA concentration increased from 1 μmol/L to 10 μmol/L (Fig. S13A–D). Wound closure assay also showed that the migration rate of NRCFs gradually decreased significantly, as the DHA concentration increased from 2 μmol/L to 10 μmol/L (Fig. S13E and S13F). We then combined DHA treatment with the PPARD inhibitor GSK3787 to test whether DHA relied on PPARD to inhibit NRCF proliferation and migration. The results showed that upon the inhibition of PPARD, DHA treatment no longer inhibited NRCF proliferation and migration (Fig. S14A–D), indicating that DHA inhibits NRCF proliferation and migration through PPARD.

Thus, the data suggest that DHA may inhibit inflammation and fibrosis through PPARD.

### DHA activates PPARD to bind to the promoter regions of heart regeneration-related genes

**Figure 6.**
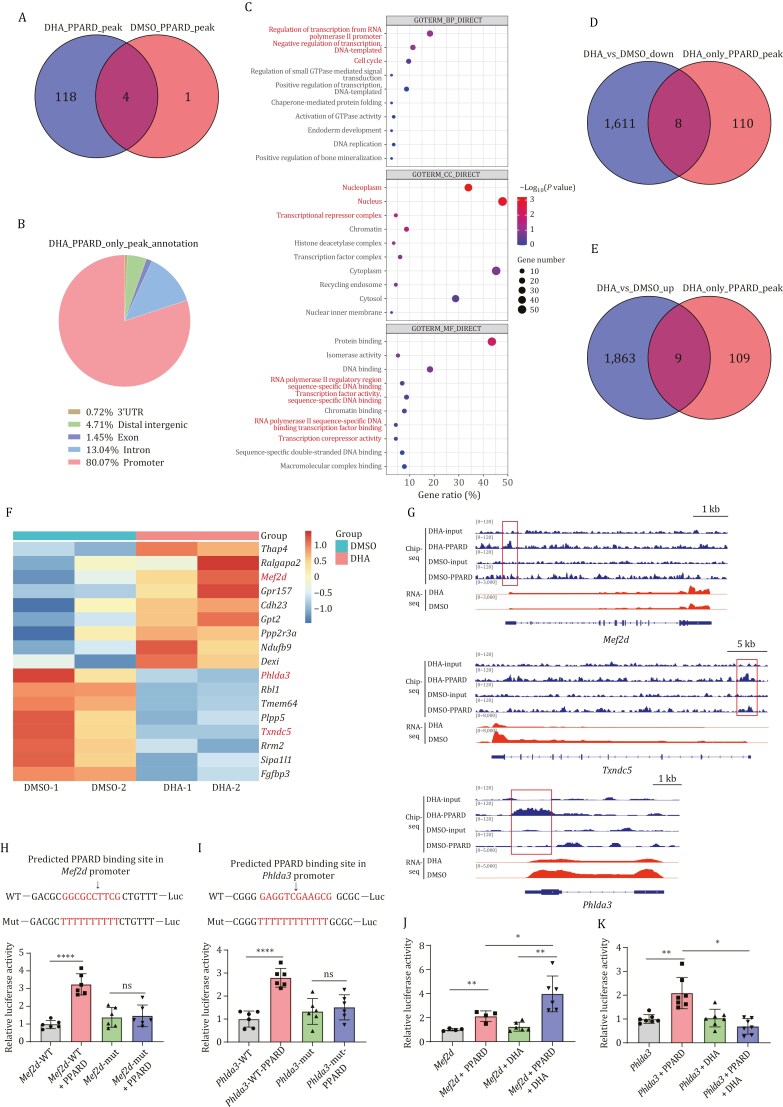
**DHA promotes CM proliferation by activating PPARD to bind to the promoter regions of heart regeneration-related genes to regulate their expression**. (A) The Venn diagram showing the overlapped genes between genes with PPARD peaks in DHA-treated MI hearts and DMSO-treated MI hearts at 7 dpMI. (B) The location distribution of PPARD peaks unique to DHA treated MI hearts at 7 dpMI. (C) GO analysis of genes with PPARD peaks unique to DHA treated MI hearts at 7 dpMI. (D and E) The Venn diagram showing the overlapped genes with PPARD peaks unique to DHA-treated MI hearts and downregulated genes in DHA-treated MI hearts against to DMSO-treated MI hearts at 7 dpMI (D) or upregulated genes in DHA-treated MI hearts against to DMSO-treated MI hearts at 7 dpMI (E). (F) The heat map of the overlapped genes in (D and E). (G) IGV tracks showing PPARD and RNA peaks at *Phlda3*, *Txndc5,* and *Mef2d* loci in DHA-treated MI hearts and DMSO-treated MI hearts at 7 dpMI. (H–K) Luciferase assays for the transcriptional activities of WT- and mut-*Mef2d* or *Phlda3* promoters in 293T cells. The predicted PPARD binding motif (in red) in *Mef2d* (H) or *Phlda3* (I) WT-promoter was replaced with the same number of oligo-T (in red) to generate *Mef2d*- or *Phlda3-*mut promoter. Luciferase assay system was used to analyze the transcriptional activities of these promoters in the context of *Ppard* expression (H and I), or *Ppard* expression combined with DHA supplementation (J and K) as indicated. Statistical analysis was performed by Student’s two-tailed unpaired *t* test in GraphPad Prism 8. The *P* values were represented by ns and asterisks. ns, *P* > 0.05. *P < 0.05; **P < 0.01; ***P < 0.001.

To identify genes directly regulated by PPARD in DHA-treated injury hearts, we conducted ChIP-seq at 7dpMI using a PPARD antibody. Only 5 peaks were enriched by the PPARD antibody in DMSO-injected injury hearts, whereas there were 122 peaks enriched in DHA-injected injury hearts (Fig. 6A), suggesting that PPARD transcription activity was triggered by DHA. Subsequently, we focused on peaks unique to DHA treatment and observed that these peaks were predominantly enriched in promoter regions (Fig. 6B). GO analysis of the genes with peaks unique to the DHA treatment revealed that they were enriched in transcription, transcriptional repression, and cell cycle regulation (Fig. 6C). Among 117 genes with the PPARD peaks in DHA injected injury hearts, RNA-seq analysis showed that 8 genes were upregulated and 9 genes were downregulated by DHA treatment (Fig. 6D–G). Among these 17 genes, *Mef2d*, *Phlda3*, and *Txndc5* have been reported to play roles in CM proliferation, fibrosis formation, or heart repair processes, respectively (Meng et al., 2021; Shih et al., 2018; Wang et al., 2023).

Next, we chose *Mef2d* representing upregulated genes and *Phlda3* representing downregulated genes (both are not known as the target genes of PPARD) to validate the DHA-mediated PPARD transcriptional activity. About 1.5–2 kb DNA fragment containing PPARD peaks around *Med2d* or *Phlda3* transcription start site was cloned as *Med2d* or *Phlda3* WT promoter, respectively. The predicted top-scoring binding motif of PPARD in the promoter of *Med2d* or *Phlda3* was substituted with oligo-T to generate the corresponding mutant promoter: *Med2d*-mut-P or *Phlda3*-mut-P (Fig. 6H and 6I). The luciferase reporter driven by these promoters was used to analyze transcriptional activity in 293T cells. The luciferase assays showed that PPARD significantly enhanced the transcriptional activity of both *Mef2d* and *Phlda3* WT promoter, but had little effect on their mutant promoters (Fig. 6H and 6I). Interestingly, DHA significantly increased transcriptional activity on *Mef2d* WT promoters, but significantly repressed transcriptional activity on *Phlda3* WT promoters, which was consistent with the transcriptomic analysis of DHA-treated MI hearts (Fig. 6J and 6K).

All these results demonstrate that DHA-activated PPARD can upregulate or downregulate the expression of certain target genes by directly binding to their promoters.

### DHA exhibits a unique binding pose with PPARD to regulate the transcription of downstream genes

**Figure 7.**
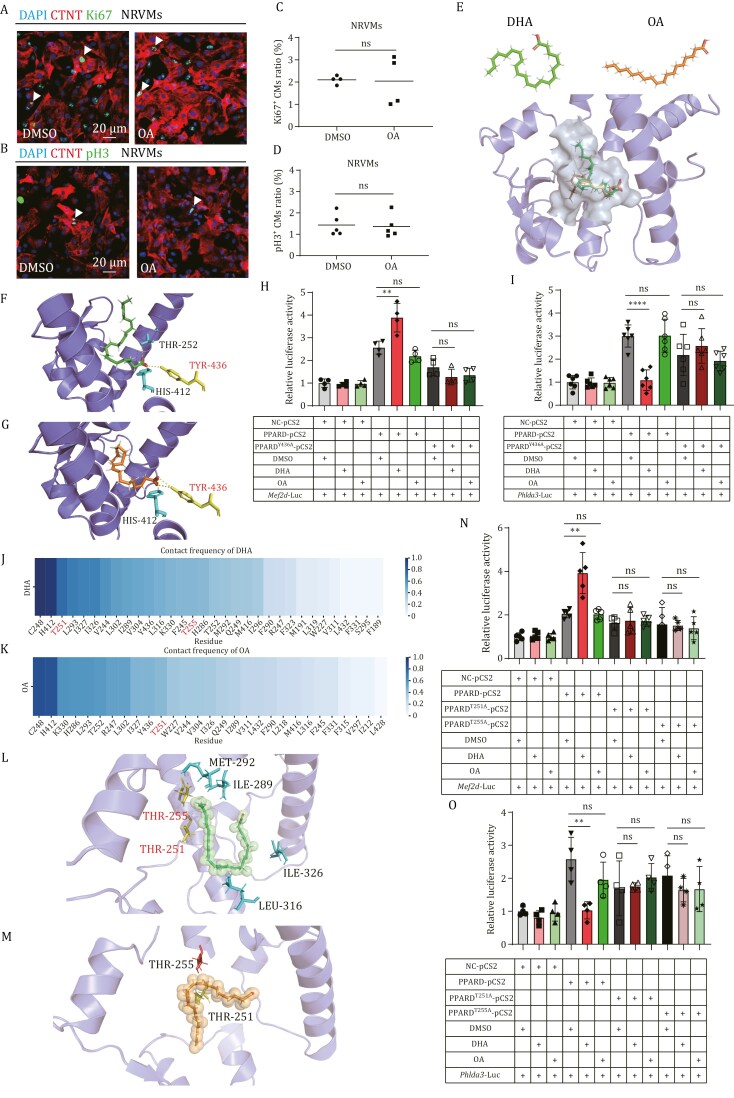
**DHA, rather than OA, triggers PPARD transcriptional activity for heart regeneration due to its unique binding pose with PparD.** (A and B) Immuno-staining of CTNT (in red) and Ki67 (A) or pH3 (B) (in green) of NRVMs treated with DMSO or OA for 24 h. Scale bar: 20 μm. (C and D) Statistical analyses of Ki67^+^ CMs in (A) or pH3^+^ CMs in (B) were presented as the percentage of the Ki67^+^ or pH3^+^ of the total CMs. (E) Structure scheme of DHA (green), OA (orange) and predicted binding pocket (gray) of DHA and OA with PPARD (purple) structure by Autodock. (F and G) Details of the interaction between the head of DHA (F) or OA (G) and different residues in the binding pocket (purple) of PPARD. The dash line represented hydrogen-bond. The residue TYR-436 in red was selected to verify the interaction. (H and I) Luciferase assays for the transcriptional activities of WT-PPARD and PPARD^Y436A^ mutant in 293T cells. The reside TYR-436 in PPARD binding pocket predicted for the interaction with the head of DHA or OA was mutated into Ala to generated PPARD^Y436A^ mutant. *Mef2d* (H) or *Phlda3* (I) promoter was used to analyze the transcriptional activity of PPARD^Y436A^ mutant in the context of DHA or OA supplementation as indicated. (J and K) The contact frequencies of different PPARD residues with DHA (J) or OA (K) analyzed by molecular dynamic simulation. The residues in red were selected to verify the interaction. (L and M) Details of the interaction between DHA (L) or OA (M) and different residues in the binding pocket (purple) of PPARD by molecular dynamic simulation. (N and O) Luciferase assays for the transcriptional activities of WT-PPARD, PPARD^T251A^, and PPARD^T255A^ mutants in 293T cells. The residue THR-251 or THR-255 in PPARD binding pocket was mutated into Ala to generate PPARD^T251A^ or PPARD^T255A^ mutant. *Mef2d* (N) or *Phlda3* (O) promoter was used to analyze the transcriptional activity of PPARD^T251A^ or PPARD^T255A^ mutant in the context of DHA or OA supplementation as indicated. Statistical analysis was performed by Student’s two-tailed unpaired *t* test in GraphPad Prism 8. The *P* values were represented by ns and asterisks. ns, *P* > 0.05; *P < 0.05; **P < 0.01; ***P < 0.001.

PPARD is a well-known nuclear receptor and binds to the promoters of its target genes to regulate their expression when it forms a complex with its ligand, such as small hydrophobic molecules. It has been demonstrated that ω-3 PUFAs, including DHA and EPA, can bind to PPARβ/δ to function as an agonist (Korbecki et al., 2019). In addition to ω-3 PUFAs, other unsaturated fatty acids, such as oleic acid (OA)-an monounsaturated fatty acid, can also bind to PPARD to regulate the expression of its target genes (Grygiel-Gorniak, 2014), which raises a question of whether OA has similar functions in promoting CM proliferation to DHA. However, unlike DHA, OA did not increase the ratio of Ki67^+^ or pH3^+^ NRVMs (Fig. 7A–D).

To investigate the molecular basis of this difference, we used AlphaFold3 to predict the structure of PPARD and performed molecular docking to examine how DHA and OA interact with its ligand-binding domain (Fig. 7E). While DHA and OA showed similar head group positioning within the PPARD binding pocket, their tail orientations diverged (Fig. 7F and 7G). Both fatty acids formed hydrogen bonds with residues HIS-412 and TYR-436 in the head region, involving the hydroxyl group of TYR-436 and the amine group of HIS-412 interacting with the carboxyl groups of the fatty acids. To experimentally validate the predictions, we mutated the residue TYR-436 of PPARD into ALA (PPARD^Y436A^), which was expected to disrupt the hydrogen bond interaction with the ligands. We then analyzed the PPARD^Y436A^ transcriptional activity in the context of DHA and OA using 293T cells with the *Mef2d* and *Phlda3* promoters. The results showed that the transcriptional activity of both *Mef2d* and *Phlda3* promoters was significantly increased by PPARD-WT or PPARD^Y436A^ mutant. However, DHA was observed to promote the transcriptional activity of *Mef2d* promoter and repress the transcriptional activity of *Phlda3* promoter only in PPARD-WT, but not in PPARD^Y436A^-mutant, while OA displayed a little effect on the transcriptional activity of both PPARD-WT and PPARD^Y436A^ mutant (Fig. 7H and 7I).

To further validate and explore the dynamics of the binding, we conducted molecular dynamics (MD) simulations of the DHA/OA-PPARD complex. The simulations showed that while the head groups of DHA and OA remained stable in the binding pocket, their tails exhibited distinct dynamic behaviors and interactions (Videos S1 and S2). Contact frequency analysis revealed different interaction modes for DHA and OA. These differences in ligand interactions likely underlie the functional divergence between DHA and OA. Among the analyzed residues, T251 exhibited a high contact frequency with DHA and a moderate frequency with OA, while T255 showed a moderate contact frequency with DHA and no contact with OA (Fig. 7J–M), suggesting these two threonines might play a role in interpreting the function of DHA. We further examined the ligand tail binding pocket and introduced mutations at T251 and T255 (T251A and T255A). These mutations reduced DHA’s ability to enhance *Mef2d* transcription and suppress *Phlda3* transcription (Fig. 7N and 7O). This is consistent with the MD simulations of the PPARD complexes, which showed that both T251 and T255 interact more frequently with DHA than with OA.

Collectively, compared to OA, DHA has a distinct binding pose with PPARD, which may lead to unique conformation changes in PPARD, thereby activating PPARD to bind to the promoters of its target genes related to heart regeneration and regulate their expression.

### DHA promotes the proliferation of CMs via activating the transcription of *Mef2d* as well as inhibiting the transcription of *Phlda3* and *Txndc5*

**Figure 8.**
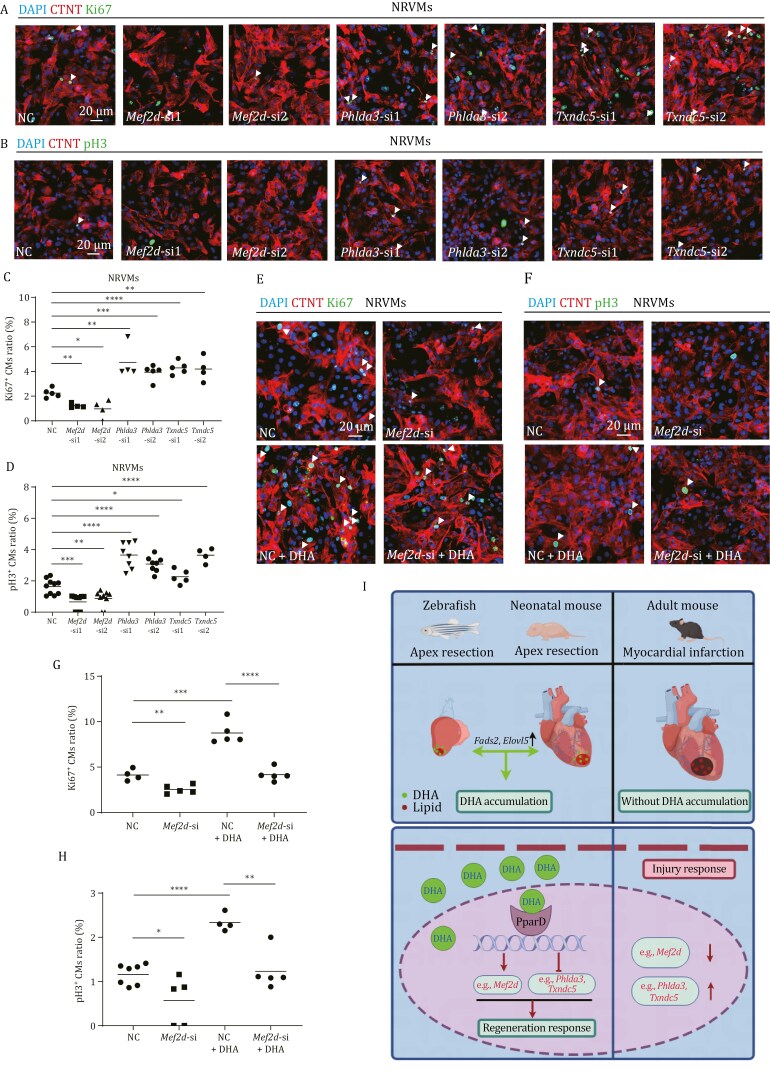
**DHA promotes the proliferation of CMs via activating the transcription of *Mef2d* as well as inhibiting the transcription of *Phlda3* and *Txndc5*.** (A and B) Immuno-staining of CTNT (in red) and Ki67 (A) or pH3 (B) (in green) of NRVMs transfected with negative control (NC), *Mef2d*, *Phlda3,* or *Txndc5* siRNA for 24 h. Scale bar: 20 μm. (C and D) Statistical analyses of Ki67^+^ CMs in (A) or pH3^+^ CMs in (B) were presented as the percentage of the Ki67^+^ or pH3^+^ of the total CMs. (E and F) Immuno-staining of CTNT (in red) and Ki67 (E) or pH3 (F) (in green) of NRVMs transfected with *Mef2d* siRNA or NC siRNA and treated with DHA or DMSO for 24 h. Scale bar: 20 μm. (G and H) Statistical analyses of Ki67^+^ CMs in (E) or pH3^+^ CMs in (F) were presented as the percentage of the Ki67^+^ or pH3^+^ of the total CMs. (I) Model of DHA function in heart regeneration. The diagram is drawn in Figdraw 2.0. Statistical analysis was performed by Student’s two-tailed unpaired *t* test in GraphPad Prism 8. The *P* values were represented by ns and asterisks. ns, *P* > 0.05; *P < 0.05; **P < 0.01; ***P < 0.001.

Finally, we used NRVMs to investigate the function of DHA/PPARD axis in promoting CM proliferation in the context of its target genes. qRT-PCR showed that *Mef2d*, *Phlda3,* or *Txndc5* was effectively knocked down by its two gene-specific siRNAs, respectively (Fig. S15A and S15B). As expected, knockdown of *Mef2d* significantly decreased the ratio of Ki67^+^ and pH3^+^ NRVMs, whereas knockdown of *Phlda3* or *Txndc5* significantly increased the ratio of Ki67^+^ and pH3^+^ NRVMs (Fig. 8A–D). Furthermore, the increase of Ki67^+^ and pH3^+^ NRVMs in DHA supplementation was abolished by knockdown of *Mef2d* (Fig. 8E–H).

Taken together, DHA/PPARD axis promotes CM proliferation through activating heart regeneration responses, such as promoting the transcription of *Mef2d* and inhibiting the transcription of *Phlda3* and *Txndc5*.

## Discussion

In summary, we have revealed that DHA accumulation from *de novo* synthesis functions as a signal to promote heart regeneration response. In brief, cardiac injury can induce a metabolic switch. The expression of DHA synthesis genes is activated only in zebrafish and neonatal mice, but not in adult mice, which results in DHA accumulation around injury sites. *De novo* synthesized DHA triggers heart regeneration response by activating *Ppard*, which promotes CM proliferation through enhancing the expression of heart regeneration positive regulators such as *Mef2d*, and repressing the expression of negative regulators such as *Phlda3* and *Txndc5* (Fig. 8I).

It is found that knockout of *Cpt1b* in mice results in accumulation of α-ketoglutarate, which activates KDM5 to demethylate broad H3K4me3 domains in genes for CM maturation, and finally stimulates CM proliferation, allowing heart regeneration after ischemia–reperfusion injury of adult mice (Li et al., 2023). Here, we found that the expression of *cpt1ab* responsible for fatty acid transport from cytoplasm to mitochondria was decreased in the proliferating CMs, accompanied by lipid accumulation around the injury site of zebrafish heart. Similar to the *Cpt1b* knockout in mice (Li et al., 2023), Cpt1 depletion also promoted zebrafish heart regeneration, and resulted in more lipid accumulation around the injury site. The results suggested that lipid accumulation might be required for zebrafish heart regeneration.

Lipid utilization has conventionally been considered as a simple detrimental factor in mammalian heart regeneration due to lipotoxicity. However, just like every coin has two sides, there are also “beneficial lipids” that promote the heart regeneration. Sphingosine-1-phosphate (S1P), a central component of sphingolipids, has been found to play a role in mammalian heart regeneration (Ji et al., 2024). In this report, we have demonstrated that DHA is also a “beneficial lipid” for heart regeneration.

The major challenge for the heart regeneration is how to stimulate polyploid CMs to undergo cytokinesis or endoreplication to compensate for the loss of cardiac mass in adult mammals. However, excessive induction of CM proliferation in adult mice may lead to heart tumor formation (Chen et al., 2021). In our studies, DHA injection didn’t obviously promote CM proliferation under normal condition, suggesting that DHA treatment would not lead to oncogenesis in hearts.

Clinical studies show that higher intakes of DHA and EPA are associated with lower risk of developing cardiovascular disease (1999). This may be because of the multiple molecular and cellular functions of EPA and DHA, such as their antioxidant and anti-inflammatory roles, regulation of platelet homeostasis, and lower risk of thrombosis (Oppedisano et al., 2020). However, some clinical studies have also showed that the effects of EPA and DHA in the treatment of MI patients are not consistent (Ajith and Jayakumar, 2019). One possible reason for this phenomenon may be the delivery efficiency of EPA/DHA to the heart, as EPA/DHA is orally taken by patients. Here, we intraperitoneally injected DHA into the animals, which might increase the delivery efficiency. Additionally, we also found that the concentration of DHA is crucial for NRVMs as the cells lost adhesion ability and viability when the concentration of DHA reached 10 µmol/L. Therefore, the precise dosage of DHA and the method of delivery are of prime importance to the future clinical studies. However, some indirect effects of the DHA injection may also result from different feedback pathways or the coordinated delivery to multiple organs. It is still uncertain how the injected DHA reaches the injured heart to promote CM proliferation.

It has been demonstrated that, as a nuclear receptor, PPARs can bind to various ligands to activate downstream signaling pathways. However, whether or how different ligands trigger different functions of PPARs remains elusive. In this study, we revealed that DHA, but not OA, activated PPARD to bind to its specific target genes, thereby triggering a heart regeneration response.

Taken together, we have demonstrated that the activation of the DHA/PPARD signaling pathway plays a key role in heart regeneration response, which may be useful for treating heart failure diseases.

## Supplementary data

Supplementary data are available at *Protein & Cell* online https://doi.org/10.1093/procel/pwaf062.

pwaf062_Supplementary_Materials

## Data Availability

All relevant data are available from the authors and/or included in the manuscript or Supplementary information. Sequencing data in this paper have been deposited in the NCBI database (BioProject: PRJNA938172 (zebrafish), PRJNA1172169 (mouse), PRJNA1251510 (rat)). The resource data of LC-MS will be available, if they are required.

## References

[CIT0003] Ajith TA, Jayakumar TG. Omega-3 fatty acids in coronary heart disease: recent updates and future perspectives. Clin Exp Pharmacol Physiol 2019;46:11–18. 30230571 10.1111/1440-1681.13034

[CIT0004] Cardoso AC, Lam NT, Savla JJ et al Mitochondrial substrate utilization regulates cardiomyocyte cell cycle progression. Nat Metab 2020;2:167–178. 32617517 PMC7331943

[CIT0005] Chablais F, Jazwinska A. The regenerative capacity of the zebrafish heart is dependent on TGFbeta signaling. Development 2012;139:1921–1930. 22513374 10.1242/dev.078543

[CIT0006] Chen J, Ng SM, Chang C et al p53 isoform delta113p53 is a p53 target gene that antagonizes p53 apoptotic activity via BclxL activation in zebrafish. Genes Dev 2009;23:278–290. 19204115 10.1101/gad.1761609 PMC2648546

[CIT0007] Chen Y, Luttmann FF, Schoger E et al Reversible reprogramming of cardiomyocytes to a fetal state drives heart regeneration in mice. Science 2021;373:1537–1540. 34554778 10.1126/science.abg5159

[CIT0008] Cheng X, Ju J, Huang W et al cpt1b regulates cardiomyocyte proliferation through modulation of glutamine synthetase in zebrafish. J Cardiovasc Dev Dis 2024;11:344. 39590187 10.3390/jcdd11110344 PMC11594654

[CIT0011] D’Souza K, Nzirorera C, Kienesberger PC. Lipid metabolism and signaling in cardiac lipotoxicity. Biochim Biophys Acta 2016;1861:1513–1524. 26924249 10.1016/j.bbalip.2016.02.016

[CIT0009] Dai J, Liang K, Zhao S et al Chemoproteomics reveals baicalin activates hepatic CPT1 to ameliorate diet-induced obesity and hepatic steatosis. Proc Natl Acad Sci USA 2018;115:E5896–E5905. 29891721 10.1073/pnas.1801745115 PMC6042128

[CIT0001] Dietary supplementation with n-3 polyunsaturated fatty acids and vitamin E after myocardial infarction: results of the GISSI-Prevenzione trial. Gruppo Italiano per lo Studio della Sopravvivenza nell’Infarto miocardico. Lancet 1999;354:447–455. 10465168

[CIT0010] Doenst T, Nguyen TD, Abel ED. Cardiac metabolism in heart failure: implications beyond ATP production. Circ Res 2013;113:709–724. 23989714 10.1161/CIRCRESAHA.113.300376 PMC3896379

[CIT0013] Engel FB, Schebesta M, Duong MT et al p38 MAP kinase inhibition enables proliferation of adult mammalian cardiomyocytes. Genes Dev 2005;19:1175–1187. 15870258 10.1101/gad.1306705 PMC1132004

[CIT0014] FitzSimons M, Beauchemin M, Smith AM et al Cardiac injury modulates critical components of prostaglandin E_2_ signaling during zebrafish heart regeneration. Sci Rep 2020;10:3095. 32080283 10.1038/s41598-020-59868-6 PMC7033201

[CIT0015] Flinn MA, Jeffery BE, O’Meara CC et al Yap is required for scar formation but not myocyte proliferation during heart regeneration in zebrafish. Cardiovasc Res 2019;115:570–577. 30295714 10.1093/cvr/cvy243

[CIT0016] Fukuda R, Marin-Juez R, El-Sammak H et al Stimulation of glycolysis promotes cardiomyocyte proliferation after injury in adult zebrafish. EMBO Rep 2020;21:e49752. 32648304 10.15252/embr.201949752 PMC7403660

[CIT0017] Gemberling M, Karra R, Dickson AL et al Nrg1 is an injury-induced cardiomyocyte mitogen for the endogenous heart regeneration program in zebrafish. ELIFE 2015;4:e05871. 25830562 10.7554/eLife.05871 PMC4379493

[CIT0019] Gonzalez-Rosa JM, Sharpe M, Field D et al Myocardial polyploidization creates a barrier to heart regeneration in zebrafish. Dev Cell 2018;44:433–446.e7. 29486195 10.1016/j.devcel.2018.01.021 PMC5830170

[CIT0020] Grygiel-Górniak B. Peroxisome proliferator-activated receptors and their ligands: nutritional and clinical implications – a review. Nutr J 2014;13:17. 24524207 10.1186/1475-2891-13-17 PMC3943808

[CIT0022] Han P, Zhou XH, Chang N et al Hydrogen peroxide primes heart regeneration with a derepression mechanism. Cell Res 2014;24:1091–1107. 25124925 10.1038/cr.2014.108 PMC4152734

[CIT0021] Han L, Shen WJ, Bittner S et al PPARs: regulators of metabolism and as therapeutic targets in cardiovascular disease. Part II: PPAR-β/δ and PPAR-γ. Future Cardiol 2017;13:279–296. 28581362 10.2217/fca-2017-0019 PMC5941699

[CIT0023] Harmon SD, Kaduce TL, Manuel TD et al Effect of the delta6-desaturase inhibitor SC-26196 on PUFA metabolism in human cells. Lipids 2003;38:469–476. 12848296 10.1007/s11745-003-1086-9

[CIT0024] Hirose K, Payumo AY, Cutie S et al Evidence for hormonal control of heart regenerative capacity during endothermy acquisition. Science 2019;364:184–188. 30846611 10.1126/science.aar2038 PMC6541389

[CIT0025] Honkoop H, de Bakker DE, Aharonov A et al Single-cell analysis uncovers that metabolic reprogramming by ErbB2 signaling is essential for cardiomyocyte proliferation in the regenerating heart. eLife 2019;8:e50163. 31868166 10.7554/eLife.50163 PMC7000220

[CIT0028] Humayun A, Fornace AJ. GADD45 in stress signaling, cell cycle control, and apoptosis. Adv Exp Med Biol 2022;1360:1–22. 35505159 10.1007/978-3-030-94804-7_1

[CIT0029] Ji X, Chen Z, Wang Q et al Sphingolipid metabolism controls mammalian heart regeneration. Cell Metab 2024;36:839–856.e8. 38367623 10.1016/j.cmet.2024.01.017

[CIT0031] Jopling C, Sleep E, Raya M et al Zebrafish heart regeneration occurs by cardiomyocyte dedifferentiation and proliferation. Nature 2010;464:606–609. 20336145 10.1038/nature08899 PMC2846535

[CIT0032] Jopling C, Sune G, Faucherre A et al Hypoxia induces myocardial regeneration in zebrafish. Circulation 2012a;126:3017–3027. 23151342 10.1161/CIRCULATIONAHA.112.107888

[CIT0033] Jopling C, Sune G, Morera C et al p38α MAPK regulates myocardial regeneration in zebrafish. Cell Cycle 2012b;11:1195–1201. 22391208 10.4161/cc.11.6.19637 PMC3679222

[CIT0034] Kikuchi K, Holdway JE, Werdich AA et al Primary contribution to zebrafish heart regeneration by *gata4*_+_ cardiomyocytes. Nature 2010;464:601–605. 20336144 10.1038/nature08804 PMC3040215

[CIT0036] Korbecki J, Bobinski R, Dutka M. Self-regulation of the inflammatory response by peroxisome proliferator-activated receptors. Inflamm Res 2019;68:443–458. 30927048 10.1007/s00011-019-01231-1 PMC6517359

[CIT0037] Koth J, Wang X, Killen AC et al Runx1 promotes scar deposition and inhibits myocardial proliferation and survival during zebrafish heart regeneration. Development 2020;147:dev186569. 32341028 10.1242/dev.186569 PMC7197712

[CIT0039] Li Y, Li Z, Zhang C et al Cardiac fibroblast-specific activating transcription factor 3 protects against heart failure by suppressing MAP2K3-p38 signaling. Circulation 2017;135:2041–2057. 28249877 10.1161/CIRCULATIONAHA.116.024599 PMC5542579

[CIT0038] Li X, Wu F, Günther S et al Inhibition of fatty acid oxidation enables heart regeneration in adult mice. Nature 2023;622:619–626. 37758950 10.1038/s41586-023-06585-5 PMC10584682

[CIT0041] Lin YH, Zhang S, Zhu M et al Mice with increased numbers of polyploid hepatocytes maintain regenerative capacity but develop fewer hepatocellular carcinomas following chronic liver injury. Gastroenterology 2020;158:1698–1712.e14. 31972235 10.1053/j.gastro.2020.01.026 PMC8902703

[CIT0043] Magadum A, Ding Y, He L et al Live cell screening platform identifies PPARdelta as a regulator of cardiomyocyte proliferation and cardiac repair. Cell Res 2017;27:1002–1019. 28621328 10.1038/cr.2017.84 PMC5539351

[CIT0044] Maillet M, Purcell NH, Sargent MA et al DUSP6 (MKP3) null mice show enhanced ERK1/2 phosphorylation at baseline and increased myocyte proliferation in the heart affecting disease susceptibility. J Biol Chem 2008;283:31246–31255. 18753132 10.1074/jbc.M806085200 PMC2576531

[CIT0045] Meng X, Zhang L, Han B et al PHLDA3 inhibition protects against myocardial ischemia/reperfusion injury by alleviating oxidative stress and inflammatory response via the Akt/Nrf2 axis. Environ Toxicol 2021;36:2266–2277. 34351043 10.1002/tox.23340

[CIT0046] Mukherjee D, Wagh G, Mokalled MH et al Ccn2a is an injury-induced matricellular factor that promotes cardiac regeneration in zebrafish. Development 2021;148:dev193219. 33234717 10.1242/dev.193219 PMC7847265

[CIT0047] Oppedisano F, Macrì R, Gliozzi M et al The anti-inflammatory and antioxidant properties of n-3 PUFAs: their role in cardiovascular protection. Biomedicines 2020;8:306. 32854210 10.3390/biomedicines8090306 PMC7554783

[CIT0048] Patterson M, Barske L, Van Handel B et al Frequency of mononuclear diploid cardiomyocytes underlies natural variation in heart regeneration. Nat Genet 2017;49:1346–1353. 28783163 10.1038/ng.3929 PMC5736145

[CIT0049] Picelli S, Faridani OR, Bjorklund AK et al Full-length RNA-seq from single cells using Smart-seq2. Nat Protoc 2014;9:171–181. 24385147 10.1038/nprot.2014.006

[CIT0050] Piquereau J, Ventura-Clapier R. Maturation of cardiac energy metabolism during perinatal development. Front Physiol 2018;9:959. 30072919 10.3389/fphys.2018.00959 PMC6060230

[CIT0051] Porrello ER, Mahmoud AI, Simpson E et al Transient regenerative potential of the neonatal mouse heart. Science 2011;331:1078–1080. 21350179 10.1126/science.1200708 PMC3099478

[CIT0052] Poss KD, Wilson LG, Keating MT. Heart regeneration in zebrafish. Science 2002;298:2188–2190. 12481136 10.1126/science.1077857

[CIT0053] Schmidt-Schweda S, Holubarsch C. First clinical trial with etomoxir in patients with chronic congestive heart failure. Clin Sci (Lond) 2000;99:27–35. 10887055

[CIT0054] Shi Y, Tian M, Zhao X et al α-Ketoglutarate promotes cardiomyocyte proliferation and heart regeneration after myocardial infarction. Nat Cardiovasc Res 2024;3:1083–1097. 39223390 10.1038/s44161-024-00531-y

[CIT0055] Shih YC, Chen CL, Zhang Y et al Endoplasmic reticulum protein TXNDC5 augments myocardial fibrosis by facilitating extracellular matrix protein folding and redox-sensitive cardiac fibroblast activation. Circ Res 2018;122:1052–1068. 29535165 10.1161/CIRCRESAHA.117.312130 PMC5899016

[CIT0056] Shoffner A, Cigliola V, Lee N et al Tp53 suppression promotes cardiomyocyte proliferation during zebrafish heart regeneration. CELL REP 2020;32:108089. 32877671 10.1016/j.celrep.2020.108089 PMC7494019

[CIT0057] Tahara N, Akiyama R, Wang J et al The FGF-AKT pathway is necessary for cardiomyocyte survival for heart regeneration in zebrafish. Dev Biol 2021;472:30–37. 33444612 10.1016/j.ydbio.2020.12.019 PMC7956161

[CIT0059] Tan J, Yang M, Wang H et al Moderate heart rate reduction promotes cardiac regeneration through stimulation of the metabolic pattern switch. CELL REP 2022;38:110468. 35263588 10.1016/j.celrep.2022.110468

[CIT0061] Verlengia R, Gorjao R, Kanunfre CC et al Effects of EPA and DHA on proliferation, cytokine production, and gene expression in Raji cells. Lipids 2004;39:857–864. 15669761 10.1007/s11745-004-1307-2

[CIT0062] von Gise A, Lin Z, Schlegelmilch K et al YAP1, the nuclear target of Hippo signaling, stimulates heart growth through cardiomyocyte proliferation but not hypertrophy. Proc Natl Acad Sci USA 2012;109:2394–2399. 22308401 10.1073/pnas.1116136109 PMC3289361

[CIT0064] Wang W, Hu CK, Zeng A et al Changes in regeneration-responsive enhancers shape regenerative capacities in vertebrates. Science 2020;369:eaaz3090. 32883834 10.1126/science.aaz3090 PMC9479427

[CIT0063] Wang L, Liu J, Wang Z et al Dexmedetomidine abates myocardial ischemia reperfusion injury through inhibition of pyroptosis via regulation of miR-665/MEF2D/Nrf2 axis. Biomed Pharmacother 2023;165:115255. 37549462 10.1016/j.biopha.2023.115255

[CIT0065] Wilkinson PD, Delgado ER, Alencastro F et al The polyploid state restricts hepatocyte proliferation and liver regeneration in mice. Hepatology 2019;69:1242–1258. 30244478 10.1002/hep.30286 PMC6532408

[CIT0066] Ye S, Zhao T, Zhang W et al p53 isoform Delta113p53 promotes zebrafish heart regeneration by maintaining redox homeostasis. Cell Death Dis 2020;11:568. 32703938 10.1038/s41419-020-02781-7 PMC7378207

[CIT0067] Yu P, Song S, Zhang X et al Downregulation of apoptotic repressor AVEN exacerbates cardiac injury after myocardial infarction. Proc Natl Acad Sci USA 2023;120:e1992485176. 10.1073/pnas.2302482120 PMC10589712 37816050

[CIT0070] Zhao Y, Yang G, Wu N et al Integrated transcriptome and phosphoproteome analyses reveal that fads2 is critical for maintaining body LC-PUFA homeostasis. J Proteomics 2020;229:103967. 32891890 10.1016/j.jprot.2020.103967

[CIT0069] Zhao Y, Lv H, Yu C et al Systemic inhibition of mitochondrial fatty acid beta-oxidation impedes zebrafish ventricle regeneration. Biochim Biophys Acta Mol Basis Dis 2024;1870:167442. 39059593 10.1016/j.bbadis.2024.167442

[CIT0071] Zhou X, Zhang C, Wu X et al Dusp6 deficiency attenuates neutrophil-mediated cardiac damage in the acute inflammatory phase of myocardial infarction. Nat Commun 2022;13:6672. 36335128 10.1038/s41467-022-33631-z PMC9637103

