# Supplementary material for: Accumulation of newly synthesized docosahexaenoic acid plays an essential role in heart regeneration
Source: Protein Cell. 2025 Aug 20;17(1):5–26. doi: 10.1093/procel/pwaf062 (PMC12888925; doi:10.1093/procel/pwaf062)
Supplement: pwaf062_Supplementary_Materials [file pwaf062_supplementary_materials.zip › pwaf062_Supplementary Information.docx]

**Materials and methods**

**Animals**

Zebrafish were raised and maintained at Zhejiang University in standard zebrafish units as described previously(Gong et al.,2015; Ye et al.,2020). Mice were housed in a temperature-controlled environment under a 12 hour (hr) light:dark cycle with free access to water and food.

Zebrafish *Tg(Δ113p53:GFP)* transgenic line were generated in our previous studies(Chen et al.,2009). Zebrafish AB stain was used to generated *cpt1ab^-/-^* and *ppardb* *^-/-^* mutants. The gRNA sequences were listed in Table S1. Zebrafish *fads2^-/-^* mutants was a gift from Yonghua Sun’s lab and contain 4bp-deletion, resulting a PTC at 338 aa. Adult zebrafish heart amputation experiments were performed in zebrafish 6 months to 12 months in age. Mouse C57BL/6 strain was used as the wild type (WT) animal in all experiments and purchased from Hangzhou Hangsi Biotechnology CO Company. The LAD ligation experiment was performed in 6- to 12-week-old male mice. The heart resection experiment was performed in p1-p8 neonatal mice.

**Adult zebrafish heart resection**

Ventricular surgery was performed on 6- to 12-month-old zebrafish according to previously described procedures. Briefly, zebrafish were anaesthetized with 0.02% Tricaine and then subjected to ~15% ventricular amputation at the apex with scissors. Sham procedures excluded apex resection.

**Heart apex resection in neonatal mice**

P2 neonatal mice were anesthetized by hypothermia on ice for 4 min while P8 mice were anesthetized for 2.5min. Lateral thoracotomy at the fourth intercostal space was performed by blunt dissection of the intercostal muscles after skin incision. An incision was made at the fourth intercostal space and steady pressure was applied to exteriorize the heart out of the chest. The ventricle apex was amputated. Subsequently, the chest and skin incisions were sewn up with an 8-0 prolene suture. The neonates were warmed under a heat bed for several minutes until the pups came to life. In the sham controls, we performed the same procedures without truncating the heart apex.

**Adult mice myocardial infarction**

Adult male mice were anesthetized by intraperitoneal injection of 4% chloral hydrate and supplied with a ventilator (Nanjing Karwin Biotechnology，KW-10). The thoracic cavity was opened at the fourth intercostal space to expose the mouse heart. A suture was placed around the LAD coronary artery to inflict myocardial ischemia on the left ventricle. Then, the incision was sewed up with a 6-0 prolene suture, and the mice were warmed under a heat bed until they came to life. In the sham controls, we performed the same procedures without suturing the LAD coronary artery.

**Drug administration in vivo**

For the experiments in zebrafish, 15 μl of 40 μM Etomoxir (CPT1a inhibitor, MCE, HY-5020), 15 μl of 200 μM Baicalin (CPT1 activator, MCE, HY-N0197), 15 μl of 200 μM DHA (MCE, HY-B2167), and 15 μl of 200 μM sc-26196 (Fads2 inhibitor, MCE, HY-107410）were intraperitoneally injected in each fish at 4 to 6 dpa.

For the experiments in mice, neonatal mice were weighed and intraperitoneally injected daily with DMSO (at the same concentration as drug in PBS) or 3 mg/kg sc-26196 (200 μM) (Fads2 inhibitor, MCE, HY-107410) or 1 mg/kg DHA (200 μM), and adult mice were intraperitoneally injected with DMSO or 1 mg/kg DHA (200 μM) or 0.25mg/kg GSK3787 (100 μM) (PPARD inhibitor, MCE, HY-15577), scheduled as the schematic outline showed.

**Echocardiography**

Cardiac function was assessed by an ultrasound system Vinno 6LAB (Vinno Technology) before surgery and at 7, 14 and 28 dpMI. Animals were anesthetized with 0.5-1.0% isofluorane and hair was removed over the measurement area. The mice were then placed in a supine position on a heating pad. To measure ejection fraction and fractional shortening, short axis images were acquired at the level of the papillary muscle with M-mode.

**AAV9 package and delivery**

Sequence of *Ppard* shRNA(GAAGGCCTTCTCTAAGCACAT) and *Fads2* shRNA (GCGTTTCTTCTACACCTACAT) were cloned into the AAV9 plasmid (pAAV-U6-EGFP). Viruses were generated by GenePharma Co. Ltd, China. AAV9-*Fads2*-shRNA were hypodermic injected for 20μl in Neonatal mice at 1dpa in a dose of 1×10^13^（vg/ml） viral particles per animal. AAV9-*Ppard*-shRNA were tail vein injected for 100μl in adult mice in a dose of 2×10^12^（vg/ml） viral particles per animal.

**Zebrafish cardiomyocyte (CM) isolation and FACS**

The protocal was modified and based on previous study(Hou et al.,2025). Total 6 hearts were isolated from *Tg(Δ113p53:GFP)* transgenic zebrafish with cardiosurgery at 7 dpa. The atrium and bulbus arteriosus were removed from the hearts. Ventricles were minced to small pieces in perfushion buffer (1XPBS, 10 mM HEPES, 30 mM taurine, 5.5 mM glucose and 10 mM BDM) and subsequently incubated in 1 ml Bacillus Licheniformis protease (Creative Enzymes, NATE0633, 5mg/ml) at 4℃ for 2 h. The suspension was centrifuged at 250g for 5 min at 4℃. The 0.5 ml supernatant was removed and the remain suspension were added with 0.5 ml stopping buffer (5% BSA in perfushion buffer) to stop the digestion. The cell suspension was filtered through a 70 um cell strainer on ice and then subjected for FACS (MoFlo XDP). About 1900 GFP^+^ and GFP^-^ cells were sorted out for SMART-seq.

**Isolation of neonatal rat ventricle cardiomyocytes (NRVMs) and neonatal rat cardiac fibroblasts (NRCFs)**

Ventricles were isolated from P2-3 neonatal Sprague Dawley rats and minced to small pieces in cold PBS. The tissues were removed to 50mL centrifugal tubes containing digestion buffer (1mg/ml collagenase II; Trypsin) and digested in shakers at 37℃ 4-6 times. After each 30-40min digestion, supernatant were removed to 10％ FBS DMEM medium and new digestion buffer were added. The digested cells were filtrated by 70 and 100μM cell sieve and then centrifuged and resuspended by complete medium. Cell suspension were plated for fibroblast adhesion for 2h. Supernatant which contains most of NRVMs were collected and plated on the culture dish. NRVMs were cultured in 37℃ cell incubator for 36h and followed by the treatment. Adherent NRCFs and NRVMs were cultured in 37℃ cell incubator for 24h or 36h and followed by the treatment .

**Cell culture and treatment**

NRVMs and 293T cells were cultured in DMEM (Sigma-Aldrich, D5796) with 10% fetal bovine serum and 1% penicillin/streptomycin. For NRVMs treatment, cells were treated with DHA(0.5-10μM) or sc-26196(1μM) or GSK3787(1μM) or OA(Solarbio, O8291)(0.5μM) for 24h. For NRCFs treatment, cells were treated with DHA(0.5-10μM) or GSK3787(1μM) for 24h . For 293T cells treatment, cells were treated with DHA(1μM) or OA(1μM) for 24h after transfection.

**Plasmid construction and transfection**

The full length CDS of mouse PPARD was cloned into PCS2 vector and 1.5-2k bp DNA fragments around PPARD peaks of mouse *Mef2d* and *Phlda3* genes were cloned into Pgl3-basic vector. Predicted PPARD binding motif in the *Mef2d* and *Phlda3* promoters were mutated into into oligo-T with the same number of nucleotides. Key binding amino acids of DHA or OA in PPARD were mutated to Alanine. About 1μg Plasmids were transfected into 293T cells in 6 well plates by PolyJet Transfection Reagent(SignaGen) fo 24h.

**siRNA transfection**

siRNA oligos were synthesized by GenePharma. After 36h plated, NRVMs were washed by PBS for 3 times. All the siRNA were transfected at 50 nM by GenMute™ siRNA Transfection Reagent (SignaGen). After 24 hours of siRNA transfection, cells were treated or detected for different assays.

**Luciferase aasay**

PPARD-PCS2 and *Mef2d*-p reporter or *Phlda3*-p reporter were co-transfected into 293T cells for 24h. Cells tranfected with NC-PCS2 were used for negative control and cells without transfection were set for blank control. Cells were then treated with DHA or OA for 24h. Luciferase assay were conducted by Luciferase Reporter Gene Assay Kit (Yeasen,11401ES76).

**EdU incorporation assay**

For the assays in zebrafish, 3 μL of 0.5 M EdU (Invitrogen, A10044) was intraperitoneal injected into each zebrafish once daily for 3 days from 4 to 6 dpa. For the assays in mice, the mice after MI were intraperitoneal injected with 50 mg/kg EdU, scheduled as the schematic outline showed. The hearts were fixed for cryosectioning. For the assays in NRCFs, cells were treated with EdU(20μM) for 2h. EdU staining was performed using Azide Alexa Fluor 647 (Invitrogen, A10277).

**In situ hybridization**

In situ hybridization assay was performed as described previously (Zhao et al.,2021). Isolated hearts of mice and zebrafish were fixed in 4% PFA at 4℃ overnight and cryosectioned into 8 and 10 µm respectively. DIG-labeled RNA probes were generated by NEB T7 RNA Polymerase (M0251S) and Roche DIG RNA Labelling Mix (11277073910). Fresh cryo-sections were hybridizated with DIG-labeled RNA probes(6-8μg/ml) at 68 or 70℃ overnight. Then the sections were washed by 5×SSCT, 0.5×SSCT, 1×MABT and blocked by 1×DIG block buffer (Roche, 11096176001). Staining was performed with Anti-Digoxigenin-AP (Roche, 11093274910, 1:2000) and the BCIP/NBT Alkaline Phosphatase Colour Development Kit (Beyotime Biotechnology, C3206). Fluorescein-labeled RNA probes were generated by NEB T7 RNA Polymerase (M0251S) and Roche Fluorescein RNA Labelling Mix (11685619910). Staining was performed with Anti-Fluorescein-AP (Roche, 11426338910, 1:2000) and the SIGMAFAST Fast Red TR/Naphthol AS-MX Tablets (1003318421). Dual-color in situ hybridization entails simultaneous incubation of two different labelled probes followed by sequential staining of the two detection systems. The primers for each probes were listed in Table S1.

**Oil red O staining and signal quantification**

Defrosted fresh cryo-sections (10μm）were stained with Oil Red O solution (sigma,01516) for 15 mins at room temperature and washed by flowing water for 5 s, and then transferred into redyeing solution (Nanjing Jiancheng technology,D207-1-3) for 2 mins. The sections were washed for 5 s and sealed for photographing immediately. For signal quantification, 3-4 sections from each animal were used. Blue color in pictures was removed by Image J. Then injury area was selected for intensity calculation of red color. Area without Oil Red O signals in injury site was selected for calculating the intensity of blank background in picture. The relative Oil Red O signal intensity was obtained by the signal intensity of injury area minus the signal intensity of blank background.

**Masson staining**

For the staining in zebrafish, 4-6 serial sections around injury sites were collected for each individual heart. The average of scar area was calculated from 10-13 hearts of each group and 4 sections of each heart. For the staining in neonatal mice, 3-4 serial sections around injury sites were collected for each individual heart. About 3 sections per heart and 7-9 hearts per sample were analyzed for the average of scar area. For the staining in adult mice, 6 sections from different layers at 200 μM interval were collected for each heart. Adult mice scar areas were quantified based on previous study(Takagawa et al.,2007). Masson staining and photograph were performed by Haoke Biotechnology CO.

**WGA staining**

Cryo-sections of hearts were incubated in 5 μg/ml Wheat Germ Agglutinin, Oregon Green 488 Conjugate (Invitrogen, W6748) for 15 min and were stained with DAPI. To quantify CM size, average of cell area in the images was analyzed with Image J. Four sections per heart and 5-6 hearts per group were used for quantification.

**Quantitative real-time PCR (qRT-PCR)**

Hearts were freshly isolated from anaesthetized zebrafish subjected to sham surgery or resection at different experiments. The outflow tracts and atriums were removed from the isolated hearts. Total RNA was isolated from approximately 4-6 isolated ventricles using TRIZOL reagent (AidLab) according to the manufacturer’s protocol. In NRVMs, total RNA was isolated from 6-well plate. In mice, total RNA was isolated from half of the heart from the animal. About 1 μg RNA digested with DNase I (NEB) was used for reverse transcription using the M-MLV Reverse Transcritptase (Invitrogen) according to the manufacturer’s protocol. Quantitative PCR was performed in a CFX96TM Real-Time System (Bio-Rad) using a C1000 Thermal Cycle (Bio-Rad) according to the manufacturer’s instructions. *rpl32* was used for normalization of cDNA of sorting cells to confirm RNA-seq results. *actb1* was used for normalization of cDNA of zebrafish embryos and NRVMs. *gapdh* was used for normalization of cDNA of zebrafish adult hearts and mouse hearts. Statistics were obtained from three repeats. The information of qPCR primers was listed in Supplementary table S1.

**Immunostaining and immunohistochemistry**

For the immunostaining and immunohistochemistry assay , zebrafish and mouse hearts were fixed, cryosectioned (10 and 8 μm respectively). NRVMs were plated on coverslips. Primary antibodies used in zebrafish were anti-MF20 (Developmental Studies Hybridoma Bank, AB2147781), and primary antibodies used in mice were anti-PH3 (Santa cruz, sc-8656-R), anti-TNNT2 (ABclonal, A4914), anti-CTNT (ThermoFisher, MA5-12960), anti-Ki67 (ABclonal, A23722), anti-CD45(Servicebio,GB11066), anti-FADS2 (Affinity Biosciences, DF15514), anti-CD68 (Cell Signaling, 97778S), anti-Arg1 (ABclonal, A25808), anti-α-SMA (Boster, BM0002), anti-F4/80 (ThermoFisher, 11-4801-82), anti-Vimentin (ABclonal, A19607), anti-PCM1 (Sigma-Aldrich, HPA023374). Secondary antibodies were anti-mouse IgG H&L Alexa Fluor 488 (Abcam, ab150113), anti-mouse IgG H&L Alexa Fluor 647 (Abcam, ab150115), anti-Rabbit IgG H&L Alexa Fluor 647 (Abcam, ab150143),anti-rabbit IgG-HRP(Servicebio,GB23303). Nuclei were stained by DAPI (BYT, C1002).

**Western blot**

For western blot in adult mouse injured heart, 1/2 hearts above the infarction area were isolated for protein extraction by RIPA and SDS lysis buffer. Primary antibodies were anti-PPARD (Santa cruz, sc-74517), anti-FADS2 (Affinity Biosciences, DF15514), anti-GAPDH (Proteintech, 60004-1-Ig). The secondary antibodies were goat anti-rabbit IgG (HUABIO, HA1001), goat anti-mouse IgG (HUABIO, HA1006).

**cell wound scratch assay**

For cell wound scratch assay, NRCFs were cultured in complete medium for 24h after plated. Then a scratch was made using a 1mL pipette tip. After PBS washing, the scratch area was immediately captured and remained cells were cultured in complete medium supplied with DHA or GSK3787 for 24h. The scratch area was captured again after 24 hours using a microscope (Keyence). The migration rates were quantified by Image J.

**Liquid chromatography-tandem mass spectrometry (LC-MS) analysis**

For LC-MS in zebrafish, each treatment had 4 replicates. About 15-25 ventricles in each replicate were washed with PBS to remove blood, and pooled together to reach 5 mg weight. For LC-MS in mice, each treatment had 4-6 replicates. Single ventricle without blood in each replicate was used for the analysis. The ventricle weights of neonatal and adult mice were 15-25 mg and 90-110 mg respectively. Metabolites extraction, LC-MS analysis and data analysis were performed by Biotree Biomedical Technology CO. Briefly, an UHPLC system (Vanquish, Thermo Fisher Scientific) coupled with Orbitrap Exploris 120 mass spectrometer (Orbitrap MS, Thermo) were used for the LC-MS/MS analysis. The Orbitrap Exploris 120 mass spectrometer was to acquire MS/MS spectra on information-dependent acquisition (IDA) mode with a software (Xcalibur, Thermo). The ESI source conditions were set : sheath gas flow rate as 50 Arb, Aux gas flow rate as 15 Arb, capillary temperature 320 ℃, full MS resolution as 60000, MS/MS resolution as 15000 collision energy as 10/30/60 in NCE mode, spray Voltage as 3.8 kV (positive) or -3.4 kV (negative), respectively. The raw data were converted to the mzXML format with Proteo Wizard and processed with a program developed from R based on XCMS for peak detection, extraction, alignment and integration. MS2 database (BiotreeDB) was applied in metabolite annotation. The cutoff for annotation was set at 0.3. The significant changes were set as VIP > 1 and p < 0.05 (Student’s t-test).

**RNA-seq analysis**

For RNA-seq in isolated zebrafish CMs, total RNA was extracted from 1900 GFP^+^- or GFP^-^-CMs sorted with FACS. RNA reverse transcription and cDNA amplification were performed according to the protocol of SMART-seq. SMART-seq was done by Annoroad company (Beijing, China). For bulk RNA-seq, each treatment had 3 replicates in zebrafish, 2 replicates in mice and two replicates in NRVMs. About 4-6 hearts of zebrafish and 1 piece of heart of mice and a 6-well plate of NRVMs in each replicate were used to extract total RNA. Isolation of mRNA, library construction, high throughput sequencing and data filtering were performed by Annoroad company (Beijing, China). Quality control was conducted using FastQC (v0.11.9), after which clean reads were mapped to the zebrafish genome (GRCz11), mouse genome (GRCm39) and rat genome (Rn6) according to the species of the samples, using the software Hisat2 (v2.2.1) with default parameters(Kim et al.,2015). Data sorting and visualization were performed using Samtools (v1.10) and IGV (v2.12.3), respectively. Fragments per kilobase of exon model per million mapped fragments (FPKM) for each gene were analyzed with featureCounts (v2.0.1)(Liao et al.,2014). Differential expression genes (DEGs) analysis were performed using R (v4.3.1) package DESeq2 (v.1.42.0) (|log2FoldChange| ≥1 and P_adj_ < 0.05 for isolated CM RNA-seq; |log2FoldChange| ≥ 0.68 and P_adj_ < 0.05 for bulk RNA-seq)(Love et al.,2014). GO and KEGG analysis was further performed using DAVID Bioinformatics resources (https://david.ncifcrf.gov). GSEA analysis for zebrafish samples was performed using GSEA v4.2.3 with zebrafish GO genesets.

**ChIP-seq analysis**

For ChIP-seq of PPARD in mouse MI hearts at 7dpMI, each treatment had 2 replicates. Each replicate contained two injury hearts with half of the infarction zone and the remaining area. Tissues were crosslinked by formaldehyde for 12min. Chromatin was sheared into 200-500bp DNA fragments by ultrasound (10s on,30s off, 40%, 15min). A PPARD antibody (Santa cruz,sc-74517) was used to perform the precipitation. Then it was eluted from the beads and digested with proteinase K and RNAase I. Finally it was purified by DNA purification kit.

ChIP-seq data were provided by the sequencing service provider and quality control was assessed using FastQC (v0.11.9). Sequence alignment was carried out with BWA (v0.7.17) using the GRCm39 mouse genome as the reference. Data sorting and deduplication were performed using Samtools (v1.10) and Picard (v2.27.1), respectively. Normalization was conducted with deepTools (v3.5.1), and the data were visualized using IGV (v2.12.3). Peak calling was done using MACS2 (v2.2.7.1) with an extension size of 150 and a q-value threshold of 0.1. Peak annotation was carried out using the ChIPseeker (v1.38.0) package in R (v4.3.1). Stable peaks in DMSO and DHA treatments were identified by overlapping peaks detected in two replicates per condition. Peaks exclusive to the DHA treatment were isolated by removing overlapping peaks between the DMSO and DHA groups for downstream analysis. Gene Ontology (GO) analysis of DHA-specific peak-associated genes was performed using the DAVID Bioinformatics Resources (https://david.ncifcrf.gov). These genes were further cross-referenced with differentially expressed genes (DEGs) from our RNA-seq analysis (|FoldChange| > 1.5, p.adjusted < 0.05), and motif analysis was performed using HOMER (v4.11.1).

**Structure prediction,docking and molecular dynamics simulations**

**The structure of PPARδ in complex with OA was predicted using the AlphaFold 3.0 (AF3) web server**(Abramson et al.,2024)**. To model whether PPARδ bound to DHA, a docking approach was employed. Specifically, AutoDock Vina**(Eberhardt et al.,2021) **was used to predict the binding pose of DHA within the AF3-predicted binding pocket of PPARδ. The docking search space was defined based on the ligand-binding domain of PPARδ, with a grid box centered on the binding pocket and dimensions of 2 x 2 x 2 nm^3^. The exhaustiveness parameter was set to 8 to ensure thorough sampling of potential binding poses. The top-ranking binding pose from AutoDock Vina**(Eberhardt et al.,2021) **that also closely matched the AF3-predicted pose was used for subsequent simulations. To minimize computational costs, only the ligand-binding domain (N168-Y440) of PPARδ was included in the models.**

**The models of molecular dynamics simulations were solvated in a cubic water box with dimensions of 9.0 nm and 0.15 M NaCl, resulting in a system of approximately 68,000 atoms. System setup and energy minimization were performed using CHARMM-GUI** (Jo et al.,2008)**, followed by 125 ps of NVT and 1 ns of NPT equilibration. Production MD simulations were conducted under NVT conditions, using the CHARMM36m force field**(Huang et al.,2017) **for proteins and the TIP3P water model. Force field parameters for OA and DHA were derived from the CHARMM General Force Field (CGenFF)**(Vanommeslaeghe et al.,2010)**. During the MD simulations, the temperature was maintained at 300 K. Neighbor searching was performed every 20 steps, with the PME algorithm handling electrostatic interactions using a cut-off of 1.2 nm. A reciprocal grid of 80 x 80 x 80 cells with 4th order B-spline interpolation was used, and a single cut-off of 1.2 nm was applied for Van der Waals interactions. Temperature coupling was managed using the V-rescale algorithm. Four independent 1000 ns MD simulations were conducted for each system using the GPU-accelerated version of Gromacs 2023.3. Trajectory analysis was performed using Gromacs gmx tools, and protein-ligand contact frequencies were calculated with GetContacts (https://getcontacts.github.io/).**

**Statistical analyses**

Sample sizes were designed based on routine genetic analysis in zebrafish and mouse studies. Unless stated otherwise, the experiments were randomized and investigators were not blinded to allocation during experiments. No data were excluded from the analyses. Statistical analysis Unless stated otherwise, all parameters were tested using unpaired two-tailed Student’s t-test. Significant p-value in all statistical analyses was obtained using GraphPad Prism 8 (GraphPad Software). A p-value below 0.05 was considered to be statistically significant (p > 0.05, n.s.. p < 0.05,*.p < 0.01,**. p < 0.001,***).

**References**

Abramson, J., Adler, J., Dunger, J., Evans, R., Green, T., Pritzel, A., Ronneberger, O., Willmore, L., Ballard, A.J., and Bambrick, J., et al.(2024). Accurate structure prediction of biomolecular interactions with AlphaFold 3. NATURE 630, 493-500.

Chen, J., Ng, S.M., Chang, C., Zhang, Z., Bourdon, J.C., Lane, D.P., and Peng, J.(2009). p53 isoform delta113p53 is a p53 target gene that antagonizes p53 apoptotic activity via BclxL activation in zebrafish. Genes Dev 23, 278-290.

Eberhardt, J., Santos-Martins, D., Tillack, A.F., and Forli, S.(2021). AutoDock Vina 1.2.0: New Docking Methods, Expanded Force Field, and Python Bindings. J CHEM INF MODEL 61, 3891-3898.

Gong, L., Gong, H., Pan, X., Chang, C., Ou, Z., Ye, S., Yin, L., Yang, L., Tao, T., and Zhang, Z., et al.(2015). p53 isoform Delta113p53/Delta133p53 promotes DNA double-strand break repair to protect cell from death and senescence in response to DNA damage. CELL RES 25, 351-369.

Hou, J., Song, Y., Xiao, C., Sun, Y., Shen, J., Ma, X., Zhou, Q., Chiu, S.C., Xu, Y., and Huang, Y., et al.(2025). Cloche/Npas4l is a pro-regenerative platelet factor during zebrafish heart regeneration. DEV CELL.

Huang, J., Rauscher, S., Nawrocki, G., Ran, T., Feig, M., de Groot, B.L., Grubmuller, H., and MacKerell, A.J.(2017). CHARMM36m: an improved force field for folded and intrinsically disordered proteins. NAT METHODS 14, 71-73.

Jo, S., Kim, T., Iyer, V.G., and Im, W.(2008). CHARMM-GUI: a web-based graphical user interface for CHARMM. J COMPUT CHEM 29, 1859-1865.

Kim, D., Langmead, B., and Salzberg, S.L.(2015). HISAT: a fast spliced aligner with low memory requirements. NAT METHODS 12, 357-360.

Liao, Y., Smyth, G.K., and Shi, W.(2014). featureCounts: an efficient general purpose program for assigning sequence reads to genomic features. BIOINFORMATICS 30, 923-930.

Love, M.I., Huber, W., and Anders, S.(2014). Moderated estimation of fold change and dispersion for RNA-seq data with DESeq2. GENOME BIOL 15, 550.

Takagawa, J., Zhang, Y., Wong, M.L., Sievers, R.E., Kapasi, N.K., Wang, Y., Yeghiazarians, Y., Lee, R.J., Grossman, W., and Springer, M.L.(2007). Myocardial infarct size measurement in the mouse chronic infarction model: comparison of area- and length-based approaches. J Appl Physiol (1985) 102, 2104-2111.

Vanommeslaeghe, K., Hatcher, E., Acharya, C., Kundu, S., Zhong, S., Shim, J., Darian, E., Guvench, O., Lopes, P., and Vorobyov, I., et al.(2010). CHARMM general force field: A force field for drug-like molecules compatible with the CHARMM all-atom additive biological force fields. J COMPUT CHEM 31, 671-690.

Ye, S., Zhao, T., Zhang, W., Tang, Z., Gao, C., Ma, Z., Xiong, J.W., Peng, J., Tan, W.Q., and Chen, J.(2020). p53 isoform Delta113p53 promotes zebrafish heart regeneration by maintaining redox homeostasis. CELL DEATH DIS 11, 568.

Zhao, T., Ye, S., Tang, Z., Guo, L., Ma, Z., Zhang, Y., Yang, C., Peng, J., and Chen, J.(2021). Loss-of-function of p53 isoform Delta113p53 accelerates brain aging in zebrafish. CELL DEATH DIS 12, 151.

**Supplementary materials**

**
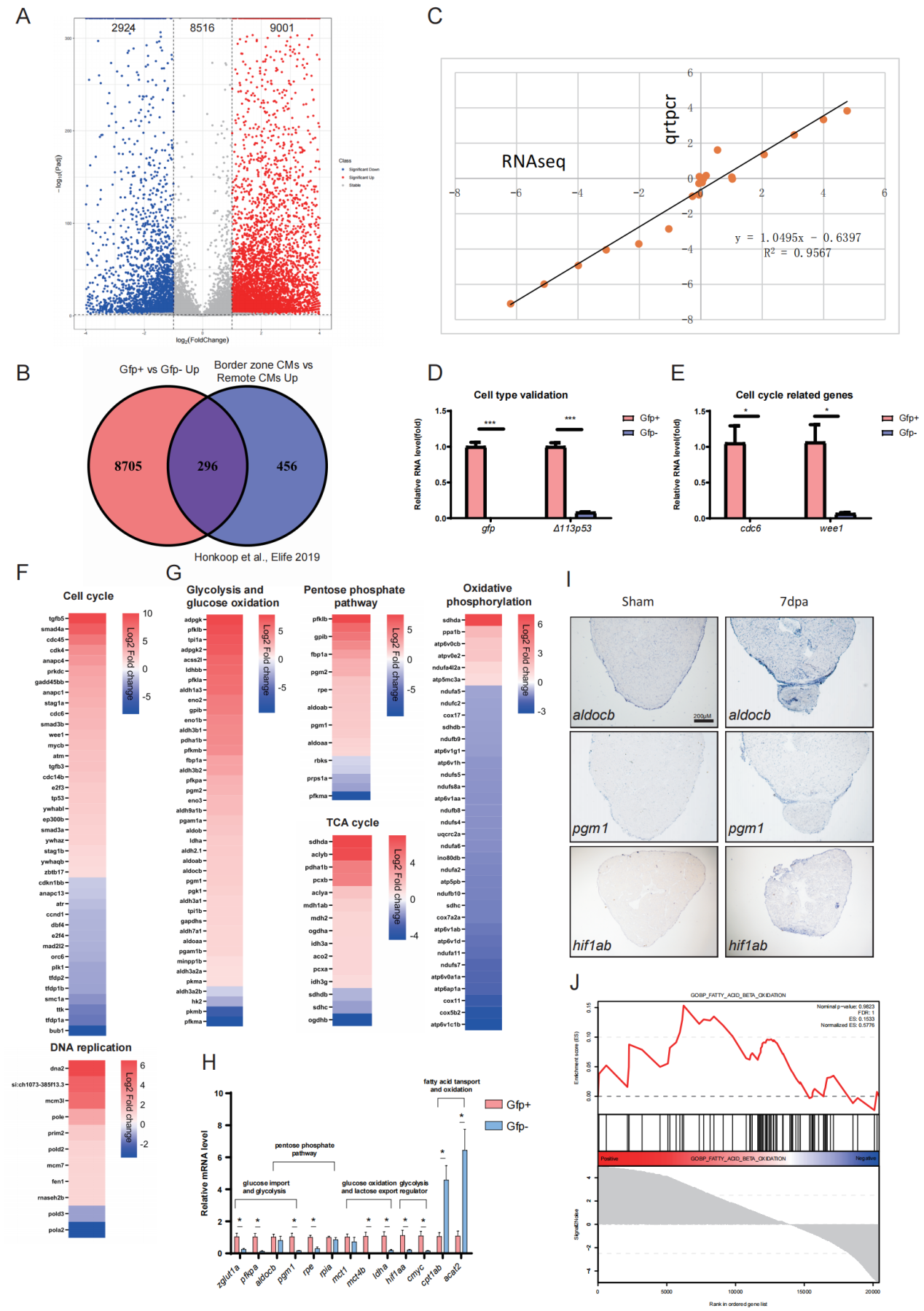
**

**Supplementary Figure 1. ∆113p53^+^ cells are proliferating CMs and undergo the metabolic switch.**

**A** Volcano plot showing the DEGs (|FoldChange|≥2, P_adj_ < 0.05) of ∆113p53^+^ CMs versus ∆113p53^-^ CMs. ∆113p53^+^ and ∆113p53^-^ CMs were isolated and analyzed as described in **Figure 1A**.

**B** The venn diagram showing that the upregulated genes identified in our ∆113p53^+^ cells overlapped with the upregulated genes in proliferating CMs identified by single cell sequencing of a previous study.

**C** Confirmation of the RNA-seq data by the qRT-PCR analysis of 20 randomly selected genes (6 upregulated genes, 6 downregulated and 8 without significantly changed genes) (|log2FC|≥1，Padj < 0.05) in ∆113p53^+^ and ∆113p53^-^ CMs. Expression data of genes detected by RNA-seq was plotted against those by qRT-PCR. The reference line indicates the linear correlation between the RNA-seq and qRT-PCR.

**D,E** qRT-PCR was performed to validate the expression of *Gfp*, *∆113p53*, *cdc6* and *wee1* in ∆113p53^+^ and ∆113p53^-^ CMs.

**F,G** Heatmaps of DEGs of cell cycle and DNA replication related genes (**F**) and metabolic pathways related genes (**G**) in ∆113p53^+^ and ∆113p53^-^ CMs.

**H** Validation of the RNA-seq data by qRT-PCR analysis on 13 DEGs related to metabolism in ∆113p53^+^ and ∆113p53^-^ CMs.

**I** RNA *in situ* hybridization to validate RNA-seq data. The experiment was performed with the DIG-labeled probe to detect *aldocb*, *pgm1*, *hif1ab* on cryosections of zebrafish injury hearts at sham and 7 dpa. The representative picture was taken from 10 hearts in each group. Scale bar: 200 μm.

**J** GSEA analysis on DEGs of the fatty acid oxidation pathway in ∆113p53^+^ and ∆113p53^-^ CMs.

Statistical analysis was performed by Student’s two-tailed unpaired t test in GraphPad Prism 8. The p values were represented by n.s. and asterisks. n.s., p > 0.05; *, p < 0.05.

**
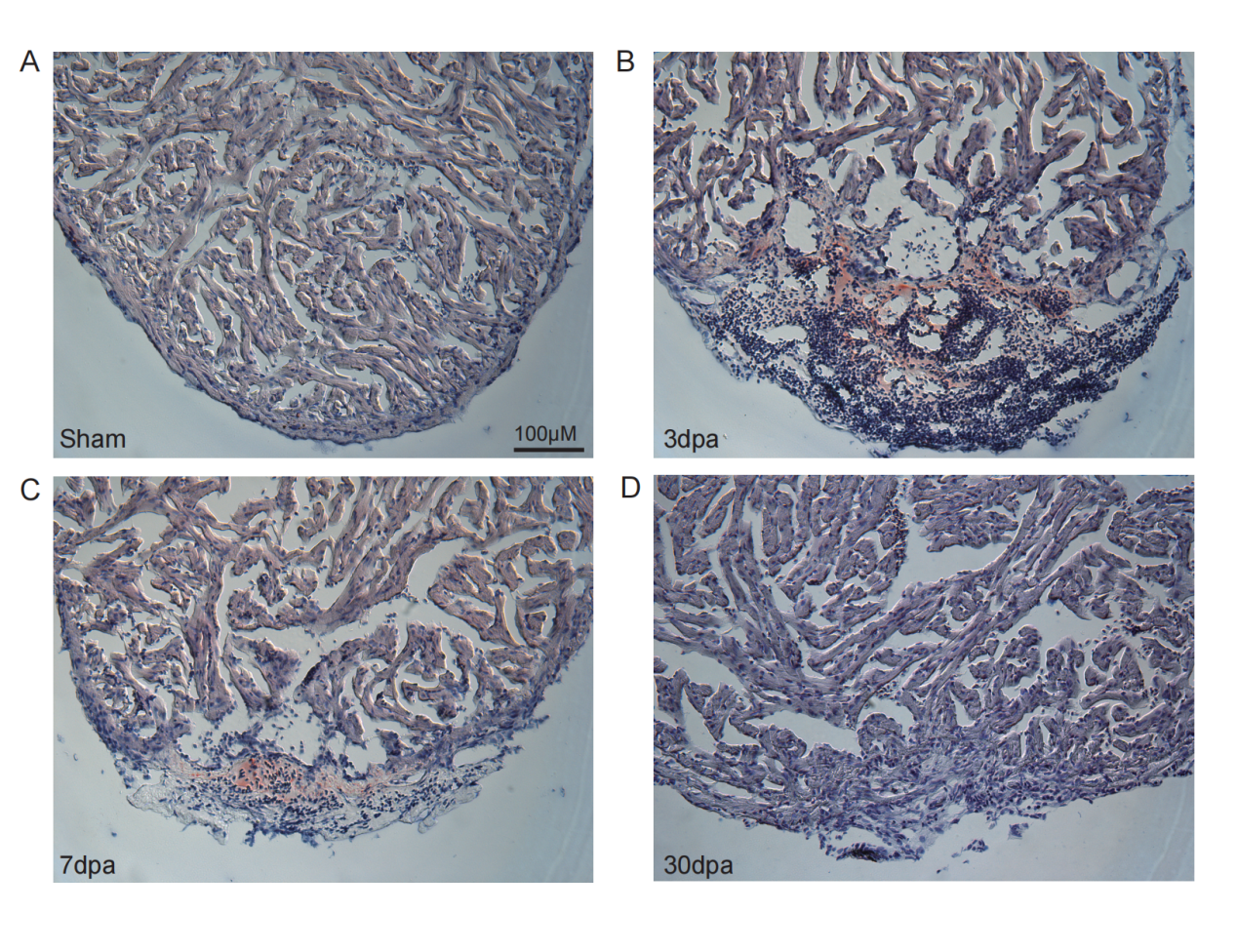
**

**Supplementary Figure 2. Dynamic changes of lipid accumulation during zebrafish heart regeneration.**

Representative images of Oil Red O staining in zebrafish sham (**A**) and injury hearts at 3 (**B**), 7 (**C**) and 30 dpa (**D**). n: 10 hearts/sample; Scale bars: 100 μm.

**
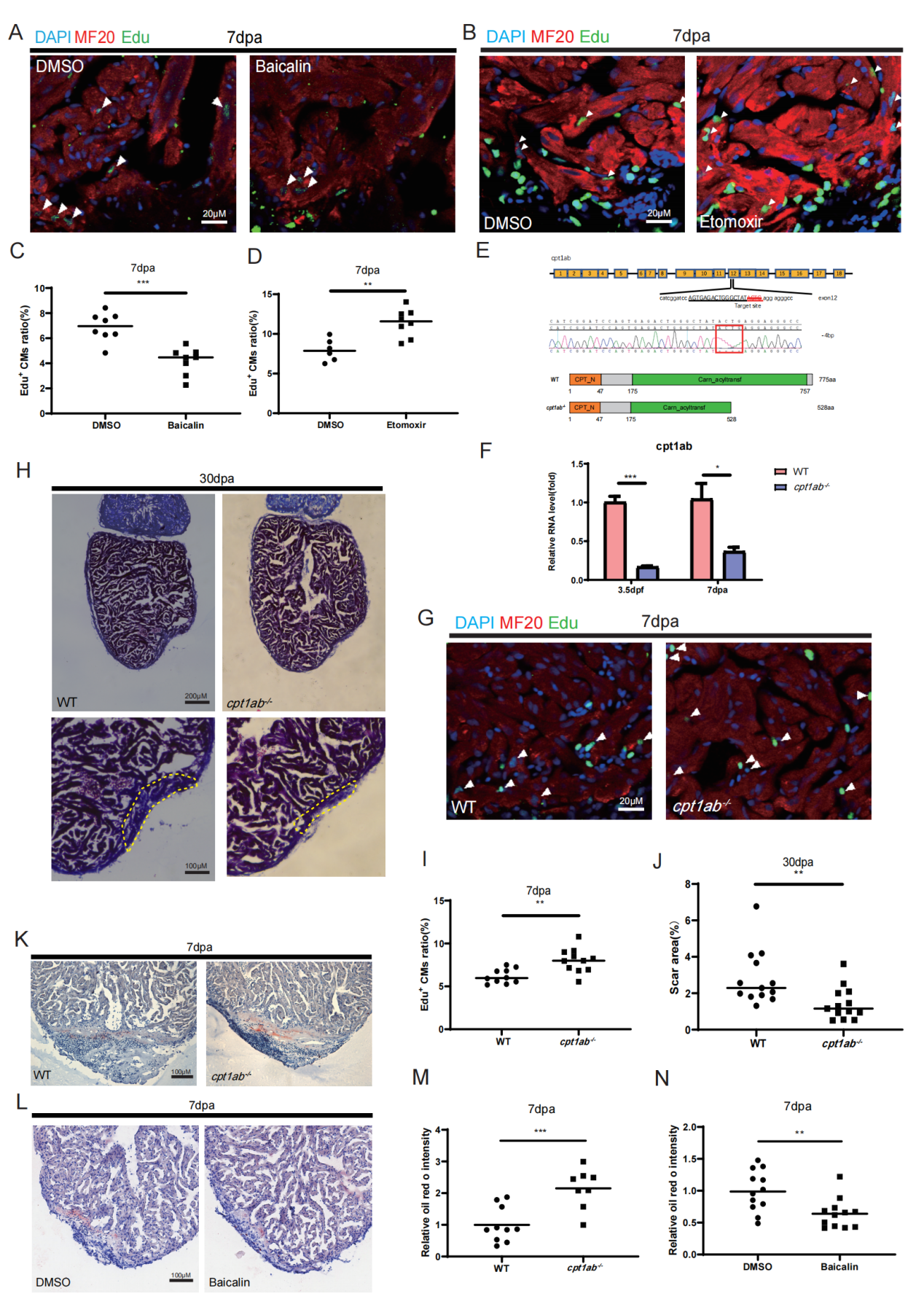
**

**Supplementary Figure 3. Inhibition of Cpt1 promotes zebrafish heart regeneration.**

**A,B** Cryosections of Edu-labelled (in green) of zebrafish injury hearts with different treatments at 7 dpa were immunostained by anti-MF20 (in red). The nuclei were stained with DAPI (in blue). The zebrafish with heart resection were intraperitoneally injected with DMSO (the injection control), Baicalin (CPT1a activator)(**A**) and Etomoxir (CPT1a inhibitor)(**B**). Scale bar: 20 μm.

**C,D** Statistical analyses of EDU^+^ CMs in (**A**) and (**B**). The number of EDU^+^ CMs was counted from 4 sections in the injury site of each heart and was presented as the percentage of the total MF20^+^ cells at the injury sites. Each dot represents an individual heart. n: 6-8 hearts/sample.

**E** Diagram showing the gRNA targeting site and 4-bp deletion in the exon 12 of *cpt1ab* mutant, which results in a premature stop codon (PTC) at 528 aa.

**F** qRT-PCR was performed to exam the expression of *cpt1ab* in WT and *cpt1ab^-/-^* mutant embryos at 3.5 dpf as well as in WT and *cpt1ab^-/-^* mutant injury hearts at 7 dpa.

**G** Immuno-staining of MF20 (in red) and EDU incorporation assay (in green) of WT and *cpt1ab^-/-^* injury hearts at 7dpa. n: 10-11 hearts/sample; Scale bar: 20 μm.

**H** Fibrin clot stained with Masson’s trichrome on the cryosections of WT and *cpt1ab^-/-^* injury hearts at 30 dpa. Yellow dotted lines indicate the approximate scar area. Scar areas were magnified in lower panels. Scale bar: 200 μm or 100 μm respectively for upper and lower panels. n: 13 hearts/sample.

**I,J** Statistical analyses of EDU^+^ CMs and scar areas in (**G**) and (**H**). The number of EDU+ CMs on heart sections was presented as the percentage of the total MF20^+^ cells at the resection sites (**G**). Average scar area with fibrin clots on heart sections was presented as the percentage of the total ventricular area (**H**). Each dot represents an individual heart.

**K** Oil Red O staining of WT and *cpt1ab^-/-^* injury hearts at 7dpa. Scale bar: 100 μm.

**L** Oil Red O staining of WT injury hearts with different treatments at 7 dpa. The WT zebrafish with heart resection were intraperitoneally injected with DMSO (the injection control) or Baicalin. Scale bar: 100 μm.

**M** Statistical analysis of relative Oil Red O signal intensity in injury areas in (**K**). Each dot represents an individual heart. n: 8-10 hearts/sample.

**N** Statistical analysis of relative Oil Red O signal intensity in (**L**). Each dot represents an individual heart. n: 12 hearts/sample.

The experiments were repeated independently for at least 2-3 times with similar results. Statistical analysis was performed by Student’s two-tailed unpaired t test in GraphPad Prism 8. The p values were represented by n.s. and asterisks. n.s., p > 0.05; *, p < 0.05; **, p < 0.01; ***, p < 0.001.

**
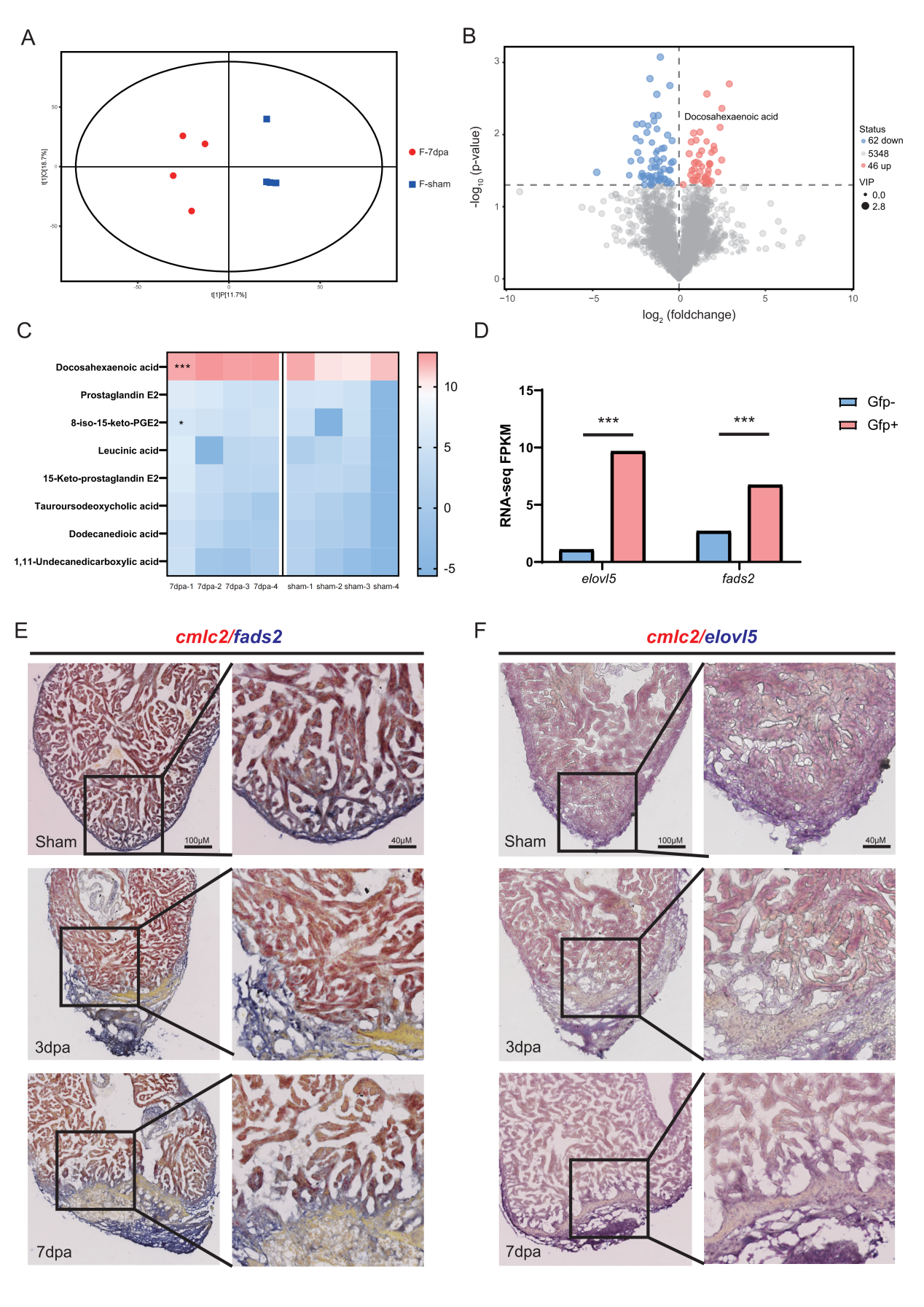
**

**Supplementary Figure 4. DHA increases the most among lipids or derivatives increased in zebrafish injury hearts at 7 dpa.**

**A** OPLS-DA analysis. LC-MS data from four independent replicates including zebrafish sham and injury hearts at 7 dpa, were subjected to OPLS-DA analysis. About 15-25 ventricles in each replicate were pooled together to reach 5 mg weight and subjected for LC-MS analysis.

**B** Volcano plot showing the differentially accumulated metabolites (VIP≥1, P-value< 0.05) in zebrafish sham hearts versus injury hearts at 7dpa.

**C** Heatmap of increased lipids or derivatives in zebrafish injury hearts at 7dpa (fold change>1.5).

**D** FPKMs of *elovl5* and *fads2* in ∆113p53^+^ and ∆113p53^-^ CMs from RNA-seq.

**E,F** Fluorescein-labeled *cmlc2* probe (in red) combined with DIG-labeled *fads2*(**E**) or *elovl5*(**F**) probe (in blue) were used to perform double-RNA in situ hybridization on cryosections of zebrafish WT hearts at sham, 3 and 7 dpa. The representative picture was taken from 6 hearts in each group. Scar areas were magnified in lower panels. Scale bar: 100μm.

Statistical analysis was performed by Student’s two-tailed unpaired t test in GraphPad Prism 8. The p values were represented by n.s. and asterisks. n.s., p > 0.05; *, p < 0.05; **, p < 0.01; ***, p < 0.001.

**
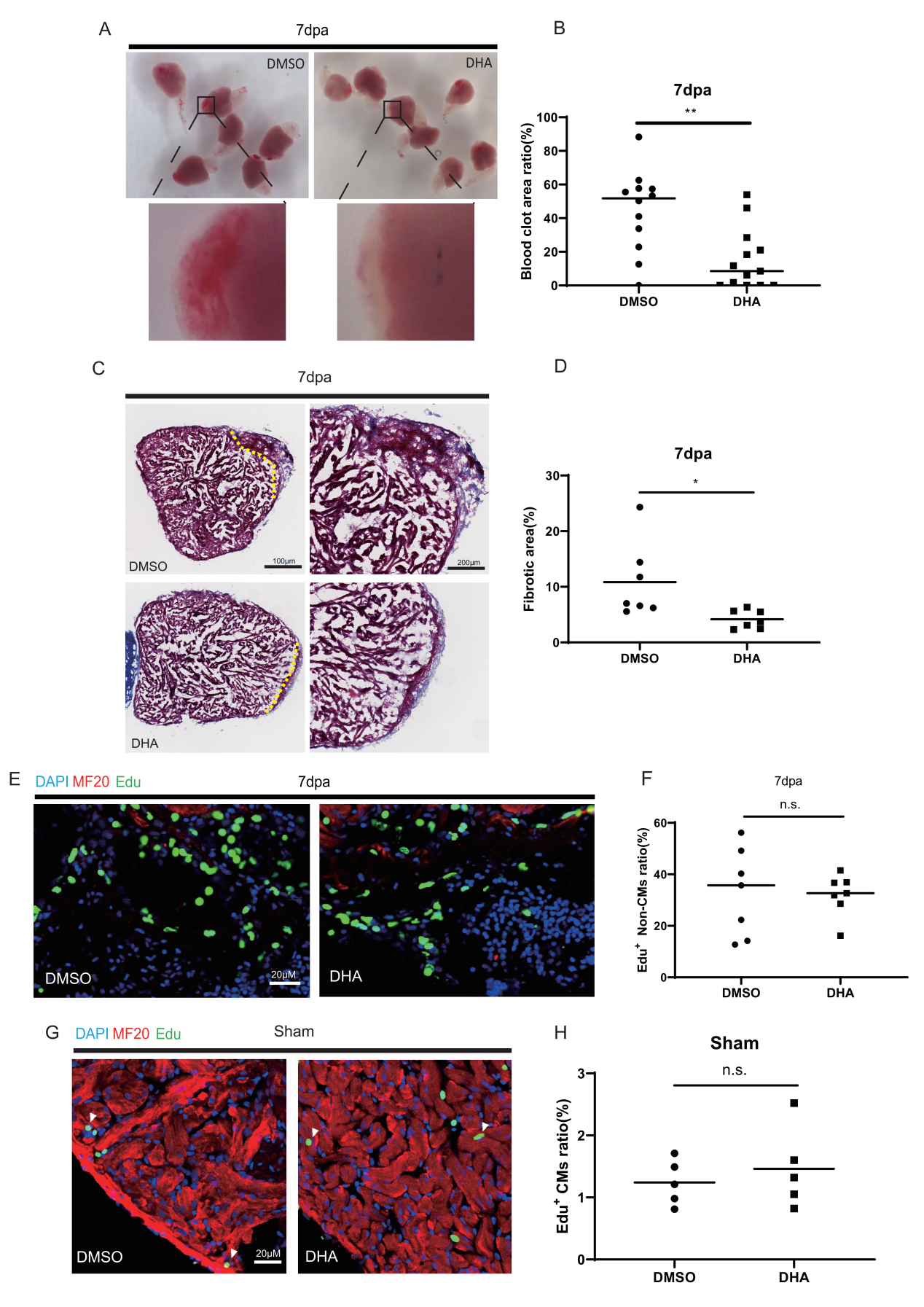
**

**Supplementary Figure 5. DHA injection promotes zebrafish heart regeneration.**

**A** Images showing blood clots in zebrafish injury hearts with DMSO or DHA injection at 7 dpa. Framed areas in upper panels were magnified in lower panels.

**B** Statistical analysis of blood clot areas in injury hearts in (**A**). Average area with blood clot on an injury heart was presented as the percentage of the total injury area (**A**). Each dot represents an individual heart. n: 12-13hearts/sample.

**C** Fibrin clot stained with Masson’s trichrome on the cryosections of zebrafish injury hearts injected with DMSO or DHA at 7 dpa. n: 7 hearts/sample; Scale bar: 200 μm.

**D** Statistical analyses of scar areas in in WT injury hearts injected with DMSO or DHA at 7dpa in (**C**).Average scar area with fibrin clots on heart sections was presented as the percentage of the total ventricular area (**C**). Each dot represents an individual heart.

**E** Immuno-staining of MF20 (in red) and EDU incorporation assay (in green) in injury areas of zebrafish WT injury hearts injected with DMSO or DHA at 7 dpa . Scale bar: 20 μm.

**F** Statistical analyses of EDU^+^ Non-CMs (**E**). The number of EDU^+^ Non-CMs on heart sections was presented as the percentage of the total cells without MF20 signals at the injury sites. Each dot represents an individual heart. n: 8-9 hearts/sample.

**G** Immuno-staining of MF20 (in red) and EDU incorporation assay (in green) of zebrafish WT Sham hearts injected with DMSO or DHA. Scale bar: 20 μm.

**H** Statistical analyses of EDU^+^ CMs in (**G**).

Statistical analysis was performed by Student’s two-tailed unpaired t test in GraphPad Prism 8. The p values were represented by n.s. and asterisks. n.s., p > 0.05; *, p < 0.05; **, p < 0.01.

**
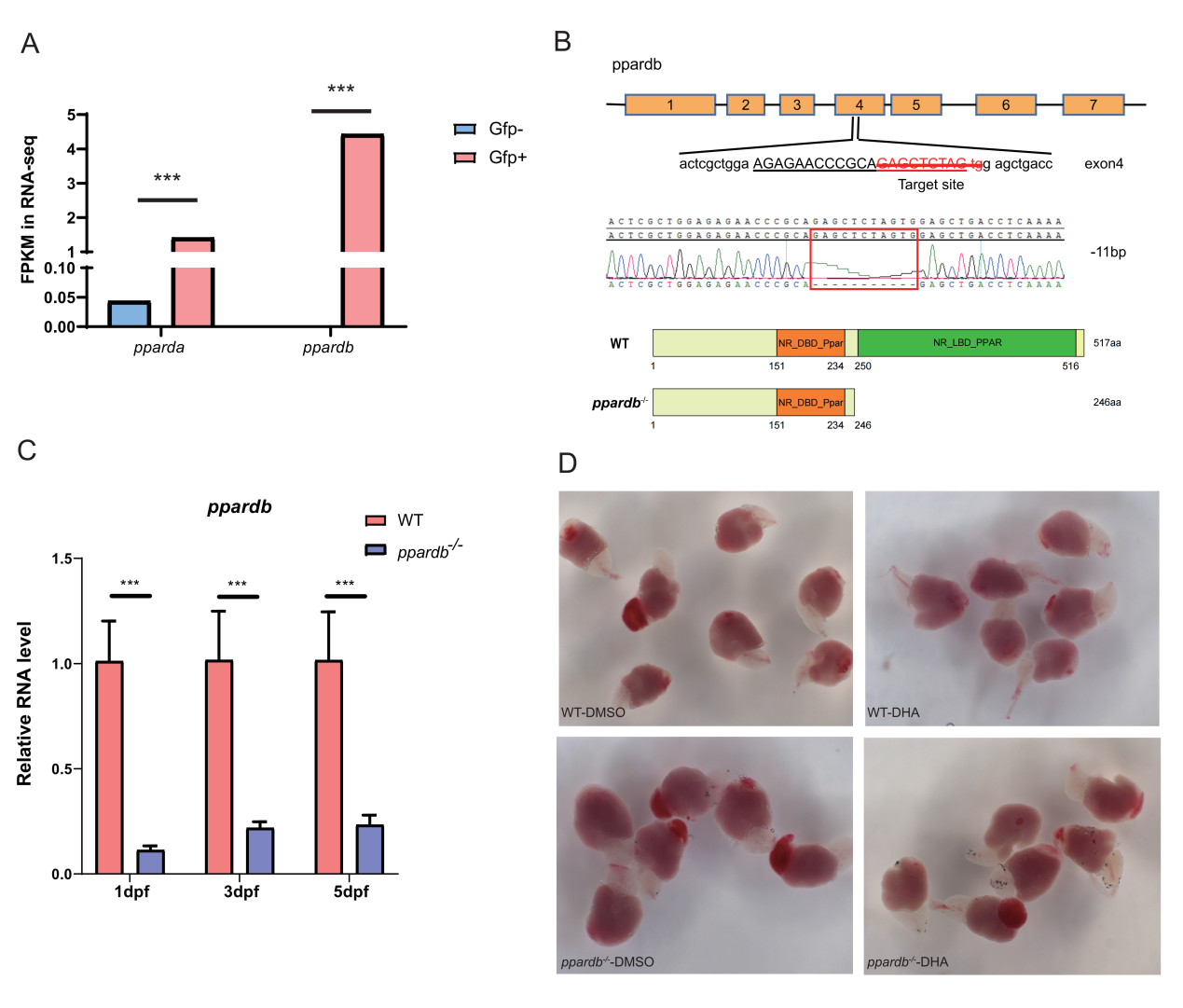
**

**Supplementary Figure 6. DHA promotes zebrafish heart regeneration in a *ppardb*-dependent manner.**

**A** FPKMs of *pparda* and *ppardb* in ∆113p53^+^ and ∆113p53^-^ CMs from RNA-seq.

**B** Diagram showing the gRNA targeting site and 11-bp deletion in the exon 4 of *ppardb* mutant, which results in a PTC at 246 aa.

**C** qRT-PCR was performed to exam the expression of *ppardb* in WT and *ppardb^-/-^* mutant embryos at 1, 3 and 5 dpf.

**D** Images showing blood clots in WT and *ppardb^-/-^* mutant injury hearts with DMSO or DHA injection at 7 dpa. Statistical analysis of blood clot areas in injury hearts was in **Figure 2H**. The experiment was repeated for three time. n: 11-12 hearts/sample.

Statistical analysis was performed by Student’s two-tailed unpaired t test in GraphPad Prism 8. The p values were represented by n.s. and asterisks. n.s., p > 0.05; *, p < 0.05; **, p < 0.01; ***, p < 0.001.

**
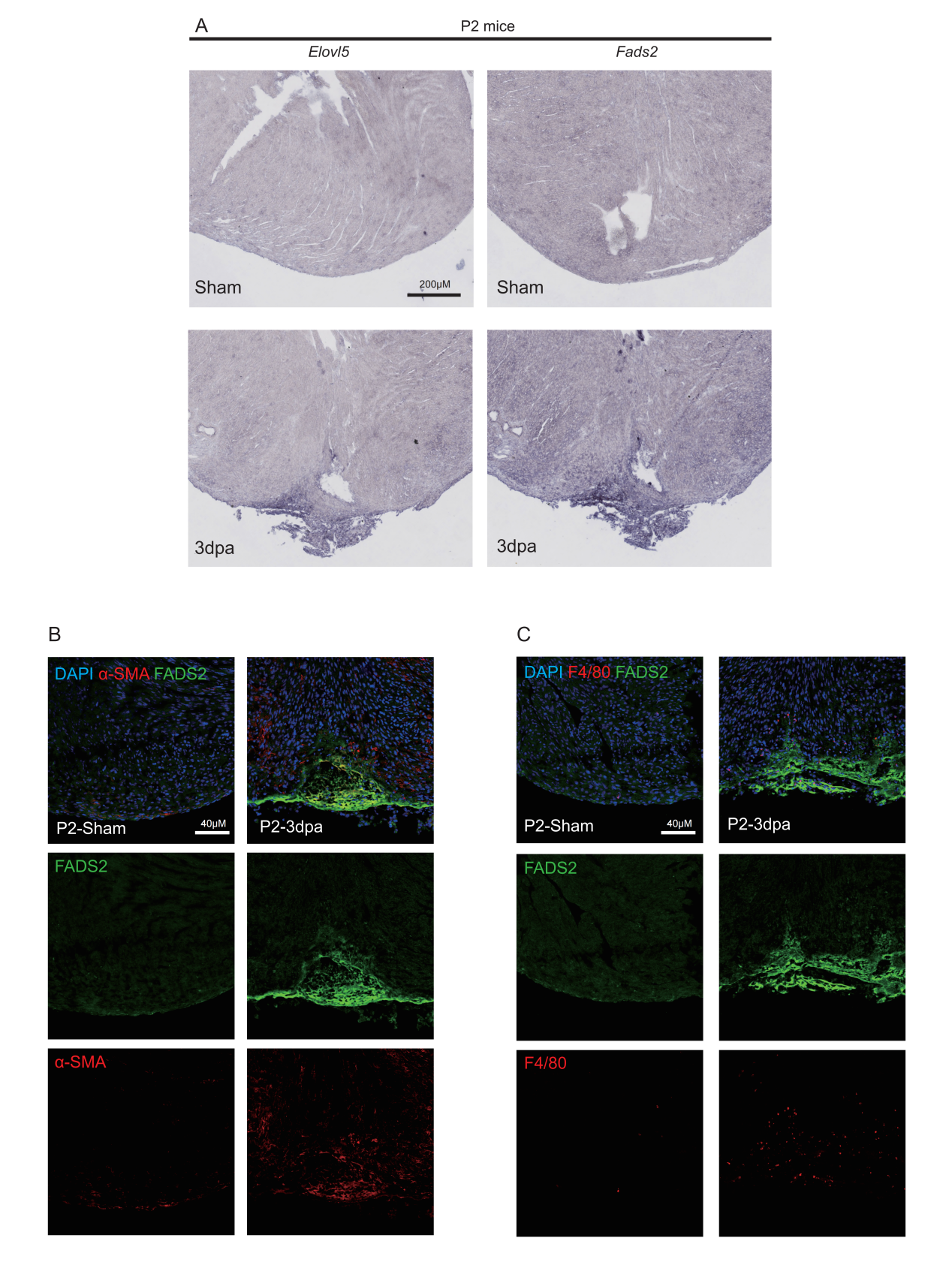
**

**Supplementary Figure 7.** ***Elov5* and *Fads2* encoding DHA synthesis enzymes were induced in injury areas of neonatal mouse hearts.**

**A** RNA in situ hybridization was performed with the DIG-labeled probe to detect both mouse *Elovl5* and *Fads2* on cryosections of neonatal mouse (P2) injury hearts at sham and 3 dpa. The representative picture was taken from 4-6 hearts in each group. Scale bar: 200 μm.

**B** Immuno-staining of α-SMA(a fibroblast marker) and FADS2 (in green) on cryosections of neonatal mouse (P2) injury hearts at sham and 3 dpa. The representative picture was taken from 4-6 hearts in each group. Scale bar: 200 μm.

**C** Immuno-staining of F4/80 (in red) (a macrophage marker) and FADS2 (in green) on cryosections of neonatal mouse (P2) injury hearts at sham and 3 dpa. The representative picture was taken from 4-6 hearts in each group. Scale bar: 200 μm.

**
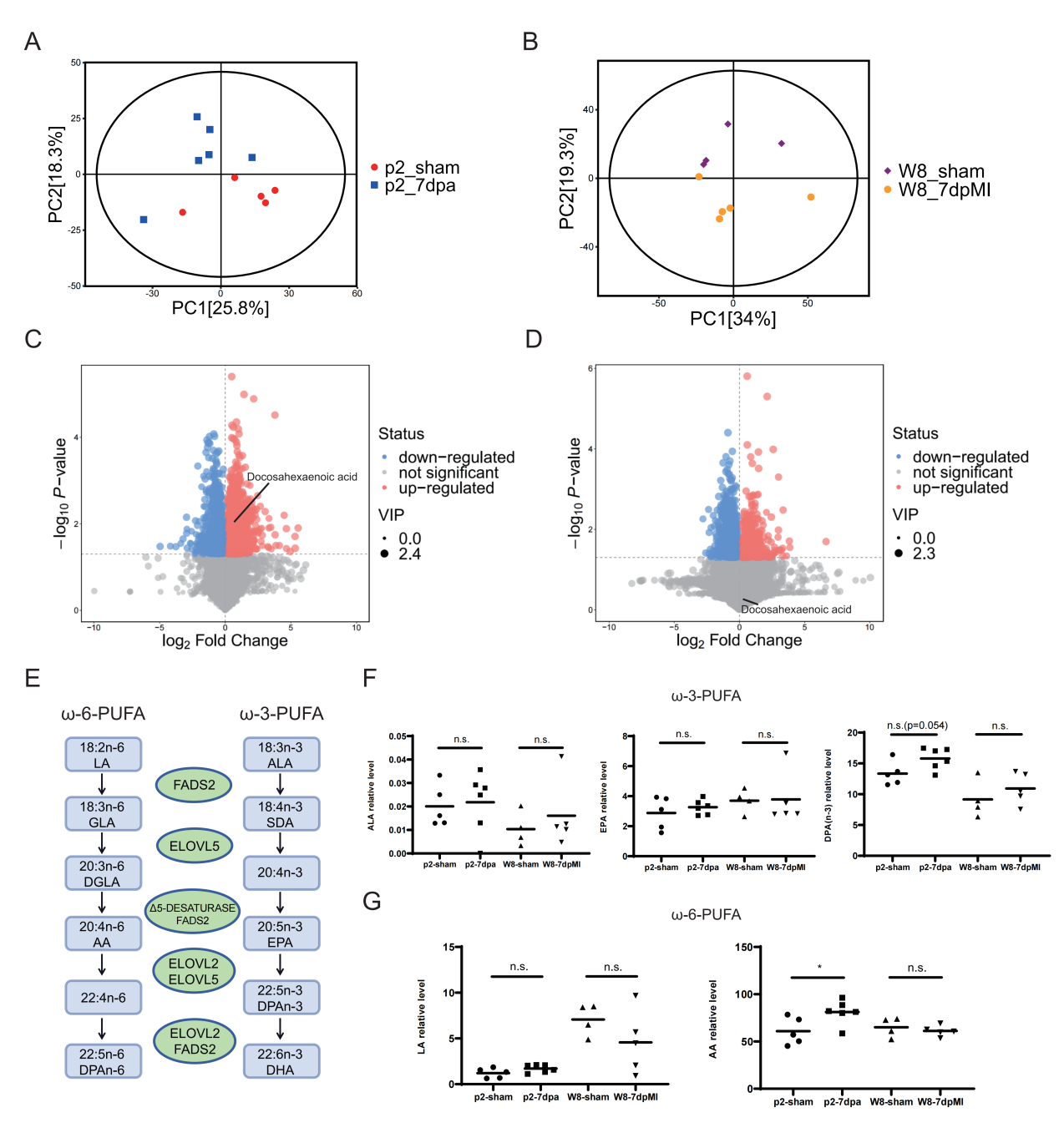
**

**Supplementary Figure 8. DHA accumulation is increased in injury hearts of neonatal mice, but not in injury hearts of adult mice.**

**A** PCA analysis. LC-MS data from 5-6 independent replicates including P2-neonatal mouse sham and injury hearts at 7 dpa, were subjected to PCA analysis. Each replicate consisted of 1 heart and subjected for LC-MS analysis.

**B** PCA analysis. LC-MS data from 4-5 independent replicates including W8-adult mouse sham and injury hearts at 7 dpMI, were subjected to PCA analysis. Each replicate consisted of 1 heart and subjected for LC-MS analysis.

**C** Volcano plot showing the differentially accumulated metabolites (VIP≥1, P-value< 0.05) in P2-neonatal sham hearts versus injury hearts at 7 dpa.

**D** Volcano plot showing the differentially accumulated metabolites (VIP≥1, P-value< 0.05) in W8-adult sham hearts versus injury hearts at 7 dpMI.

**E** Diagram of the process of ω-3 and ω-6 PUFA synthesis.

**F** Relative other ω-3 PUFA levels analyzed with LC-MS in neonatal mouse (P2) injury hearts at sham and 7 dpa, and adult mouse (W8) injury hearts at sham and 7 dpMI. Each treatment had 4-6 replicates. Each replicate had one injury heart.

**G** Relative ω-6 PUFA levels analyzed with LC-MS in neonatal mouse (P2) injury hearts at sham and 7 dpa, and adult mouse (W8) injury hearts at sham and 7 dpMI. Each treatment had 4-6 replicates. Each replicate had one injury heart.

Statistical analysis was performed by Student’s two-tailed unpaired t test in GraphPad Prism 8. The p values were represented by n.s. and asterisks. n.s., p > 0.05; *, p < 0.05.

**
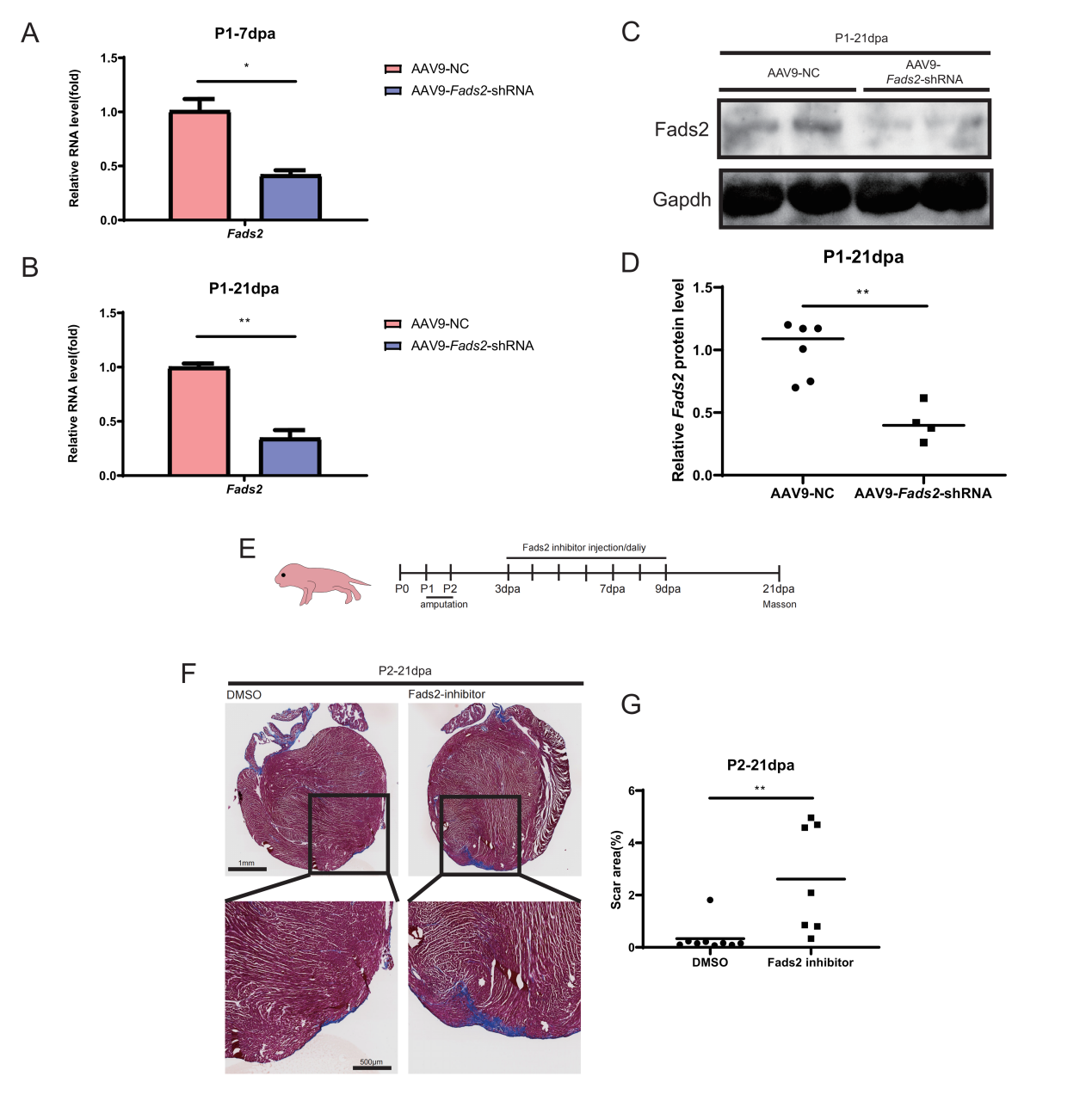
**

**Supplementary Figure 9. Inhibition of Fads2 impaired neonatal mice heart regeneration.**

**A,B** qRT-PCR was performed to exam the expression of *Fads2* in AAV-NC and AAV-*Fads2*-shRNA injected neonatal mice at 7(**A**), 21dpa(**B**).

**C,D** Western blot analysis was performed to exam the protein level of Fads2 in AAV-NC and AAV-*Fads2*-shRNA injected neonatal mice at 21dpa, n = 4-6 biological replicates.

**E** Diagram showing the time schedule of the chemical injection in P2 mice. The FADS2 inhibitor was intraperitoneally injected into neonatal mice with heart resection at P2 once daily for 7 days from 3 to 9 dpa.

**F** Fibrin clot stained with Massonth trichrome on the cryosections of injury hearts of P2 neonatal mice injected with DMSO or FADS2 inhibitor at 21 dpa. Framed areas in upper panels were magnified in lower panels. Scale bar: 1 mm or 500 00 bar: 1 mm or 500 dpa. rom 3 to 9 dpa. l

**G** Statistical analyses of scar areas in (**F**). Average scar area with fibrin clots on heart sections was presented as the percentage of the total ventricular area. Each dot represents an individual heart. n: 7-9 hearts/sample.

Statistical analysis was performed by Student’s two-tailed unpaired t test in GraphPad Prism 8. The p values were represented by n.s. and asterisks. n.s., p > 0.05; *, p < 0.05; **, p < 0.01.

**
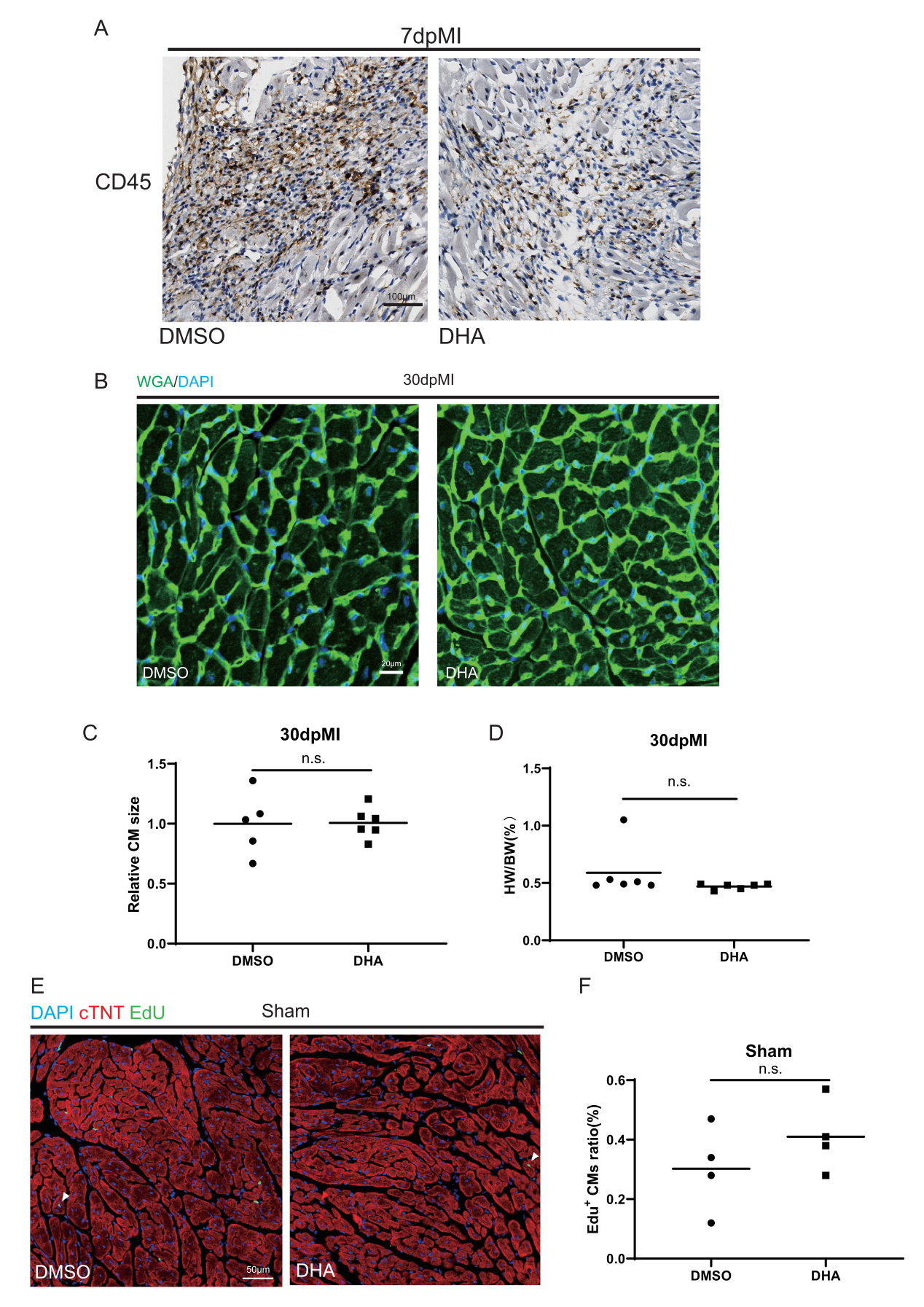
**

**Supplementary Figure 10.** **DHA injection down-regulates immune response, but has no obvious effect on CM hypertrophy in injury hearts and CM proliferation in sham hearts of adult mice.**

**A** Immunohistochemistry of CD45 (a pan leukocyte marker) around 7 dpMI infarcted CM areas of adult mouse hearts with DMSO or DHA injection. Scale bar: 100 μm.

**B** WGA staining of 30 dpMI adult mouse hearts with DMSO or DHA injection. Scale bar: 20 μm.

**C** Statistical analysis of relative CM sizes in (**B**). The relative CM sizes were calculated by dividing total area by the cell number in each heart section.

**D** Statistical analysis of the ratio of heart weight (HW) to body weight (BW) at 30 dpMI in MI treated adult mice with DMSO or DHA injection.

**E** Immuno-staining of CTNT (in red) and EDU incorporation assay (in green) of mouse sham hearts injected with DMSO or DHA. Scale bar: 50 μm.

**F** Statistical analyses of EDU^+^ CMs in (**E**).

Statistical analysis was performed by Student’s two-tailed unpaired t test in GraphPad Prism 8. The p values were represented by n.s. and asterisks. n.s., p > 0.05.

**
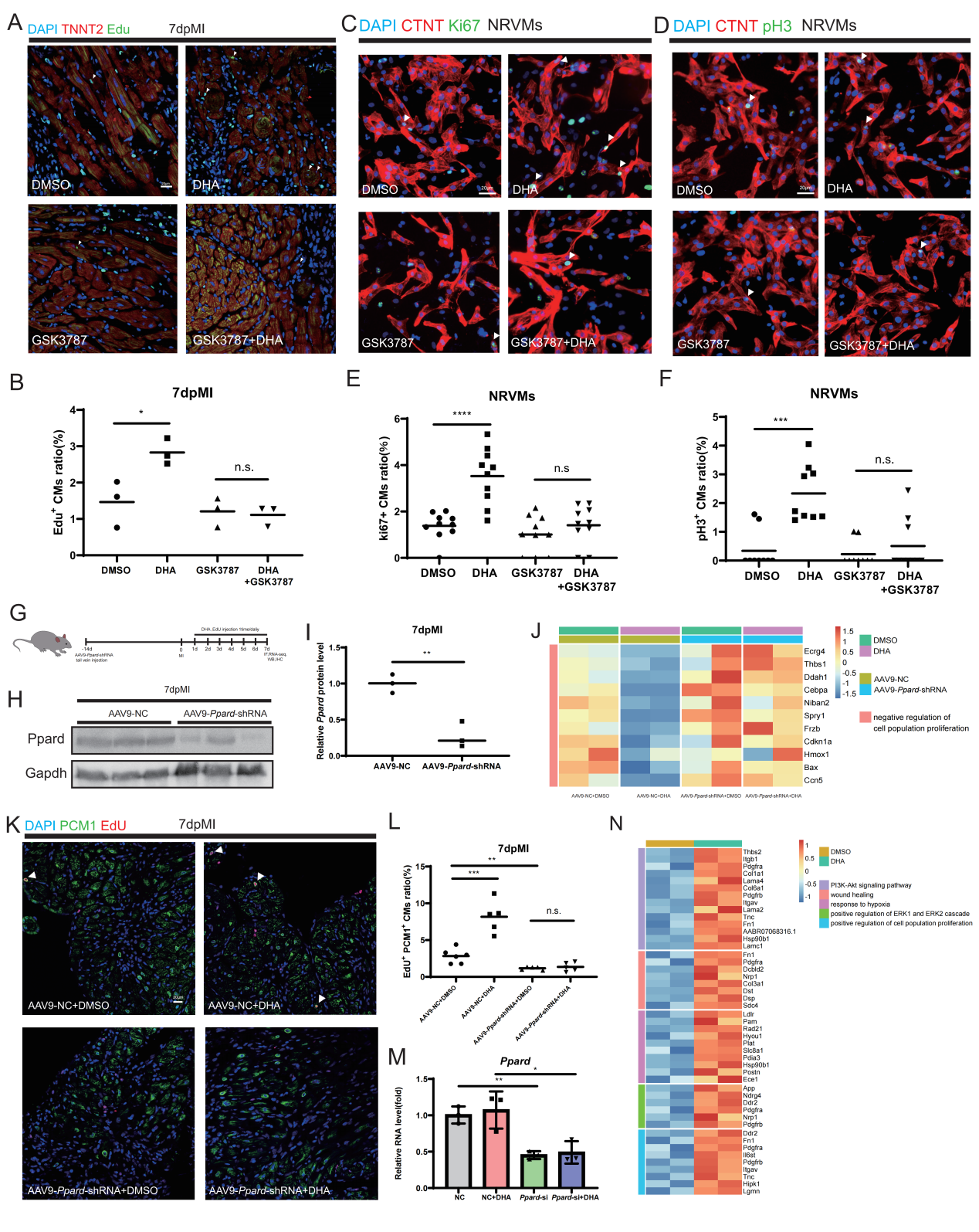
**

**Supplementary Figure 11. DHA injection promotes cardiomyocytes proliferation via PPARD in adult mice after MI and NRVMs.**

**A** Immuno-staining of TNNT2 (in red) and EDU incorporation assay (in green) of adult mouse MI hearts injected with DMSO or DHA or GSK3787 (a PPARD inhibitor) or DHA plus GSK3787 at 7dpMI as indicated. n: 3 hearts/sample; Scale bar: 20 μm.

**B** Statistical analyses of EDU^+^ CMs in (**A**). The numbers of EDU**^+^** CMs on heart sections were presented as the percentage of the total TNNT2^+^ cells at the injury sites respectively. Each dot represents an individual heart.

**C,D** Immuno-staining of CTNT (in red) and Ki67(**C**) or pH3(**D**) (in green) of NRVMs treated with DMSO or DHA or GSK3787 or DHA plus GSK3787 for 24h.. Scale bar: 20 μm.

**E,F** Statistical analyses of Ki67^+^ CMs in (**C**) or pH3^+^ CMs in (**D**) were presented as the percentage of the Ki67^+^ or pH3^+^ of the total CMs.

**G** Diagram showing the time schedule of the AAV9-*Ppard*-shRNA and chemical injection, as well as MI in adult mice.

**H,I** Western blot analysis was performed to exam the protein levels of PPARD in AAV-NC and AAV-*Ppard*-shRNA injected adult mouse hearts at 7dpMI. n = 3 biological replicates.

**J** Heatmap of downregulated negative regulators of cell proliferation only in DHA treated AAV9-NC injected injury hearts at 7dpMI.

**K** Immuno-staining of PCM1 (in green, nuclear marker of CMs) and EDU incorporation assay (in red) in adult mouse hearts with different treatments as indicated at 7dpMI. n: 5-6 hearts/sample. Scale bar: 20 μm.

**L** Statistical analyses of EDU^+^ PCM1^+^ cells.

Statistical analysis was performed by Student’s two-tailed unpaired t test in GraphPad Prism 8. The p values were represented by n.s. and asterisks. n.s., p > 0.05; *, p < 0.05; **, p < 0.01; ***, p < 0.001.

**M** qRT-PCR was performed to exam the mRNA levels of *Ppard* in *Ppard* siRNA or NC transfected NRVMs treated with DMSO or DHA for 24h.

**N** Heatmap of upregulated genes related to PI3-AKT, wound healing, response to hypoxia, positive regulation of ERK1 and ERK2 cascade, positive regulators of cell proliferation in DHA treated NRVMs.

Statistical analysis was performed by Student’s two-tailed unpaired t test in GraphPad Prism 8. The p values were represented by n.s. and asterisks. n.s., p > 0.05; *, p < 0.05; **, p < 0.01; ***, p < 0.001; ****, p < 0.0001.


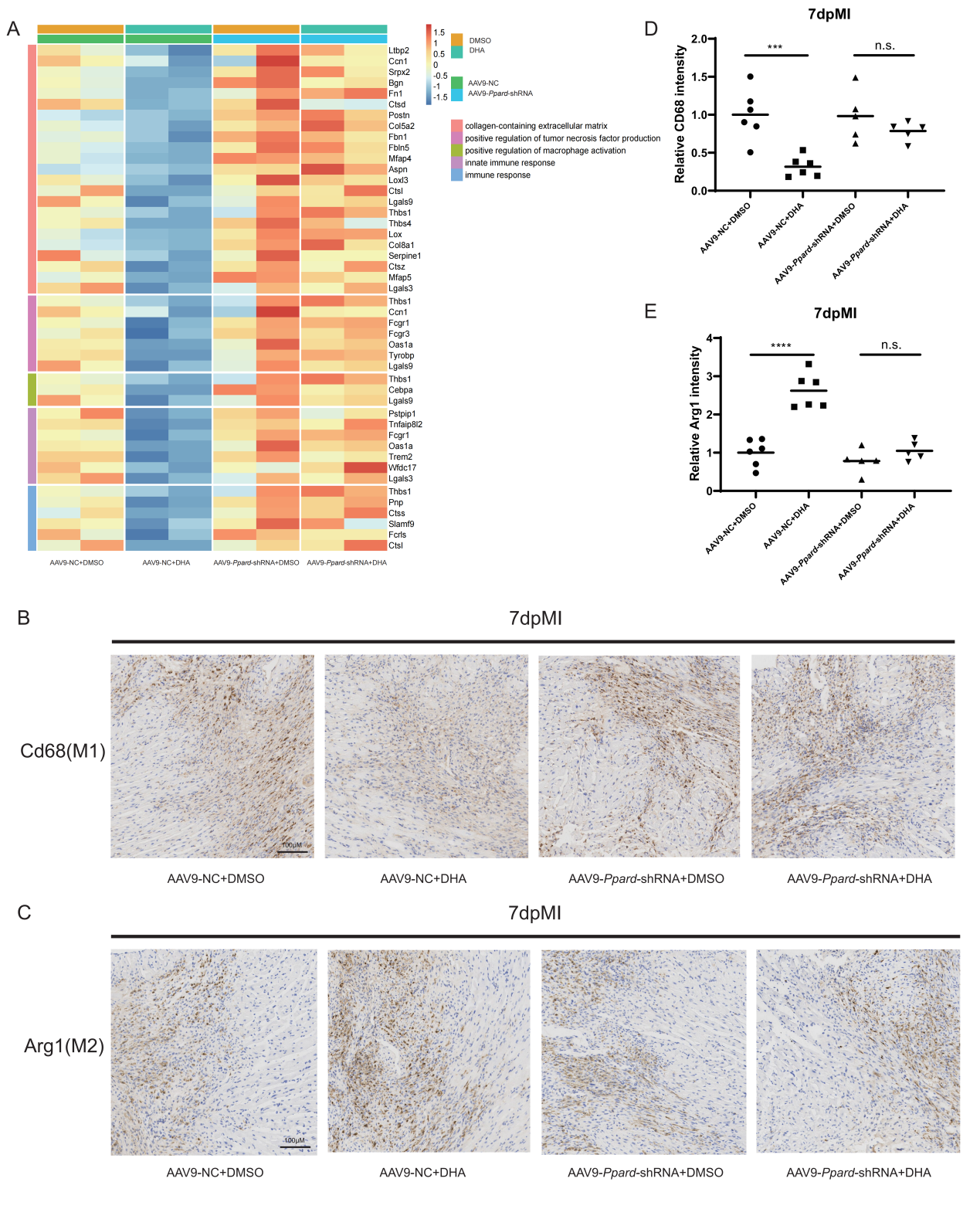


**Supplementary Figure 12. DHA supplementation inhibits immune responses and fibrosis in adult MI hearts through PPARD.**

**A** Heatmap of downregulated genes related to collagen-containing extracellular matrix, immune response, regulation of macrophage activation in DHA treated AAV9-NC injected injury hearts at 7dpMI.

**B,C** Immunohistochemistry of CD68 (M1 macrophage marker) and Arg1 (M2 macrophage marker) around injury areas of adult mouse hearts with different treatment as indicated at 7dpMI. Scale bar: 100 μm.

**D,E** Statistical analyses of relative intensity of CD68(**B**) or Arg1(**C**) expression in **b** and **c**.

Statistical analysis was performed by Student’s two-tailed unpaired t test in GraphPad Prism 8. The p values were represented by n.s. and asterisks. n.s., p > 0.05; *, p < 0.05; **, p < 0.01; ***, p < 0.001; ****, p < 0.0001.

**
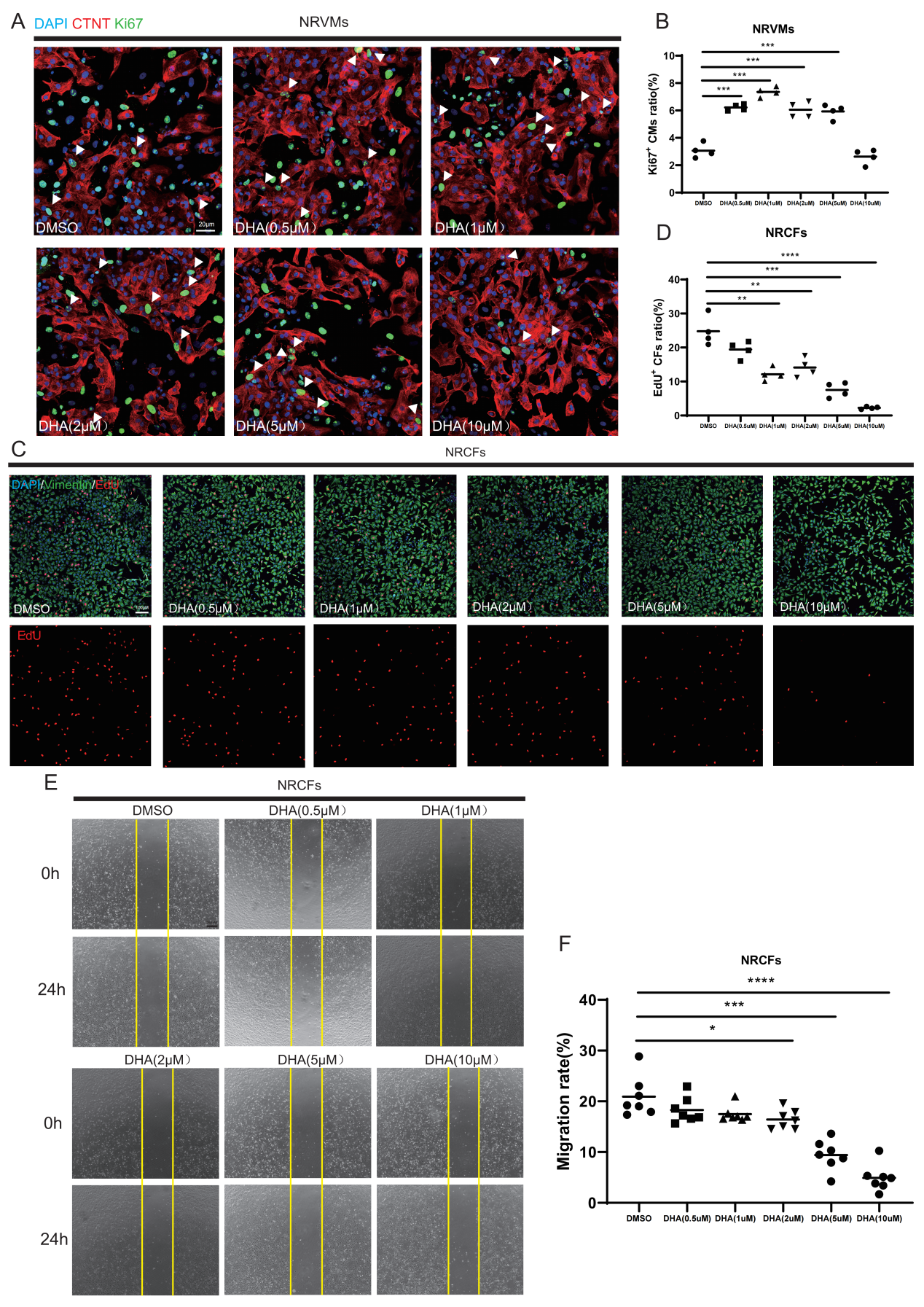
**

**Supplementary Figure 13.** **In contrast to NRCM, DHA inhibits NRCF proliferation and migration.**

**A** Immuno-staining of CTNT (in red) and Ki67 (in green) of NRVMs treated with different concentrations of DHA (0.5,1,2,5,10centfor 24h. Scale bar: 20 μm.

**B** Statistical analyses of Ki67^+^ CMs in (**A**).

**C** Immuno-staining of Vimentin (in green) and EdU incorporation assay (in red) of NRCFs treated with different concentrations of DHA (0.5,1,2,5,10centfor 24h.. Scale bar: 100 μm.

**D** Statistical analyses of EdU^+^ CFs in (**C**).

**E** Representative images in wound closure assay for cell migration capacity measurement of NRCFs treated with different concentrations of DHA (0.5,1,2,5,10μM) for 24h. Scale bars, 250 μm.

**F** Statistical analyses of the percentage of wound closure NRCFs in (**E**).

Statistical analysis was performed by Student’s two-tailed unpaired t test in GraphPad Prism 8. The p values were represented by n.s. and asterisks. n.s., p > 0.05; *, p < 0.05; **, p < 0.01.


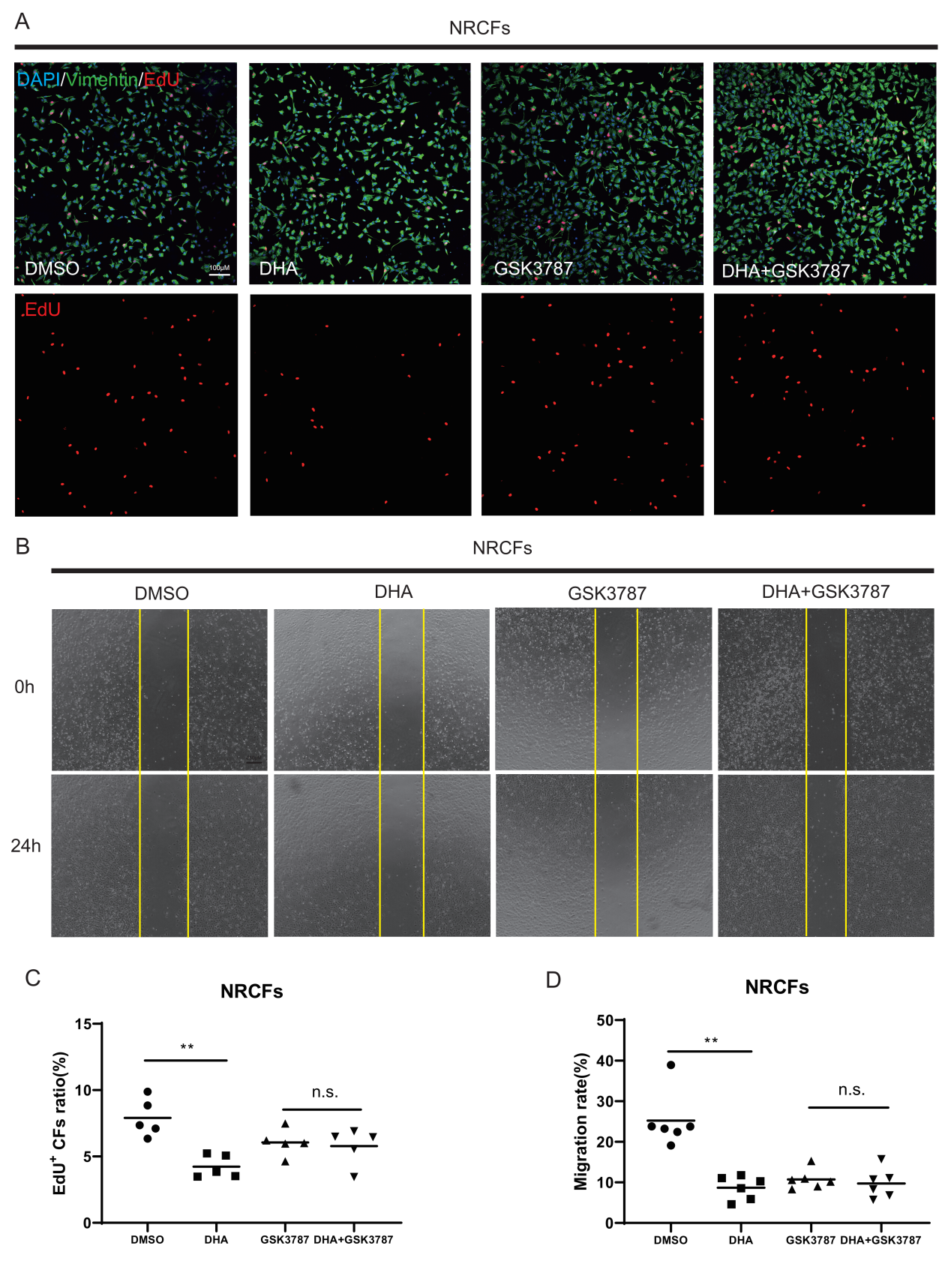


**Supplementary Figure 14. DHA inhibits NRCF proliferation and migration via PPARD.**

**A** Immuno-staining of Vimentin (in green) and EdU (in red) of NRCFs with different treatments as indicated for 24h. Scale bar: 100 μm.

**B** Statistical analyses of EdU^+^ CFs in (**A**).

**C** Representative images in wound closure assay for cell migration capacity measurement of NRCFs with different treatment as indicated for 24h. Scale bars, 250 μm.

**D** Statistical analyses of the percentage of wound closure NRCFs in (**B**).


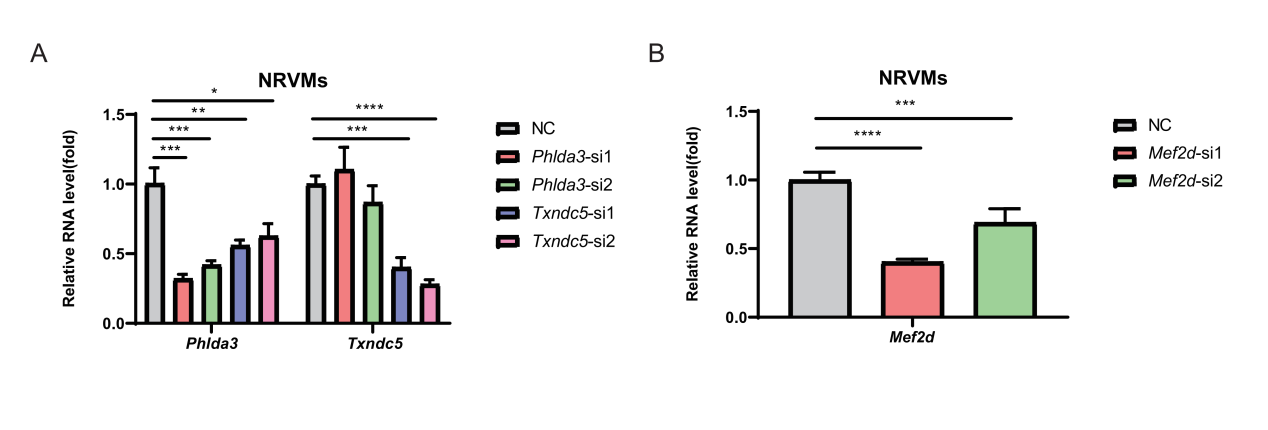


**Supplementary Figure 15.** **The mRNA level of *Phlda3*,** ***Txndc5* or *Mef2d* is significantly knocked down by its gene specific siRNAs in NRVMs.**

**A** qRT-PCR was performed to exam the mRNA level of *Phlda3* and *Txndc5* in NRVMs transfected with NC, *Phlda3* or *Txndc5* two siRNAs.

**B** qRT-PCR was performed to exam the mRNA level of *Mef2d* in NRVMs transfected with NC, *Mef2d* two siRNA.

**Supplementary table 1.**

| Primers for gRNA synthesis | | |
| --- | --- | --- |
| Gene | Forward | reverse |
| cpt1ab | ATAATACGACTCACTATAAGTGAGACTGGGCTATACTGGTTTTAGAGCTAGAAATAGC | AGCACCGACTCGGTGCCACT |
| ppardb | ATAATACGACTCACTATAGAGAACCCGCAGAGCTCTAGGTTTTAGAGCTAGAAATAGC | AGCACCGACTCGGTGCCACT |
| Primer for probes of in situ hybridization | | |
| Gene | Forward | reverse |
| aldocb | CACCAGTATCCCGCTCTCAC | TAATACGACTCACTATAgggGGAGTGGTGTCGCTGATCTT |
| pgm1 | GGCAGGCTAGTCATTGGTCA | TAATACGACTCACTATAgggTTCACGGCAGAGTTAGCAGG |
| hif1ab | TCCTCATCCCTCGAACATCG | TAATACGACTCACTATAgggGCAATTGAGGCTTTCTGGCT |
| ppardb | TGAGTTGAGCTCCGTCCCGATT | ATAATACGACTCACTATAgggCGCGCTCACATCGCTCGTACT |
| elovl5 | GAGATCTCAGGGTCACAGGATG | ATAATACGACTCACTATAgggGGCCGTAATACGAGTACATCAGC |
| fads2 | GGAAATACCTGAAGCCGCTGCT | ATAATACGACTCACTATAgggCGTCCGGGTCCTTCTTGAAGAT |
| cmlc2 | GCATAGATCAGAACCGGGATGG | ATAATACGACTCACTATAgggTTCCAGCCACGTCTATTGGAGC |
| Ms-elovl5 | GTCAAAGGATGGTTCCTCCTGG | ATAATACGACTCACTATAgggTGGTAGCGTGGTGGTAGACATG |
| Ms-fads2 | TTGGCCACTTAAAGGGTGCCTC | ATAATACGACTCACTATAgggGGCTCCCAAGATGCCGTAGAAA |
| Primers for qRT-PCR in zebrafish | | |
| aqp1a.1 | GCCCTGGGACTGAATCAAATC | GTCAGATGTCCCAGGCAAAC |
| prdx2 | GGATCAACACACCACGGAAG | ATCTGCCTCAAGATGCCCTT |
| xpo7 | CCTGATCCAGCTCTATGCCA | TGTAAGCGGATGTGTCGAGT |
| ap2m1a | CTGAGCGGAATGCCTGAATG | GCCATCAGGAGGGATGAAAC |
| plekhb2 | CTCACTCCTCCTGAAGGCAA | ATCTGAGGAGGAGCCACAAC |
| deptor | TGGACAGTGAAGCCAAAGTG | CTGCTCTGACTCATCCCTGT |
| itih1 | TGGAGACCATCCCTTAGCAA | CCAGAACTGGGTTGTCCATC |
| tuft1a | AGAGCCGAACATAAGCTGGT | TCTCTCTCTCCTCCTGGTGT |
| rgs5a | GGCCTGTGAGGACTTCAAGA | CCAGCTCAAAGGTGGAGGAA |
| cyp2ad3 | GGCCCTCGTTACTCAGAATG | GGAAGATGCACTCCTGTTGG |
| nob1 | CAGCAGATGCCATCACAGTC | GGGAGTAATCCACCCTCCAC |
| arpc1b | TTCTCAGGATCAACCGTGCT | GTGCCAGTCCAAACAAAGGA |
| oaz1a | CGGGATCACAGTCTTTCAGC | CCTTCTGGGAGGACACCAG |
| arrdc3a | GCACCTCAGGTCCAATTTCC | TGGCCACCAATTGCTTGATT |
| skp1 | GGGACCAGGAGTTCCTCAAA | CGCTTCCTCTTCCTCTGTGA |
| clic2 | ACAGTCCAAACAATGCTTTCCA | ACGTGCAGCTTTGGCAATAA |
| qkia | GAGAATTCTTGGCCCTCGTG | GTGTCTGTGTGTCCTCCACT |
| atp5f1b | CAGGCTAGTCCTGGAGGTG | TGGTCCTCTCTCGTCAATGG |
| rps6 | CTTCTACGAGAAGCGCATGG | CGAGGACGGTAACAAGAGTG |
| dusp1 | GACATGCTGGACATGTTGGG | TGGCAGTGAACAAAGACACG |
| zglut1a | TTGTTGGATCCTTCTCCGTCG | ACCCAGTGGACAGACCAGAA |
| mct4b | TCTCGTGGTGTTCTGCATCT | TAGTTCTTTGTGGCGTCCAGG |
| ldha | CGCACAAGATTGTAGCGGAT | ATGCAGTTGGGGCTGTACTT |
| pfkpa | GTGTTGATTCCCGAAATGCCC | ACGATCAATCGCACCTTCAG |
| acat2 | ATCTGGCCTGAAGTCTGTGTG | GTGTCCTGCAGAGTAGCATCT |
| cpt1ab | ATGATGGATGAGAAAGCTAC | TTCAAGTCAAAGAGTTCCAT |
| cmyc | GGATCTGAGCACCTCTGCAT | CGACTCTGAAGCATCCGTCT |
| hif1aa | CAGCGTCACCTCTAACCTGG | AACCCGTCCAGAGCTTTCAG |
| mct1 | CACCGATTACTTGGGCTTTGC | GCAATCACCAGAACGATTCCA |
| rpe | GACCAGAGCAGTGGGTGAAA | GCCCATGGTGCCAATTCTTC |
| rpia | TCCTGAGCTGGATGTAGCGA | GACACAGGCACATACGCCAT |
| aldocb | AGGAGAACCGCCGTCTTTAC | TCTCTCCGTTTGTTCCAGCC |
| pgm1 | TGCGTGGCTCTCAATACTGG | ACGTTTTATCCCCCGAGGTG |
| lsm12b | AGTTGTCCCAAGCCTATGCAATCAG | CCACTCAGGAGGATAAAGACGAGTC |
| rpl32 | TGACCGCTATGTCAAGATCAGG | TTGAGGACACGTTGTGAGCG |
| ∆113p53 | ATATCCTGGCGAACATTTGGAGGG | CCTCCTGGTCTTGTAATGTCAC |
| EGFP | GCACCATCTTCTTCAAGGAC | TCGTTGGGGTCTTTGCTCAG |
| cdc6 | CGGCTTGTCTGAACTGTGTG | CTCCAATCGTGCCTGACTGT |
| wee1 | TACTGGTGCAGTCATCGACAC | CGTCCAACCTCTTGACGCAT |
| ppardb | GCGAAGTTTACGCAAGCCAT | GAGTCCGGATGGTGAACCTG |
| gapdh | TTCCAGTACGACTCCACCCA | TGACTCTCTTTGCACCACCC |
| β-actin | CATTGGCAATGAGCGTTTC | TACTCCTGCTTGCTGATCCAC |
| Primers for qRT-PCR in NRVMs | | |
| Mef2d | GGGTGGAGACCTCAACAGTG | CTTGGCAGGGATGACCTTGT |
| Txndc5 | AGCCTGGACAAGAAGCAGTG | TGCAGCTCAAAGTTGTTGGC |
| Phlda3 | ATGCCAGCTTCTCTGTCCAC | GTAAGGCCCAAGTGCTCCAT |
| β-actin | TGGCACCACACTTTCTACAATGAGC | GGGTCATCTTTTCACGGTTGG |
| Ppard | ATTCCTCCCCTTCCTCCCTG | TCCAAAGCGGATAGCGTTGT |
| Primers for qRT-PCR in mice | | |
| Ms-Fads2 | TTCTACGGCATCTTGGGAGC | GGCATAGTGGGGAAGAGGTG |
| Ms-Gapdh | CATCACTGCCACCCAGAAGACTG | ATGCCAGTGAGCTTCCCGTTCAG |
| Primers for siRNA synthesis in NRVMs | | |
| Gene | sense | anti-sense |
| Mef2d-siRNA1 | CAGUUCAGCAAUCCAAGUATT | UACUUGGAUUGCUGAACUGTT |
| Mef2d-siRNA2 | CGUGGCAACACCAAGUUUATT | UAAACUUGGUGUUGCCACGTT |
| Phlda3-siRNA1 | UAAAGGAGGGCGUGCUGGATT | UCCAGCACGCCCUCCUUUATT |
| Phlda3-siRNA2 | GUCAAGUUCAAGAAUCAACTT | GUUGAUUCUUGAACUUGACTT |
| Txndc5-siRNA1 | GGGUCCUAGAGACUUUGAATT | UUCAAAGUCUCUAGGACCCTT |
| Txndc5-siRNA2 | CCUCGAACAUUCUGAAACUTT | AGUUUCAGAAUGUUCGAGGTT |
| Ppard-siRNA1 | GGAAGAGGAGAAAGAGGAATT | UUCCUCUUUCUCCUCUUCCTT |
| Ppard-siRNA2 | GCAAGAUCCAGAAGAAGAATT | UUCUUCUUCUGGAUCUUGCTT |

**Supplementary table 2.**

| **REAGENT or RESOURCE** | **SOURCE** | **IDENTIFIER** |
| --- | --- | --- |
| **Antibodies** | | |
| anti-PPARD | Santa cruz | Cat# sc-74517 |
| anti-Ki67 | ABclonal | Cat# A23722 |
| anti-MF20 | Developmental Studies Hybridoma Bank | Cat# AB2147781 |
| anti-PH3 | Santa cruz | Cat# sc-8656-R |
| anti-TNNT2 | ABclonal | Cat# A4914 |
| anti-CTNT | ThermoFisher | Cat# MA5-12960 |
| anti-CD45 | Servicebio | Cat# GB11066 |
| anti-FADS2 | Affinity Biosciences | Cat#DF15514 |
| anti-PCM1 | Sigma-Aldrich | Cat#HPA023374 |
| anti-GAPDH | Proteintech | Cat#60004-1-Ig |
| anti-mouse IgG H&L Alexa Fluor 488 | Abcam | Cat# ab150113 |
| anti-mouse IgG H&L Alexa Fluor 647 | Abcam | Cat# ab150115 |
| anti-Rabbit IgG H&L Alexa Fluor 647 | Abcam | Cat#ab150143 |
| anti-rabbit IgG-HRP | Servicebio | Cat# GB23303 |
| goat anti-rabbit IgG | HUABIO | Cat#HA1001 |
| goat anti-mouse IgG | HUABIO | Cat#HA1006 |
| Anti-Digoxigenin-AP | Roche | Cat# 11093274910 |
| Anti-Fluorescein-AP | Roche | Cat# 11426338910 |
| **Chemicals, peptides, and recombinant proteins** | | |
| DAPI | BYT | Cat# C1002 |
| Etomoxir | MCE | Cat# HY-5020 |
| Baicalin | MCE | Cat# HY-N0197 |
| DHA | MCE | Cat# HY-B2167 |
| sc-26196 | MCE | Cat# HY-107410 |
| GSK3787 | MCE | Cat#HY-15577 |
| OA | Solarbio | Cat#O8291 |
| EdU | Invitrogen | Cat# A10044 |
| Azide Alexa Fluor 647 | Invitrogen | Cat# A10277 |
| Oil Red O solution | sigma | Cat# 01516 |
| redyeing solution | Nanjing Jiancheng technology | Cat# D207-1-3 |
| Wheat Germ Agglutinin, Oregon Green 488 Conjugate | Invitrogen | Cat# W6748 |
| **Critical commercial assays** | | |
| Bacillus Licheniformis protease | Creative Enzymes | Cat# NATE0633 |
| TRIZOL reagent | AidLab | Cat# RN0102 |
| DNase I | NEB | Cat# M0303S |
| M-MLV Reverse Transcritptase | Invitrogen | Cat# 28025021 |
| T7 RNA Polymerase | NEB | Cat# M0251S |
| DIG RNA Labelling Mix | Roche | Cat# 11277073910 |
| BCIP/NBT Alkaline Phosphatase Colour Development Kit | Beyotime Biotechnology | Cat# C3206 |
| Fast Red TR/Naphthol AS-MX Tablets | SIGMAFAST | Cat#1003318421 |
| Luciferase Reporter Gene Assay Kit | Yeasen | Cat# 11401ES76 |
| **Software and algorithms** | | |
| CFX96TM Real-Time System/C1000 Thermal Cycle | BIORAD | N/A |
| ImageJ | NIH | https://imagej.nih.gov/ij/index.html |
| FastQC | Babraham Bioinformatics | https://www.bioinformatics.babraham.ac.uk/projects/fastqc/ |
| Hisat2 | Tophat | https://daehwankimlab.github.io/hisat2/ |
| Bowtie2 | Johns Hopkins University | https://bowtie-bio.sourceforge.net/bowtie2/index.shtml |
| Samtools | Github | https://github.com/samtools/samtools |
| Picard | Broad Institute | https://broadinstitute.github.io/picard/ |
| deepTools | Max Planck Institute for Immunobiology and Epigenetics, Freiburg | https://test-argparse-readoc.readthedocs.io/en/latest/ |
| featureCounts | Subread | https://subread.sourceforge.net/ |
| MACS2 | Github | https://pypi.org/project/MACS2/ |
| HOMER | UC San Diego | http://homer.ucsd.edu/homer/ |
| DAVID | Laboratory of human retrovirology and immunoinformatics (LHRI) | https://david.ncifcrf.gov/ |
| R | The Comprehensive R Archive Network | https://cran.r-project.org/ |
| DESeq2 | Bioconductor | https://bioconductor.org/packages/release/bioc/html/DESeq2.html |
| ChIPseeker | Bioconductor | https://bioconductor.org/packages/release/bioc/html/ChIPseeker.html |
| GSEA | UC San Diego and Broad Institute | https://www.gsea-msigdb.org/gsea/index.jsp |
| IGV | UC San Diego and Broad Institute | https://igv.org/ |
| GraphPad Prism 8 | GraphPad Software | https://www.graphpad.com/features |
| **Deposited data** | | |
| RNA-seq data | This paper | NCBI BioProject: PRJNA938172, PRJNA1172169, PRJNA1251510 |
| Chip-seq data | This paper | NCBI BioProject: PRJNA1172169 |
